# Cs_2_CO_3_-promoted defluorination and functionalization of α-CF_3_ carbonyl compounds in the presence of *N*-, *O*-, and/or *S*-nucleophiles†

**DOI:** 10.1039/c8ra02353k

**Published:** 2018-04-30

**Authors:** Yue Wu, Bingbing Zhang, Yinying Zheng, Yuheng Wang, Xinsheng Lei

**Affiliations:** School of Pharmacy, Fudan University 826 Zhangheng Road, Pudong Zone Shanghai 201203 China leixs@fudan.edu.cn +86 21 5198 0128 +86 21 5198 0128

## Abstract

A simple, efficient, and mild method for defluorination and functionalization of 3,3,3-trifluoro carbonyl compounds has been developed. In the present method, Cs_2_CO_3_ can easily convert α-trifluoromethyl esters, amides, and ketones into β,β-*S*-, *O*- and/or *N*-substituted α,β-unsaturated carbonyl compounds in the presence of *N*-, *O*-, and *S*-nucleophiles with moderate to excellent yields, and furthermore, this transformation with α-trifluoromethyl ester and a series of 2-aminophenols can result in benzooxazoles in good yields.

## Abstract 2

Cs_2_CO_3_-promoted defluorination and functionalization of α-CF_3_ carbonyl compounds.

## Graphical abstract

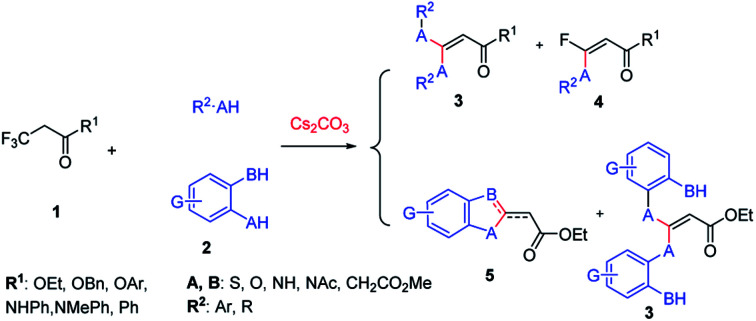

## Introduction

In the past six decades, hydrofluorocarbons have been widely used in pharmaceuticals, agrochemicals, materials, refrigeration, and air conditioning. Introduction of C–F bonds into pharmaceuticals or materials can modify the acidity, lipophilicity, conformation and metabolism of pharmaceuticals,^1–3^ improve the hydrophobic properties, chemical inertness, and elasticity of materials,^4,5^ and sometimes enhance their special water-/stain-resistant and non-sticky characteristics.^6,7^ This “fluorine effect” or “fluorine magic” stems from a very short bond length, low polarizability, being fairly inert and the strong inductive effects of the C–F bond.^8,9^

Logically, chemical inertness of C–F bond will make hydrofluorocarbons resistent to biotransformation or biodegradation.^10,11^ Thus, continual and increasing use of hydrofluorocarbons in modern life has implications for the environment and human health that urgently require attention.^12,13^ In light of the environmental concerns associated with organofluorine compounds,^14,15^ the development of novel synthetic methods for the C–F cleavage and subsequent functionalization is highly required.^16^

**Fig. 1:**
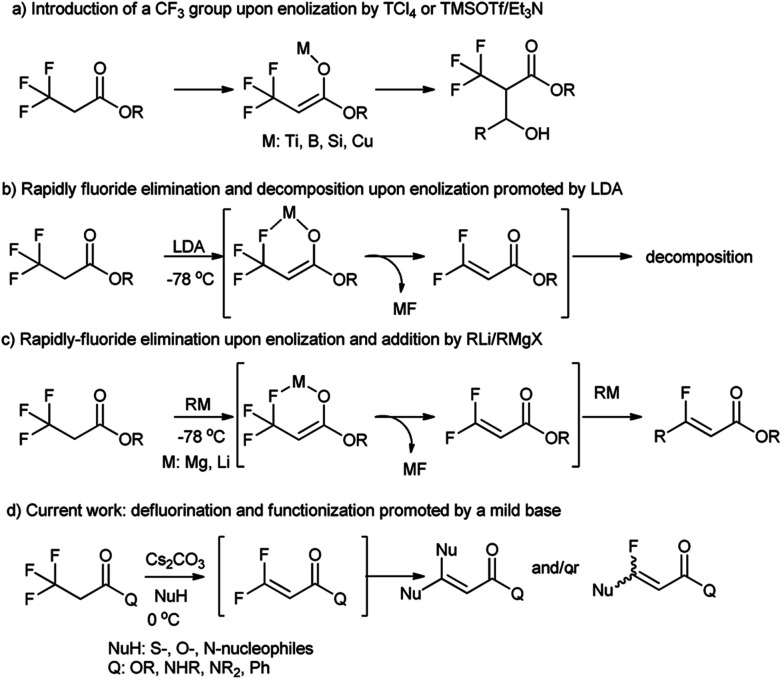
The enolization and defluorination of 3,3,3-trifluoropropanoic acid derivatives.

3,3,3-Trifluoropropanoic acid derivatives are one of the very important building blocks to incorporate one CF_3_ group into organic molecules. The exploitation of their α-CF_3_ enolates as active nucleophiles for the introduction of a CF_3_ group have been achieved (Fig. 1, path a),^17–21^ but their defluorination and functionization are only scarcely explored,^22^ because their direct defluorinations usually require strong basic conditions^23^ and the resultant β,β-difluoro-α,β-unsaturated carbonyl compounds are prone to subsequent decomposition even at a low temperature (path b).^24^ Recently, defluorination and functionization of 3,3,3-trifluoropropanoic acid derivatives have been reported at −78 °C to prepare monofluoroalkenes *via* elimination and addition with organolithium or Grignard reagents as *C*-nucleophiles (path c).^25^ These results prompted us to examine defluorination and functionization of α-CF_3_ carbonyl compounds with other nucleophiles such as *S*-, *O*-, and *N*-nucleophiles. Herein, we described an efficient and mild defluorinated and functionized method of α-CF_3_ carbonyl compounds *via* a Cs_2_CO_3_-promoted elimination/addition in the presence of a series of *N*-, *O*-, *S*-nucleophiles.

## Results and discussion

We initiated our work by studying the reaction of ethyl 3,3,3-trifluoropropanoate (1a) and 4-mercaptotoluene (2a) under the basic conditions. As shown in Table 1, in the presence of NaHMDS, the reaction was run at rt for 2 h, and expectedly, 2a was not consumed because of the almost quantitative recovery of 2a. When the reaction was run at 0 °C, only a small amount of defluorinated product could be isolated (entry 1, 37%), and its structure was subsequently determined as 3aa. Decreasing reaction temperature to −78 °C, only 3aa could be isolated in a higher yield (entry 2, 46%), but the partial-defluorinated product, such as 4aa, was still not isolated. Increasing the amount of 2a did not improve the reaction (entry 3, 47%), instead, increasing the amount of NaHMDS could increase the yield of 3aa up to 88%, albeit without 4aa. LDA had a similar performance to NaHMDS (entry 5, 64%). The above results implied that those bases might rapidly transform the ester into active β,β-difluoro-α,β-unsaturated carbonyl compounds, and then this unstable intermediate could be trapped by 2a to some extent while it was prone to decompose.

**Table 1:** The optimization on the reaction condition for the defluorination and functionization of 3,3,3-trifluoropropanoic acid derivatives

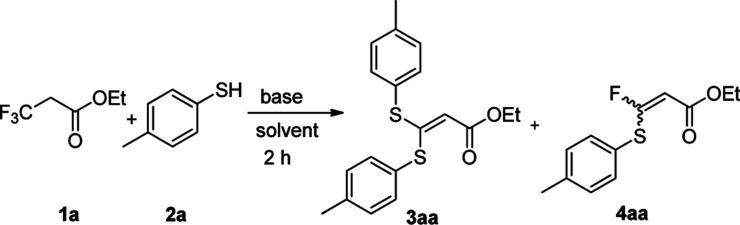					
Entry	1a/2a/base (eq.)	Base	Temp.	Solvent	Yield (3aa)^a^^,^^b^
1	1.0/1.0/2.0	NaHMDS	0 °C	THF	37%
2	1.0/1.0/2.0	NaHMDS	−78 °C	THF	46%
3	1.0/2.0/2.0	NaHMDS	−78 °C	THF	47%
4	1.0/2.0/3.0	NaHMDS	−78 °C	THF	88%
5	1.0/1.0/3.0	LDA	−78 °C	THF	64%
6	1.0/2.0/3.0	*t*-BuOK	0 °C	THF	54%
7	1.0/2.0/3.0	MeONa	0 °C	THF	56%
8	1.0/2.0/3.0	Na_2_CO_3_	0 °C	THF	ND^c^
9	1.0/2.0/3.0	K_2_CO_3_	0 °C	THF	15%
10	1.0/2.0/3.0	AcONa	0 °C	THF	ND^c^
11	1.0/2.0/3.0	NEt_3_	0 °C	THF	ND^c^
12	1.0/2.0/2.0	Cs_2_CO_3_	0 °C	THF	96%
13	1.0/2.0/2.0	Cs_2_CO_3_	0 °C	DMF	30%
14	1.0/2.0/2.0	Cs_2_CO_3_	0 °C	DMSO	74%
15	1.0/2.0/2.0	Cs_2_CO_3_	0 °C	EtOH	ND^c^
16	1.0/2.0/2.0	Cs_2_CO_3_	25 °C	THF	43%

a Reaction conditions: to a solution of 1a (1.0 mmol), 2a in anhydrous THF (10 mL) was added one kind of base in one portion at the suitable temperature under Ar, and the reaction mixture was stirred for the indicated time.

b The isolated yield based on 1a.

c Not detected.

Based on the facts that only the trace partial-defluorinated product was observed and the major full-defluorinated product, as one kind of α-oxoketene-*S*,*S*-acetals, might be one potentially versatile three-carbon building blocks for the construction of various heterocyclic systems,^26^ we turned to optimize the full-defluorinated reaction by screening a variety of other bases for the reaction.

As shown in Table 1, *t*-BuOK gave a moderate yield (entry 6, 54%) at 0 °C, but a higher or lower temperature led to a poorer yield (at rt or −78 °C). Similarly, MeONa could give the almost same result (entry 7, 56%). The weaker bases, such as Na_2_CO_3_, AcONa and Et_3_N, could not initiate the reaction (entry 8, 10, 11) while K_2_CO_3_ just resulted in a very low yield (entry 9, 15%). To our delight, Cs_2_CO_3_ gave the almost quantitative yield and its amount could be reduced from 3.0 to 2.0 equivalents (entry 12, 98% *vs.* 96%). Subsequently, the solvent screening demonstrated that neither DMF nor EtOH was good to the reaction (entry 13, 15), and DMSO just gave a slightly decreased yield (entry 14, 74%). As a result, THF was chosen as the optimized solvent for the reaction, but therein, elevating the temperature seemed to have an adverse effect on the reaction (entry 16).

With the optimized condition in hand, we subsequently examined the nucleophile scope with a variety of mono-dentate *S*-, *O*-, and *N*-nucleophiles, and the results were depicted in Table 2. For the *S*-containing nucleophiles, aryl thiols generally gave their defluorinated products in good to excellent yields (3aa–3af, 46–96%), and the substituent electronic effect in the aryl ring may have little influence on the reaction, instead, the bulkier nucleophile seems to have a slight effect on the reaction (3ac, 46%). This reaction could successfully be expanded to the aliphatic thiol (3ag, 89%).

**Table 2:** The substrate scope of defluorination and functionization of 3,3,3-trifluoropropanoic acid derivatives with mono-dentate nucleophiles

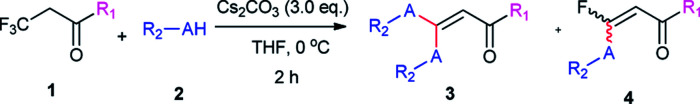
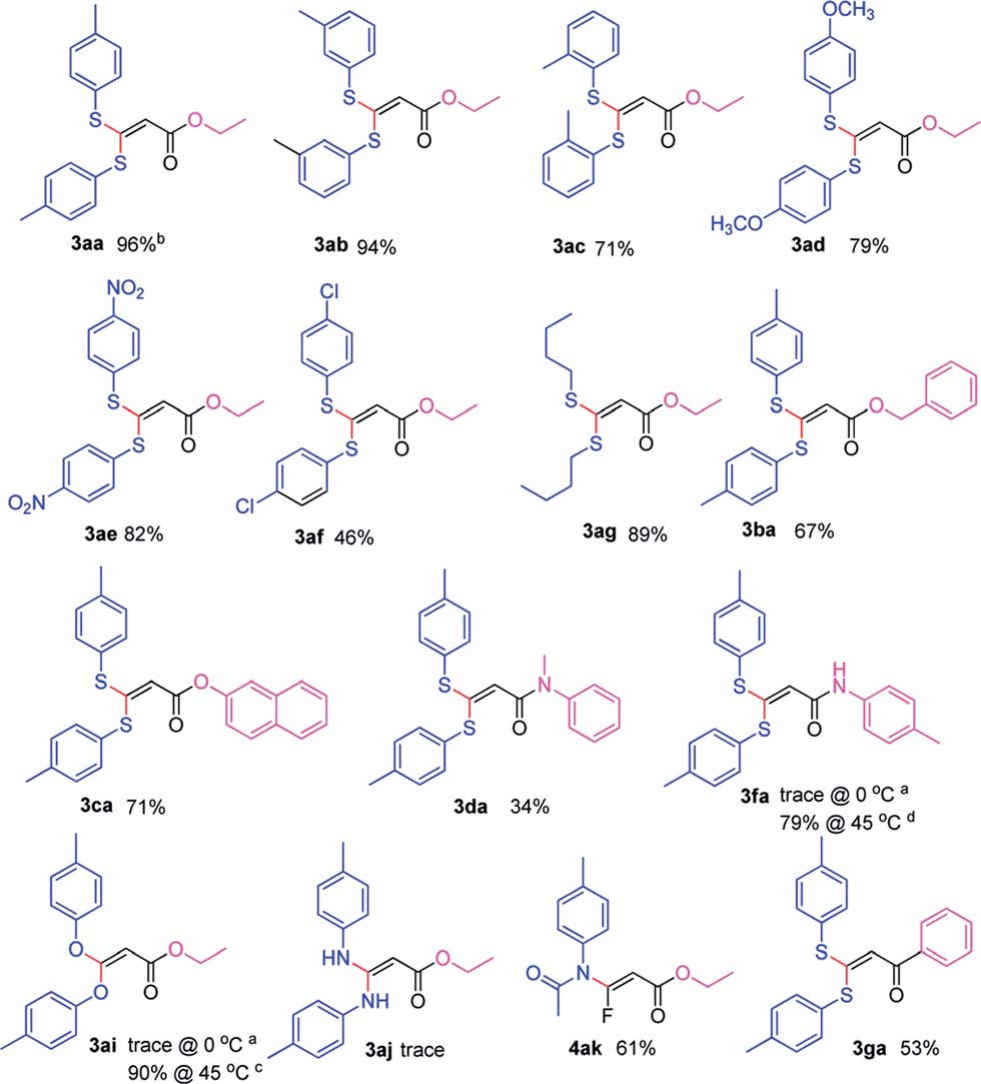

a Reaction conditions: a solution of 1 (1.0 mmol), 2 (2.0 mmol) and Cs_2_CO_3_ (2.0 mmol) in anhydrous THF (10 mL) was stirred at 0 °C for 2 h at under Ar.

b The isolated yield based on 1.

c The reaction was run at 45 °C.

d The reaction was run at 45 °C in anhydrous DMSO.

On the other hand, for the different 3,3,3-trifluoropropanoyl esters, the reaction also gave the similar results in the case of benzyl ester and β-naphthalenyl ester (3ba, 43%; 3ca, 71%). *N*,*N*-substituted 3,3,3-trifluoropropanamide was able to carry out this transformation with a lower yield (3da, 34%). In contrast, the *N*-monosubstituted amide only produced a trace amount of the desired product at 0 °C, but could afford the product at 45 °C in DMSO (3fa, 79%).

Furthermore, the *O*- and *N*-containing nucleophiles were tested in the reaction, for example, *p*-cresol successfully afforded the defluorinated product at a higher temperature (3ai, 90% at 45 °C). However, in terms of the *N*-containing nucleophile, *p*-toluidine did not afford the desired compound (3aj, trace) under Cs_2_CO_3_-, NaHMDS-, or *t*-BuOK-promoted conditions, possibly due to poor nucleophilicity of the amine or instability of 3aj. In contrast, the amide was able to produce the partial-defluorinated product (4ak, 61%) with *Z*-configuration. To our surprise, α-CF_3_ ketone was also proved flexible in the reaction, for example, 3,3,3-trifluoro-1-phenylpropan-1-one could react with 2a under the standard condition, and then gave 3ga in 53% yield.

Next, this defluorinative functionalization was switched from those mono-dentate nucleophiles to the bidentate nucleophiles, the results were depicted in Table 3. When one equivalent of catechol was used as one *O*,*O*-bidentate nucleophile, the reaction did not occur at 0 °C, but at a higher temperature (45 °C) the cyclized compound was smoothly produced (5am, 83%). Similarly, 2-mercaptophenol, one *O*,*S*-bidentate nucleophile, could smoothly afford the corresponding product (5an, 66%), and interestingly, the single crystal X-ray diffraction experiment demonstrated that the stereo configuration between H atom at the olefin and O atom at the heterocycle was *syn*-configuration,^27^ indicating there evidently existed bulkier repulse between H atom and S atom.

**Table 3:** Defluorination and functionization of ethyl 3,3,3-trifluoropropanoylate with some bidentate nucleophiles^a^

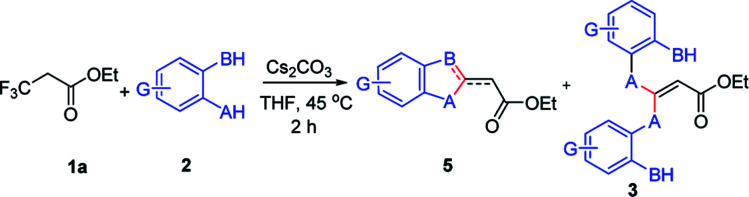
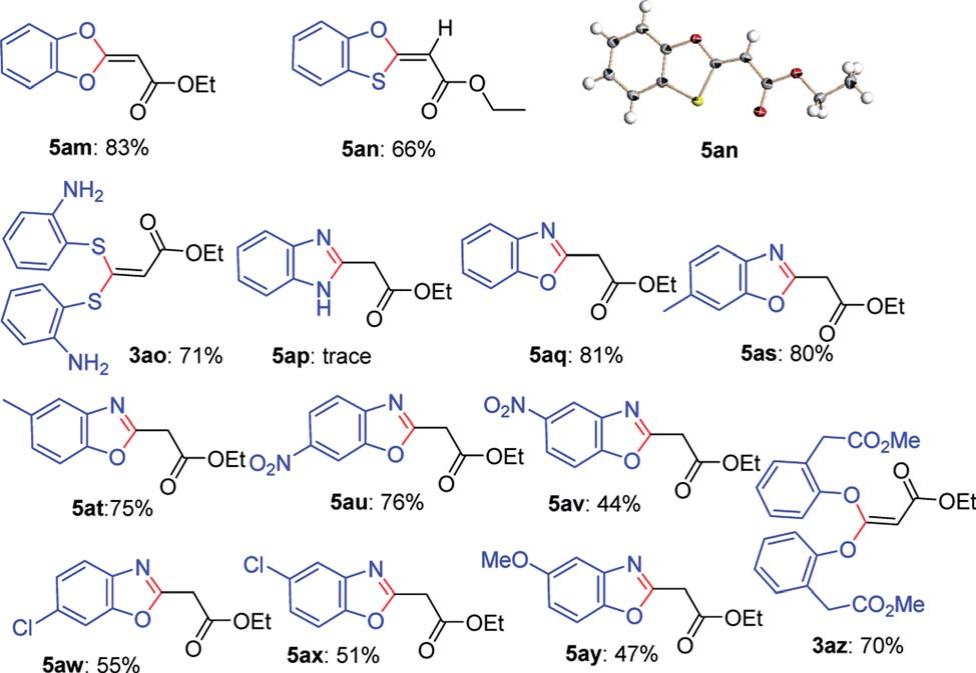

a Reaction conditions: a solution of 1a (1.0 mmol), 2 (1.0 mmol) and Cs_2_CO_3_ (2.0 mmol)in anhydrous THF (10 mL) was stirred at 45 °C for 2 h under Ar. The isolated yield based on 1a.

Unexpectedly, 2-aminothiophenol, as one *N*,*S*-bidentate nucleophile, did not give the desired product, instead, it resulted in the undesired product (3ao, 71%), possibly due to the weaker nucleophilicity of amine than that of thiol. Furthermore, the reaction using benzene-1,2-diamine as one *N*,*N*-bidentate nucleophile led to complicated products (5ap, trace).

Interestingly, 2-aminophenol, as one *N*,*O*-bidentate nucleophile, produced the benzooxazole derivative at 45 °C in a good yield (5aq, 81%) *via* defluorinative substitution, intramolecularly defluorinative substitution and aromatization. To our delight, a set of 2-aminophenols substituted at the aromatic ring could easily produce the corresponding ethyl 2-(benzo[*d*]oxazol-2-yl)acetates in moderate to good yields (5aq–5ay, 44–81%). However, when one *C*,*O*-bidentate nucleophile, such as methyl 2-(2-hydroxyphenyl)acetate, was used in the reaction, only O atom instead of C atom acted as the nucleophile (3az, 70%).

**Scheme 1:**
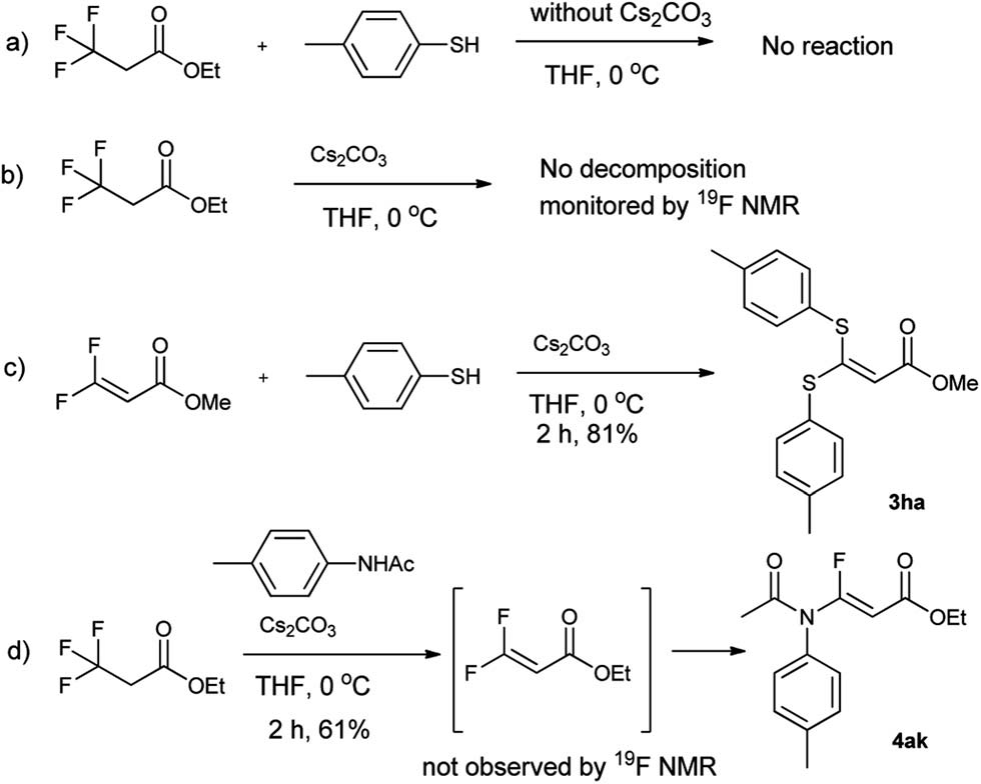
The control experiments.

To understand the reaction mechanism, the control experiments were performed, as shown in Scheme 1. If the reaction was run in absence of Cs_2_CO_3_, the reaction did not occur (Scheme 1a). Meanwhile the trifluoropropanoylate ester was not decomposed by Cs_2_CO_3_ in the absence of nucleophile (Scheme 1b), although decomposition occurred with a strong base (such as NaHMDS). On the other hand, if methyl 3,3-difluoroacrylate was subjected to the same basic condition, the desired compound (3ha) could be produced (Scheme 1c), strongly indicating the 3,3-difluoroacrylate was the intermediate. Although we attempted to isolate the corresponding 3,3-difluoroacrylamide or monitor other fluoro organic compounds by ^19^F NMR in the case of 4ka, unfortunately we did not succeed (Scheme 1d).

**Scheme 2:**
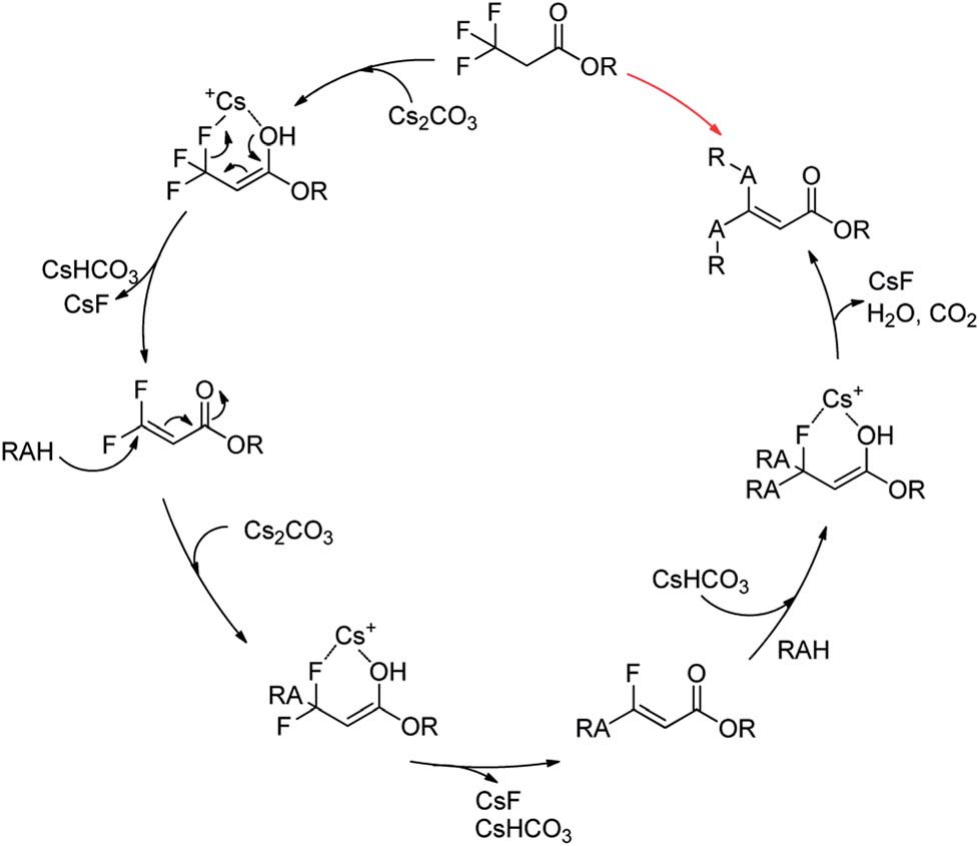
A plausible mechanism of the defluorination and functionization of ethyl 3,3,3-trifluoropropanoylate.

Together with the above results, we proposed a plausible mechanism (Scheme 2).^22–24^ At first, Cs_2_CO_3_ promoted the dehydrofluorinative elimination of trifluoropropanoylate ester in the presence of one suitable nucleophile, and the resultant 3,3-difluoroacrylate ester was rapidly added by the nucleophile, then the adduct continued to rapidly undergo elimination to afford α,β-unsaturated monofluorinated carbonyl compound, and in general, the further addition and dehydrofluorinative elimination continued to occur and finally resulted in the full-defluorinated product.

## Conclusions

In conclusion, we have developed an efficient defluorinated and functionized method of 3,3,3-trifluoropropanoic acid derivatives, and in this method, a mild base (Cs_2_CO_3_) enables to promote defluorination and functionalization of the β,β,β-trifluoro carbonyl compounds in the presence of a series of *N*-, *O*-, *S*-nucleophiles. By the current method, the α-trifluoromethyl carbonyl compounds easily afford β,β-*N*-, *O*-, and/or *S*-substituted α,β-unsaturated products in moderate to excellent yields, and α-trifluoromethyl ester can also be transformed into the corresponding benzooxazoles in good yields when 2-aminophenols were used as nucleophiles.

## Conflicts of interest

There are no conflicts to declare.

## Supplementary Material

RA-008-C8RA02353K-s001

RA-008-C8RA02353K-s002
