# Supplementary material for: Cs2CO3-promoted defluorination and functionalization of α-CF3 carbonyl compounds in the presence of N-, O-, and/or S-nucleophiles
Source: RSC Adv. 2018 Apr 30;8(29):16019–23. doi: 10.1039/c8ra02353k (PMC9080061; doi:10.1039/c8ra02353k)
Supplement: RA-008-C8RA02353K-s001 [file RA-008-C8RA02353K-s001.pdf]

Electronic Supplementary Material (ESI) for RSC Advances.
This journal is © The Royal Society of Chemistry 2018

Cs_2CO_3-promoted defluorination and functionalization of α-CF_3 carbonyl compounds in the presence of N-, O-, and/or S-nucleophiles

Yue Wu,[a] Bingbing Zhang,[a] Yinying Zheng,[a] Yuheng Wang[a] and Xinsheng Lei*,[a]

[a.] School of Pharmacy, Fudan University, 826 Zhangheng Road, Pudong Zone, Shanghai 201203, China.

Email: leixs@fudan.edu.cn

Table of Contents:

1. **General Experimental ·· S-2**
2. **Experimental Procedures ·· S-3**
3. **Spectral data of the new compounds ································ S-4**
4. **Copies of ^{1}H NMR and ^{13}C NMR spectra of the compounds ··········· S-15**

General Experimental:

The chemicals and reagents were purchased from Acros, Alfa Aesar, and National Chemical Reagent Group Co. Ltd., P. R. China, and used without further purification. Anhydrous solvents (THF, MeOH, DMF, DCM, and CH_3CN) used in the reactions were dried and freshly distilled before use. Petroleum ether (PE) used had a boiling range of 60−90 °C. All the reactions were carried out under Ar atmosphere, otherwise stated else. Oxygen and/or moisture sensitive solids and liquids were transferred appropriately. Concentration of solutions in *vacuo* was accomplished using a rotary evaporator fitted with a water aspirator. Residual solvents were removed under high vacuum (0.1-0.2 mm Hg). The progress of the reactions was monitored by TLC (silica-coated glass plates) and visualized under UV light, and by using iodine, ceric ammonium molybdate stain or phosphomolybdic acid. Melting points were measured on a SGW X-4 microscopy melting point apparatus without correction. ^{1}H NMR and ^{13}C NMR spectra were recorded either on a 400 MHz Varian Instrument at 25 °C or 600 MHz Bruke Instrument at 25 °C, using TMS as an internal standard, respectively. Multiplicity is tabulated as s for singlet, d for doublet, dd for doublet of doublet, t for triplet, and m for multiplet. Coupling constants (J) are reported in Hertz. ^{13}C NMR spectra were completely hetero-decoupled and measured at 150 MHz. HRMS spectra were recorded on Finnigan- Mat-95 mass spectrometer, equipped with ESI source. Single crystal X-ray diffraction measurements were performed with a diffractometer working with graphite-monochromated Cu Kα radiation.

Experimental Procedures:

The preparation of compound 3 or 4 in Table 2:

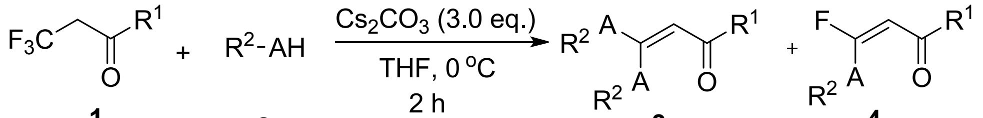

1 2 3 4

Method a: To a solution of 3,3,3-trifluoropropanoic acid derivatives (1.0 mmol) in THF (5 mL) was added the mono-dentate S-, O-, and N-nucleophiles (2.0 mmol) and Cs_2CO_3 (2.0 mmol)at 0 °C and then stirred for 2 h at under Ar. The reaction was ended with a saturated aqueous ammonium chloride solution (10 mL) and extracted with CH_2Cl_2 (DCM, 20 mL*3). After workup, the product was purified by flash chromatography (Petroleum Ether/Ethyl Acetate, PE/EA).

Method c: To a solution of ethyl 3,3,3-trifluoropropanoate (1.0 mmol) in THF (5 mL), was added the mono-dentate S-, O-, and N-nucleophiles (2.0 mmol) and Cs_2CO_3 (2.0 mmol) at 45 °C for 2 h at under Ar. The reaction was ended with a saturated aqueous ammonium chloride solution (10 mL) and extracted with DCM (20 mL*3). After workup, the product was purified by flash chromatography (PE /EA).

Method d: To a solution of ethyl 3,3,3-trifluoropropanoate (1.0 mmol) in anhydrous DMSO (5 mL), was added the mono-dentate S-, O-, and N-nucleophiles (2.0 mmol) and Cs_2CO_3 (2.0 mmol)at 45 °C for 2 h at under Ar. The reaction was ended with a saturated aqueous ammonium chloride solution (10 mL) and extracted with DCM (20 mL*3). After workup, the product was purified by flash chromatography (PE /EA).

The preparation of the compounds in Table 3:

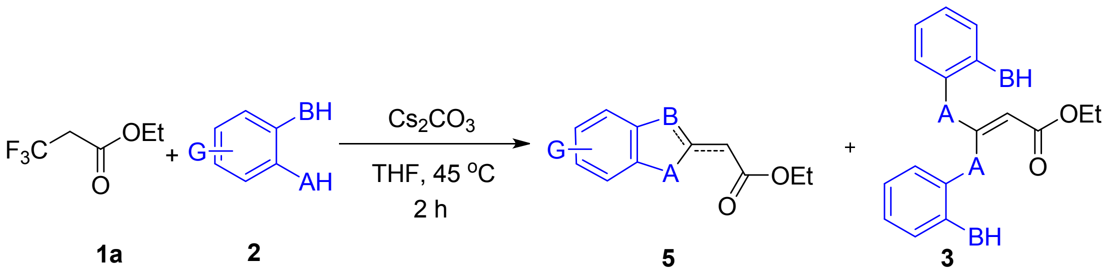

To a solution of ethyl 3,3,3-trifluoropropanoate (1.0 mmol) in THF (5 mL), was added the bidentate nucleophiles (1.0 mmol) and Cs_2CO_3 (2.0 mmol) at 45 °C for 2 h at under Ar. The reaction was ended with a saturated aqueous ammonium chloride solution (10 mL) and extracted with DCM (20 mL*3). After workup, the product was purified by flash chromatography (PE /EA).

Spectral data of all compounds:

Ethyl 3,3-bis(p-tolylthio)acrylate ***(3aa)***.

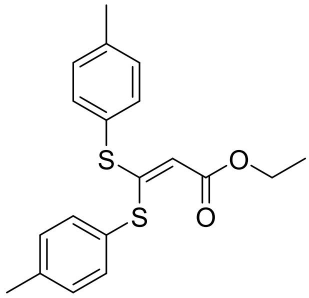

3aa

The resultant residue was purified by flash column chromatography (PE/EA=80/1) as a white solid. R_f= 0.20 (PE/EA = 80/1), **Mp** 85-86 ^{o}C.
^{1}H NMR (400 MHz, $CDCl_3$) δ 7.39 (d, *J* = 7.7 Hz, 2H), 7.09 – 6.88 (m, 6H), 5.03 (s, 1H), 3.92 (q, *J* = 7.1 Hz, 2H), 2.18 (s, 3H), 2.12 (s, 3H), 1.04 – 0.97 (m, 3H).
^{13}C NMR (150 MHz, $CDCl_3$) δ 165.4, 163.0, 140.5, 140.4, 137.0, 135.4, 130.7, 129.6, 126.7, 125.5, 107.1, 59.8, 21.5, 21.4, 14.4.
HRMS-ESI (*m/z*): $[M+H]^+$ calcd for $C_{19}H_{21}O_2S_2$ 345.0978; found 345.0973.

Ethyl 3,3-bis(m-tolylthio)acrylate ***(3ab).***

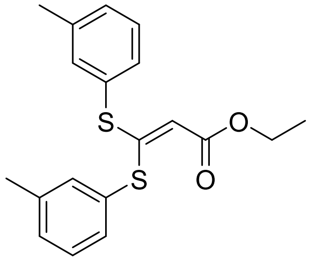

3ab

The resultant residue was purified by flash column chromatography (PE/EA=100/1) as a pale yellow liquid. R_f= 0.20 (PE/EA = 100/1).
^{1}H NMR (400 MHz, $CDCl_3$) δ 7.36 (d, *J* = 7.5 Hz, 2H), 7.23 – 6.98 (m, 6H), 5.21 (d, *J* = 1.4 Hz, 1H), 4.03 (qd, *J* = 7.1, 1.4 Hz, 2H), 2.26 (s, 3H), 2.21 (s, 3H), 1.12 (td, *J* = 7.1, 1.4 Hz, 4H).
^{13}C NMR (150 MHz, $CDCl_3$) δ 165.5, 161.9, 139.8, 138.7, 137.5, 135.8, 134.1, 132.4, 130.9, 130.9, 130.2, 129.7, 129.0, 128.7, 108.4, 60.1, 21.4, 21.3, 14.5.
HRMS-ESI (*m/z*): $[M + H]^+$ calcd for $C_{19}H_{21}O_2S_2$ 345.0978; found 345.0969.

Ethyl 3,3-bis(o-tolylthio)acrylate ***(3ac)***

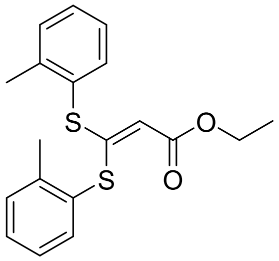

3ac

The resultant residue was purified by flash column chromatography (PE/EA=60/1) as a white solid. R_f= 0.20 (PE/EA = 60/1), **Mp** 84-85 ^oC.

^{1}H NMR (400 MHz, $CDCl_3$) δ 7.68 (d, *J* = 7.7 Hz, 1H), 7.47 – 7.08 (m, 7H), 5.11 (s, 1H), 4.13 (q, *J* = 7.1 Hz, 2H), 2.59 (s, 3H), 2.32 (s, 3H), 1.23 (t *J* = 7.1 Hz, 3H).

^{13}C NMR (150 MHz, $CDCl_3$) δ 165.7, 160.9, 144.6, 143.0, 138.2, 136.7, 131.4, 130.9, 130.9, 130.6, 129.4, 128.8, 127.4, 126.6, 106.6, 60.0, 21.4, 20.4, 14.5.

HRMS-ESI (*m/z*): $[M + H]^+$ calcd for $C_{19}H_{21}O_2S_2$ 345.0978; found 345.0974.

*Ethyl 3,3-bis((4-methoxyphenyl)thio)acrylate **(3ad)***

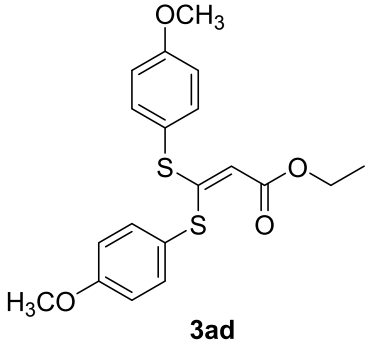

3ad

The resultant residue was purified by flash column chromatography (PE/EA=20/1) as a pale yellow liquid. R_f= 0.21 (PE/EA = 20/1).

^{1}H NMR (400 MHz, $CDCl_3$) ^{1}H NMR (400 MHz, Chloroform-*d*) δ 7.69 – 7.57 (m, 2H), 7.30 (d, *J* = 8.7 Hz, 2H), 6.99 – 6.84 (m, 4H), 5.14 (s, 1H), 4.13 (q, *J* = 7.1 Hz, 2H), 3.83 (d, *J* = 9.8 Hz, 6H), 1.23 (t, *J* = 7.1 Hz, 3H).

^{13}C NMR (150 MHz, $CDCl_3$) δ 165.7, 164.7, 161.5, 161.2, 138.9, 137.4, 120.8, 119.7, 115.6, 114.5, 106.5, 60.0, 55.5, 14.6.

HRMS-ESI (*m/z*): $[M + H]^+$ calcd for $C_{19}H_{21}O_4S_2$ 377.0876; found 377.0868.

*Ethyl 3,3-bis((4-nitrophenyl)thio)acrylate **(3ae)***

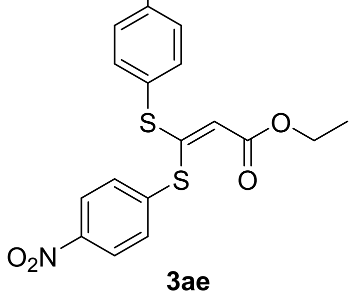

3ae

The resultant residue was purified by flash column chromatography (PE/EA=80/1) as a white foamy solid. R_f= 0.20 (PE/EA = 80/1).
^{1}H NMR (400 MHz, $CDCl_3$) δ 8.25 – 8.08 (m, 4H), 7.65 – 7.55 (m, 2H), 7.40 (dd, *J* = 8.9, 2.1 Hz, 2H), 6.10 (s, 1H), 4.23 (q, *J* = 7.1 Hz, 6H), 1.30 (t, *J* = 7.1 Hz, 5H).
^{13}C NMR (150 MHz, $CDCl_3$) δ 164.4, 151.4, 148.3, 147.9, 139.9, 138.8, 135.6, 132.8, 127.0, 124.5, 124.2, 123.6, 119.7, 61.1, 14.4.
HRMS-ESI (*m/z*): $[M + H]^+$ calcd for $C_{17}H_{15}N_2O_6S_2$ 407.0372; found 407.0378.

Ethyl 3,3-bis((4-chlorophenyl)thio)acrylate ***(3af)***

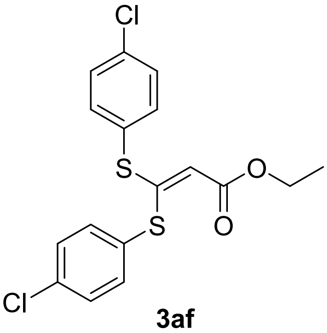

3af

The resultant residue was purified by flash column chromatography (PE/EA=100/1) as a pale yellow liquid. R_f= 0.20 (PE/EA = 100/1).
^{1}H NMR (400 MHz, $CDCl_3$) δ 7.69 – 7.44 (d, *J* = 8.0 Hz, 2H), 7.41 – 7.31 (m, 4H), 7.31 – 7.21 (d, *J* = 8.0 Hz, 2H), 5.37 (s, 1H), 4.15 (q, *J* = 7.2 Hz, 2H), 1.24 (t, *J* = 7.2 Hz, 3H).
^{13}C NMR (150 MHz, $CDCl_3$) δ 165.2, 159.6, 138.0, 136.8, 136.6, 136.3, 130.2, 129.2, 128.8, 127.7, 110.1, 60.3, 14.5.
HRMS-ESI (*m/z*): $[M + H]^+$ calcd for $C_{17}H_{15}Cl_2O_2S_2$ 384.9885; found 384.9887.

Ethyl 3,3-bis(butylthio)acrylate ***(3ag)***

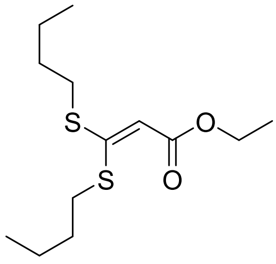

3ag

The resultant residue was purified by flash column chromatography (PE/EA=100/1) as a clear liquid. R_f= 0.21 (PE/EA = 100/1).
^{1}H NMR (400 MHz, $CDCl_3$) δ 5.63 (s, 1H), 4.17 (q, *J* = 7.2 Hz, 2H), 3.02 (t, *J* = 6.8 Hz, 2H), 2.87 (t, *J* = 6.8 Hz, 2H), 1.79 – 1.58 (m, 4H), 1.47 (m, 4H), 1.28 (tt, *J* = 7.6, 1.7 Hz, 3H), 0.95 (qd, *J* = 7.6, 3.6 Hz, 6H).
^{13}C NMR (150 MHz, CDCl3) δ 165.3, 160.6, 106.3, 59.9, 33.8, 31.5, 31.4, 29.64, 22.3, 22.2, 14.7, 13.8, 13.8.
HRMS-ESI (*m/z*): $[M + H]^+$ calcd for $C_{13}H_{25}O_2S_2$ 277.1291; found 277.1286.

Benzyl 3,3-bis(p-tolylthio)acrylate ***(3ba)***

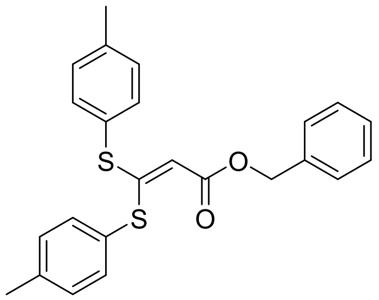

3ba

The resultant residue was purified by flash column chromatography (PE/EA=60/1) as a white solid. R_f= 0.25 (PE/EA = 60/1). **Mp** 123-124 °C.
^{1}H NMR (400 MHz, $CDCl_3$) δ 7.59 (dd, *J* = 8.1, 2.2 Hz, 2H), 7.40 – 7.06 (m, 11H), 5.24 (s, 1H), 5.11 (s, 2H), 2.40 (s, 3H), 2.34 (s, 3H).
^{13}C NMR (150 MHz, $CDCl_3$) δ 164.6, 163.5, 140.0, 139.9, 136.4, 135.7, 134.8, 130.1, 129.0, 127.9, 127.9, 127.5, 125.8, 124.8, 105.9, 65.1, 20.9, 20.7.
HRMS-ESI (*m/z*): $[M + H]^+$ calcd for $C_{24}H_{23}O_2S_2$ 407.1134; found 407.1130.

Naphthalen-2-yl 3,3-bis(p-tolylthio)acrylate ***(3ca)***

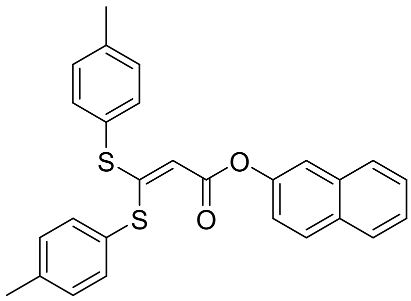

3ca

The resultant residue was purified by flash column chromatography (PE/EA=20/1) as a white solid. R_f= 0.20 (PE/EA = 20/1). **Mp** 121-122 °C.
^{1}H NMR (400 MHz, $CDCl_3$) δ 7.75 (dd, *J* = 22.1, 8.0 Hz, 2H), 7.52 – 7.45 (m, 2H), 7.41 (d, *J* = 5.5 Hz, 1H), 7.24 – 7.16 (m, 4H), 7.18 – 7.06 (m, 6H), 5.49 (s, 1H), 2.32 (s, 3H), 2.28 (s, 3H).
^{13}C NMR (150 MHz, $CDCl_3$) δ 167.6, 164.0, 148.7, 141.1, 141.0, 137.2, 135.8, 134.0, 131.5, 131.1, 123.0, 129.3, 127.9, 127.8, 126.6, 126.5, 125.6, 125.4, 121.7, 118.8, 105.5, 21.7, 21.6.
HRMS-ESI (*m/z*): $[M + H]^+$ calcd for $C_{27}H_{23}O_2S_2$ 443.1134; found 443.1142.

3,3,3-Trifluoro-N-methyl-N-(p-tolyl)propenamide ***(3da)***

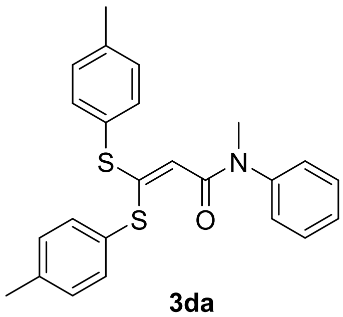

3da

The resultant residue was purified by flash column chromatography (PE/EA=15/1) as a white solid. R_f = 0.20 (PE/EA = 15/1). **Mp** 144-145 °C.

^{1}H NMR (400 MHz, $CDCl_3$) δ 7.50 (d, *J* = 7.7 Hz, 2H), 7.11 (d, *J* = 7.7 Hz, 2H), 6.89 (m, 4H), 6.81 (d, *J* = 8.0 Hz, 2H), 6.73 (d, *J* = 7.6 Hz, 2H), 4.98 (s, 1H), 3.15 (s, 3H), 2.31 (s, 3H), 2.27 (s, 3H), 2.20 (s, 3H).

^{13}C NMR (150 MHz, $CDCl_3$) δ 165.5, 157.4, 141.5, 140.2, 139.5, 137.0, 135.3, 130.1, 129.9, 129.6, 127.5, 127.0, 110.2, 36.9, 21.6, 21.5, 21.3.

HRMS-ESI (*m/z*): $[M + H]^+$ calcd for $C_{24}H_{24}NOS_2$ 406.1294; found 406.1319.

N-(p-tolyl)-3,3-bis(p-tolylthio)acrylamide ***(3fa).***

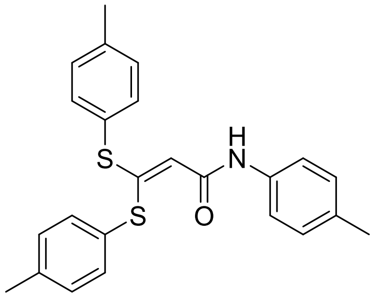

3fa

The resultant residue was purified by flash column chromatography (PE/EA=8/1) as a white solid. R_f = 0.21 (PE/EA = 8/1). **Mp** 116-117 °C.

^{1}H NMR (400 MHz, $CDCl_3$) δ 7.57 (d, *J* = 7.6 Hz, 2H), 7.39 (s, 2H), 7.25 (dd, *J* = 35.3, 7.6 Hz, 6H), 7.06 (d, *J* = 8.3 Hz, 2H), δ 5.30 (s, 1H), 2.39 (s, 3H), 2.38 (s, 3H), 2.28 (s, 3H).

^{13}C NMR (150 MHz, $CDCl_3$) δ 163.2, 158.0, 140.5, 136.4, 135.8, 135.6, 133.6, 130.8, 129.8, 129.6, 127.5, 119.7, 112.4, 21.6, 21.6, 21.1.

HRMS-ESI (*m/z*): $[M + H]^+$ calcd for $C_{24}H_{24}NOS_2$ 406.1294; found 406.1319.

Ethyl 3,3-bis(p-tolyloxy)acrylate ***(3ai)***

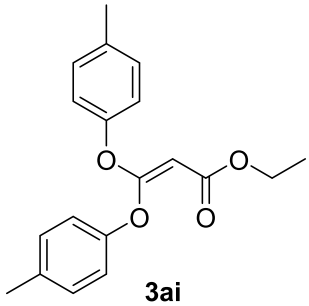

3ai

The resultant residue was purified by flash column chromatography (PE/EA=40/1) as a clear liquid. R_f= 0.20 (PE/EA = 40/1).
^{1}H NMR (400 MHz, $CDCl_3$) δ 7.21 – 7.03 (m, 6H), 6.93 (d, *J* = 8.3 Hz, 2H), 4.49 (s, 1H), 4.10 (q, *J* = 7.1 Hz, 2H), 2.33 – 2.32 (m, 6H), 1.20 (t, *J* = 7.1 Hz, 3H).
^{13}C NMR (150 MHz, $CDCl_3$) δ 166.4, 166.1, 151.9, 150.5, 136.1, 134.4, 130.7, 130.2, 120.6, 119.0, 80.8, 59.8, 21.1, 21.0, 14.6.
HRMS-ESI (*m/z*): $[M + H]^+$ calcd for $C_{19}H_{21}O_4$ 313.1434; found 313.1441.

Ethyl (Z)-3-fluoro-3-(N-(p-tolyl)acetamido)acrylate ***(4ak)***

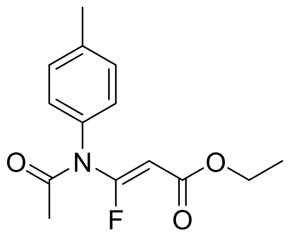

4ak

The resultant residue was purified by flash column chromatography (PE/EA=4/1) as a clear liquid. R_f= 0.25 (PE/EA = 4/1).
^{1}H NMR (400 MHz, CDCl) δ 7.26 (d, *J* = 8.2 Hz, 2H), 7.14 (d, *J* = 7.9 Hz, 2H), 5.40 (d, *J* = 27.1 Hz, 1H), 4.18 (q, *J* = 7.2 Hz, 2H), 2.39 (s, 4H), 2.13 (s, 3H), 1.27 (t, *J* = 7.2 Hz, 3H).
^{13}C NMR (150 MHz, $CDCl_3$) δ 170.2, 164.0, 159.1, 157.2, 139.5, 136.1, 130.8, 127.7, 94.7, 94.6, 60.7, 23.9, 23.8, 21.3, 14.4.
HRMS-ESI (*m/z*): $[M + Na]^+$ calcd for $C_{14}H_{16}FNNaO_3$ 288.1006; found 288.0987.

1-Phenyl-3,3-bis(p-tolylthio)prop-2-en-1-one ***(3ga)***

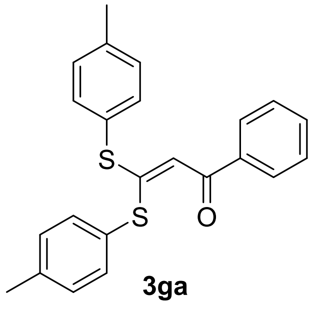

3ga

The resultant residue was purified by flash column chromatography (PE/EA=45/1) as a white solid. R_f= 0.20 (PE/EA = 45/1). **Mp** 141-142 °C.
^{1}H NMR (400 MHz, $CDCl_3$) δ 7.62 (td, *J* = 5.8, 4.0, 2.0 Hz, 4H), 7.50 – 7.38 (m, 1H), 7.39 – 7.29 (m, 4H), 7.29 – 7.19 (m, 4H), δ 6.41 (s, 1H), 2.41 (s, 3H), 2.40 (s, 3H).
^{13}C NMR (150 MHz, $CDCl_3$) δ 186.3, 167.8, 140.9, 140.8, 139.2, 136.9, 135.7, 131.9, 130.9, 129.9, 128.5, 128.0, 127.4, 126.4, 112.0, 21.7, 21.6.
HRMS-ESI (*m/z*): $[M + H]^+$ calcd for $C_{23}H_{21}OS_2$ 377.1028; found 377.1031.

Ethyl 2-(benzo[d][1,3]dioxol-2-ylidene)acetate ***(5am)***

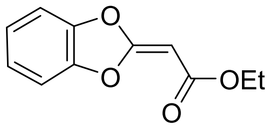

5am

The resultant residue was purified by flash column chromatography (PE /EA = 20/1) as a pale yellow solid. R_f = 0.20 (PE/EA = 20/1), **Mp** 84-85 ^{o}C.
^{1}H NMR (400 MHz, CDCl3) δ 7.35 – 7.22 (m, 1H), 7.16 – 7.15 (m, 3H), 5.01 (s, 1H), 4.22 (q, J = 7.2 Hz, 2H), 1.31 (t, J = 7.2 Hz, 3H).
^{13}C NMR (150 MHz, $CDCl_3$) δ 167.6, 166.4, 145.3, 143.8, 124.8, 124.6, 110.7, 109.9, 70.7, 59.9, 14.7.
HRMS-ESI (*m/z*): $[M + H]^+$ calcd for $C_{11}H_{11}O_4$ 207.0652; found 207.0645.

Ethyl (Z)-2-(benzo[d][1,3]oxathiol-2-ylidene)acetate ***(5an)***

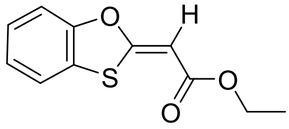

5an

The resultant residue was purified by flash column chromatography (PE/EA=75/1) as a white solid. R_f = 0.23 (PE/EA = 75/1), **Mp** 115-116 ^{o}C.
^{1}H NMR (400 MHz, $CDCl_3$) δ 7.49 – 7.37 (m, 1H), 7.30 – 7.10 (m, 3H), 5.87 (d, J = 1.3 Hz, 1H), 4.25 (q, J = 7.1 Hz, 2H), 1.43 – 1.21 (m, 3H).
^{13}C NMR (150 MHz, $CDCl_3$) δ 171.6, 168.5, 152.3, 127.0, 124.7, 124.6, 122.0, 111.3, 89.2, 60.3, 14.7.
HRMS-ESI (*m/z*): $[M + H]^+$ calcd for $C_{11}H_{11}O_3S$ 223.0423; found 223.0422.

Ethyl 3,3-bis((2-aminophenyl)thio)acrylate ***(3ao)***

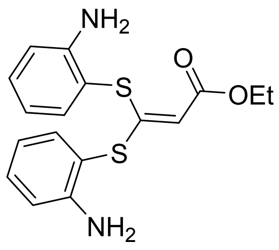

3ao

The resultant residue was purified by flash column chromatography (PE/EA=75/1) as a white solid. R_f = 0.23 (PE/EA = 75/1), **Mp** 115-116 ^{o}C.
^{1}H NMR (400 MHz, $CDCl_3$) δ 7.53 (d, J = 7.7 Hz, 1H), 7.28 (dd, J = 12.8, 5.5 Hz, 1H), 7.22 (d, J = 7.3 Hz, 2H), 6.79 (d, J = 7.7 Hz, 2H), 6.70 (q, J = 7.7 Hz, 2H), 5.35 (s, 1H), 4.43 (brs, 4H), 4.13 (q, J = 7.3 Hz, 2H). 1.24 (t, J = 7.1 Hz, 3H).
^{13}C NMR (150 MHz, $CDCl_3$) δ 165.8, 159.2, 150.4, 149.1, 138.5, 137.6, 132.6, 132.5, 119.0, 118.9, 115.8, 115.6, 112.5, 112.0, 107.7, 60.2, 14.6.
HRMS-ESI (*m/z*): $[M + Na]^+$ calcd for $C_{17}H_{18}N_2NaO_2S_2$ 369.0702; found 369.0700.

Ethyl 2-(benzo[d]oxazol-2-yl)acetate ***(5aq)***

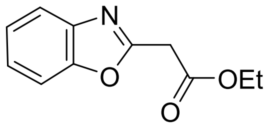

5aq

The resultant residue was purified by flash column chromatography (PE/EA=20/1) as a white solid. R_f= 0.20 (PE/EA = 20/1), **Mp** 56-57 °C.

^{1}H NMR (400 MHz, $CDCl_3$) δ 7.72 (d, *J* = 3.6 Hz, 1H), 7.71 (d, *J* = 3.6 Hz, 1H), 7.35– 7.10 (m, 2H), 4.25 (q, *J* = 7.1 Hz, 2H), 4.03 (s, 2H), 1.29 (t, *J* = 7.1 Hz, 3H).

^{13}C NMR (150 MHz, $CDCl_3$) δ 167.2, 159.7, 151.3, 141.3, 125.3, 124.6, 120.2, 110.8, 62.1, 35.5, 14.2.

HRMS-ESI (*m/z*): $[M + H]^+$ calcd for $C_{11}H_{12}NO_3$ 206.0812; found 206.0813.

Ethyl 2-(6-methylbenzo[d]oxazol-2-yl)acetate ***(5as)***

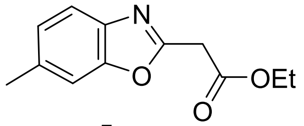

5as

The resultant residue was purified by flash column chromatography (PE/EA=18/1) as a yellow liquid. R_f= 0.20 (PE/EA = 18/1).

^{1}H NMR (400 MHz, $CDCl_3$) δ 7.57 – 7.46 (s, 1H), 7.46 – 7.37 (d, *J* = 8.4 Hz, 1H), 7.15 (d, *J* = 8.4 Hz, 1H), 4.40 – 4.13 (q, *J* = 7.2 Hz, 2H), 4.00 (s, 2H), 2.46 (s, 3H), 1.28 (t, *J* = 7.2, Hz, 3H).

^{13}C NMR (150 MHz, $CDCl_3$) δ 167.3, 159.8, 149.6, 141.5, 134.5, 126.4, 120.1, 110.2, 62.1, 35.6, 21.6, 14.3.

HRMS-ESI (*m/z*): $[M + H]^+$ calcd for $C_{12}H_{14}NO_3$ 220.0968; found 220.0967.

Ethyl 2-(5-methylbenzo[d]oxazol-2-yl)acetate ***(5at)***

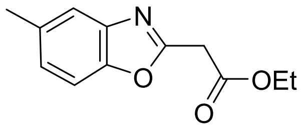

5at

The resultant residue was purified by flash column chromatography (PE/EA=18/1) as a pale yellow solid. R_f= 0.20 (PE/EA = 18/1), **Mp** 58-59 °C.

^{1}H NMR (400 MHz, $CDCl_3$) δ 7.49 (s, 1H), 7.39 (d, *J* = 8.4 Hz, 1H), 7.14 (d, *J* = 8.3 Hz, 1H), 4.24 (q, *J* = 7.1 Hz, 2H), 3.99 (s, 2H), 2.46 (s, 3H), 1.28 (t, *J* = 7.1 Hz, 3H).

^{13}C NMR (150 MHz, $CDCl_3$) δ 167.3, 159.2, 151.6, 139.1, 135.7, 125.8, 119.5, 110.9, 62.1, 35.5, 21.9, 14.3.

HRMS-ESI (*m/z*): $[M + H]^+$ calcd for $C_{12}H_{14}NO_3$ 220.0968; found 220.0969.

Ethyl 2-(6-nitrobenzo[d]oxazol-2-yl)acetate ***(5au)***

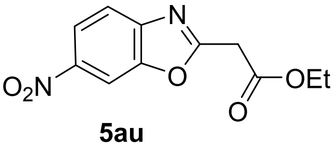

5au

The resultant residue was purified by flash column chromatography (PE/EA=13/1) as a pale yellow solid. R_f= 0.20 (PE/EA = 13/1), **Mp** 77-79 ^{o}C.

^{1}H NMR (400 MHz, $CDCl_3$) δ 8.46 (dt, J = 6.3, 2.4 Hz, 1H), 8.32 (dq, J = 8.9, 1.7 Hz, 1H), 7.83 (dd, J = 8.9, 1.7 Hz, 1H), δ 4.28 (q, J = 7.0 Hz, 2H), 4.10 (s, 2H), 1.31 (t, J = 7.0 Hz, 3H).

^{13}C NMR (150 MHz, $CDCl_3$) δ 166.4, 164.5, 150.4, 146.5, 145.6, 120.8, 120.4, 107.6, 62.5, 35.7, 14.3.

HRMS-ESI (*m/z*): $[M + H]^+$ calcd for $C_{11}H_{11}N_2O_5$ 251.0662; found 251.0665.

*Ethyl 2-(5-nitrobenzo[d]oxazol-2-yl)acetate **(5av)***

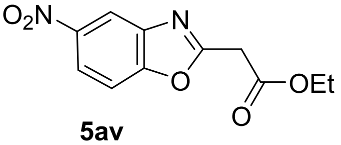

5av

The resultant residue was purified by flash column chromatography (PE/EA=20/1) as a pale yellow solid. R_f= 0.20 (PE/EA = 20/1), **Mp** 87-88 ^{o}C.

^{1}H NMR (400 MHz, $CDCl_3$) δ 8.46 (d, J = 2.1 Hz, 1H), 8.32 (d, J = 8.9 Hz, 1H), 7.91 – 7.78 (m, 1H), 4.27 (q, J = 7.0 Hz, 2H), 4.10 (s, 2H), 1.31 (t, J = 7.0 Hz, 3H).

^{13}C NMR (150 MHz, $CDCl_3$) δ 166.1, 159.6, 150.6, 139.3, 130.3, 124.6, 120.0, 110.7, 61.4, 34.6, 13.5.

HRMS-ESI (*m/z*): $[M + H]^+$ calcd for $C_{11}H_{10}N_2O_5$ 251.0662; found 251.0663.

*Ethyl 2-(6-chlorobenzo[d]oxazol-2-yl)acetate **(5aw)***

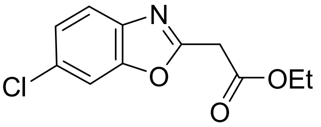

5aw

The resultant residue was purified by flash column chromatography (PE/EA=15/1) as a black solid. R_f= 0.21 (PE/EA = 15/1), **Mp** 85-86 ^{o}C.

^{1}H NMR (400 MHz, $CDCl_3$) δ 7.63 (dt, J = 8.5, 1.9 Hz, 1H), 7.55 (q, J = 1.9, 1.5 Hz, 1H), 7.33 (dt, J = 8.5, 1.5 Hz, 1H), δ 4.25 (q, J = 7.1 Hz, 2H), 4.01 (s, 2H), 1.29 (t, J = 7.1 Hz, 3H).

^{13}C NMR (150 MHz, $CDCl_3$) δ 166.1, 159.6, 150.6, 139.3, 130.3, 124.6, 120.0, 110.7, 61.4, 34.6, 13.5.

HRMS-ESI (*m/z*): $[M + H]^+$ calcd for $C_{11}H_{11}ClNO_3$ 240.0422; found 240.0419.

*Ethyl 2-(5-chlorobenzo[d]oxazol-2-yl)acetate **(5ax)***

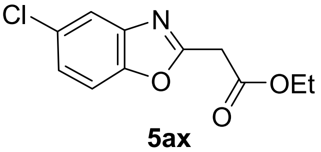

5ax

The resultant residue was purified by flash column chromatography (PE/EA=30/1) as a pale yellow solid. R_f= 0.23 (PE/EA = 30/1), **Mp** 85-86 °C.
^{1}H NMR (400 MHz, $CDCl_3$) δ 7.70 (q, J = 2.0 Hz, 1H), 7.45 (dt, J = 8.7, 1.8 Hz, 1H), 7.32 (dq, J = 8.7, 2.0 Hz, 1H), 4.25 (q, J = 7.1 Hz, 3H), 4.01 (s, 3H), 1.29 (t, J = 7.1 Hz, 5H).
^{13}C NMR (150 MHz, $CDCl_3$) δ 166.9, 161.2, 149.9, 142.4, 130.2, 125.7, 120.3, 111.6, 62.3, 35.5, 14.3.
HRMS-ESI (*m/z*): $[M + H]^+$ calcd for $C_{11}H_{11}ClNO_3$ 240.0422; found 240.0419.

Ethyl 2-(5-methoxybenzo[d]oxazol-2-yl)acetate ***(5ay)***

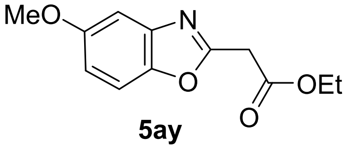

5ay

The resultant residue was purified by flash column chromatography (PE/EA=30/1) as a white liquid. R_f= 0.21 (PE/EA = 10/1).
^{1}H NMR (400 MHz, $CDCl_3$) δ 7.40 (dd, J = 8.9, 1.4 Hz, 1H), 7.19 (d, J = 1.4 Hz, 1H), 7.01 – 6.86 (m, 1H), 4.24 (q, J = 7.1 Hz, 2H), 3.99 (s, 2H), 3.85 (s, 3H), 1.28 (t, J = 7.1 Hz, 3H).
^{13}C NMR (150 MHz, $CDCl_3$) δ 167.2, 160.5, 157.4, 146.0, 142.2, 113.9, 110.9, 103.2, 62.1, 56.1, 35.6, 14.3.
HRMS-ESI (*m/z*): $[M + H]^+$ calcd for $C_{12}H_{14}NO_4$ 236.0917; found 236.0916.

Dimethyl 2,2'-(((3-ethoxy-3-oxoprop-1-ene-1,1-diyl)bis(oxy))bis(2,1-phenylene))diacetate ***(3az)***

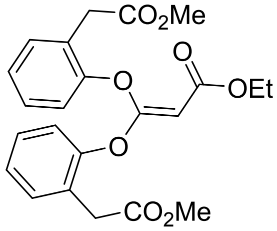

3az

The resultant residue was purified by flash column chromatography (PE/EA=5/1) as a clear liquid. R_f= 0.21 (PE/EA = 5/1).
^{1}H NMR (400 MHz, $CDCl_3$) δ 7.48 – 6.83 (m, 8H), δ 4.35 (s, 1H), 4.03 (q, J = 7.1 Hz, 2H), 3.72 (s, 2H), 3.60 (s, 3H), 3.53 (s, 3H), 3.44 (s, 2H), 1.12 (t, J = 7.1 Hz, 3H).
^{13}C NMR (150 MHz, $CDCl_3$) δ 171.5, 170.9, 165.7, 164.9, 151.8, 150.7, 132.0, 131.4, 129.3, 128.5, 126.7, 126.6, 126.2, 125.4, 121.2, 119.8, 79.9, 59.7, 52.2, 52.1, 35.4, 35.3, 14.5.
HRMS-ESI (*m/z*): $[M + H]^+$ calcd for $C_{23}H_{25}O_8$ 429.1544; found 429.1550.

Methyl 3,3-bis(p-tolylthio)acrylate ***(3ha)***

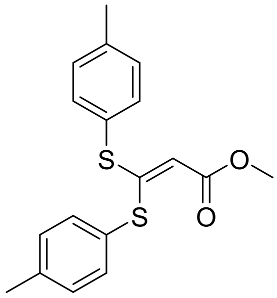

The resultant residue was purified by flash column chromatography (PE/EA=15/1) as a white foamy solid. R_f= 0.21 (PE/EA = 15/1). **Mp** 82-83 °C.

^{1}H NMR (400 MHz, $CDCl_3$) δ 7.59 (d, *J* = 8.1 Hz, 2H), 7.35 – 6.93 (m, 6H), 5.19 (s, 1H), 3.65 (s, 3H), 2.40 (s, 3H), 2.36 (s, 3H).

^{13}C NMR (150 MHz, $CDCl_3$) δ 166.0, 163.8, 140.8, 140.7, 137.2, 135.7, 130.9, 129.9, 126.9, 125.6, 106.8, 51.3, 21.7, 21.5.

HRMS-ESI (*m/z*): $[M + H]^+$ calcd for $C_{18}H_{19}O_2S_2$ 331.0821; found 331.0826.

Copies of ^{1}H NMR and ^{13}C NMR spectra of all compound:

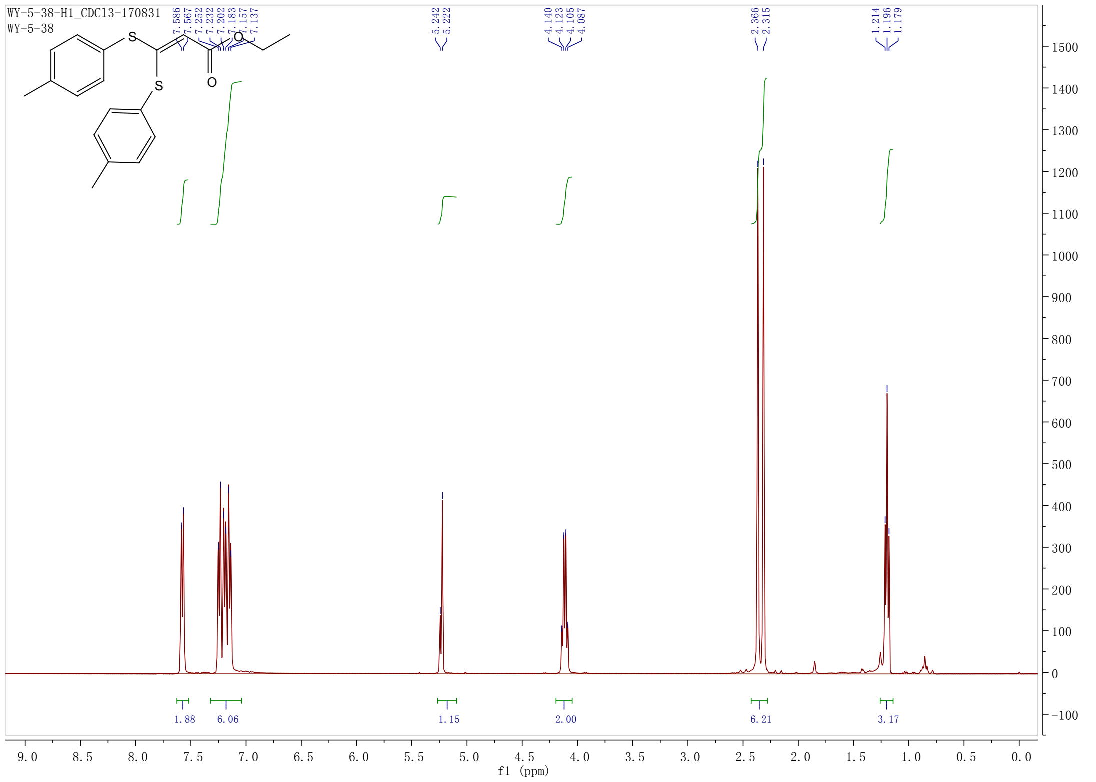

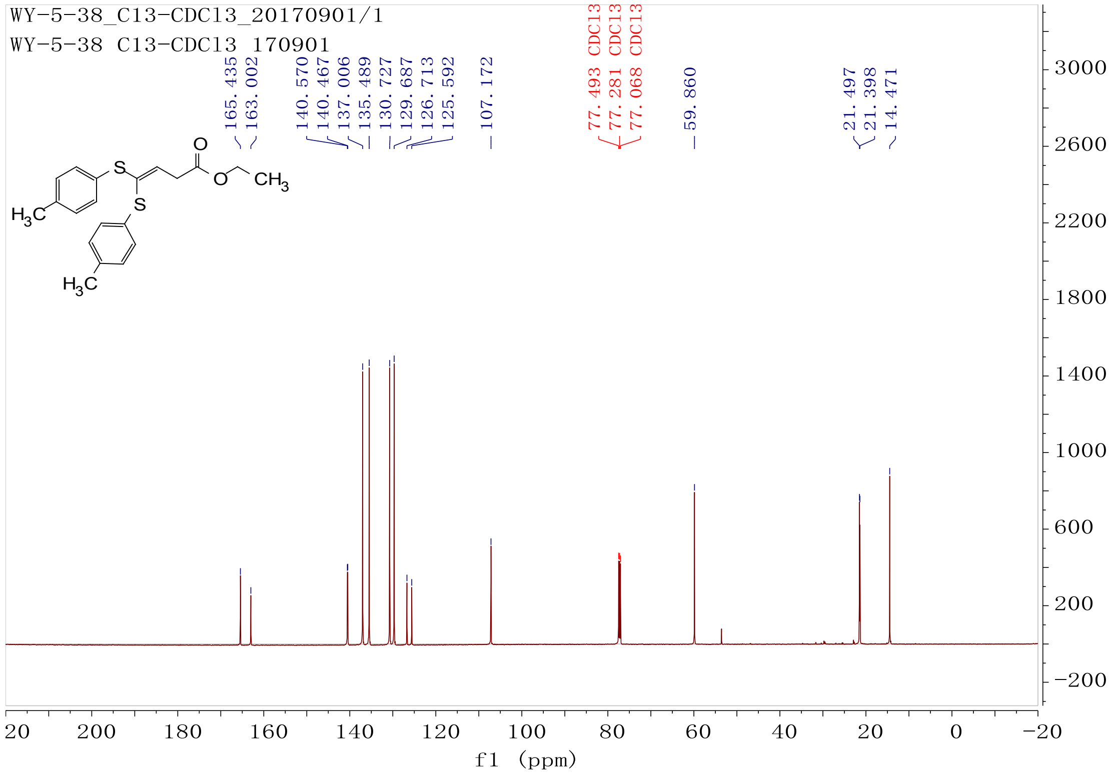

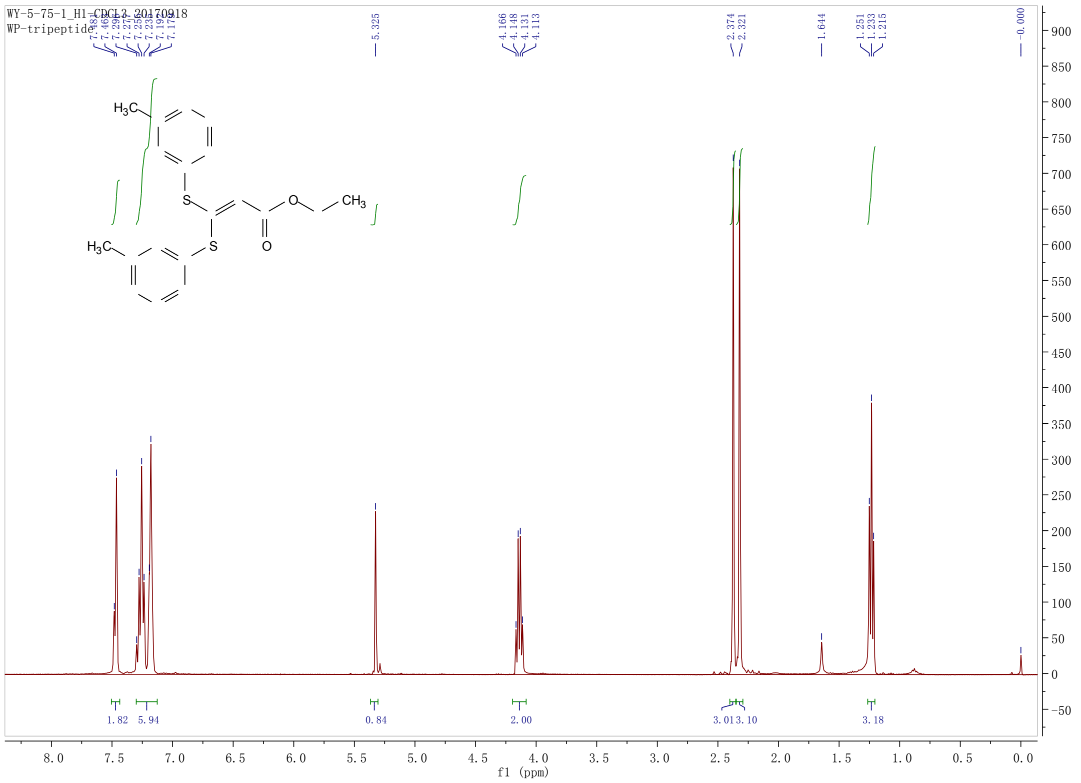
WY-5-75-1_H1-CDCL3-20170918
WP-tripeptide
5.325
4.166
4.148
4.131
4.113
2.374
2.321
1.644
1.251
1.233
1.215
0.000
1.82
5.94
0.84
2.00
3.01
3.10
3.18
f1 (ppm)

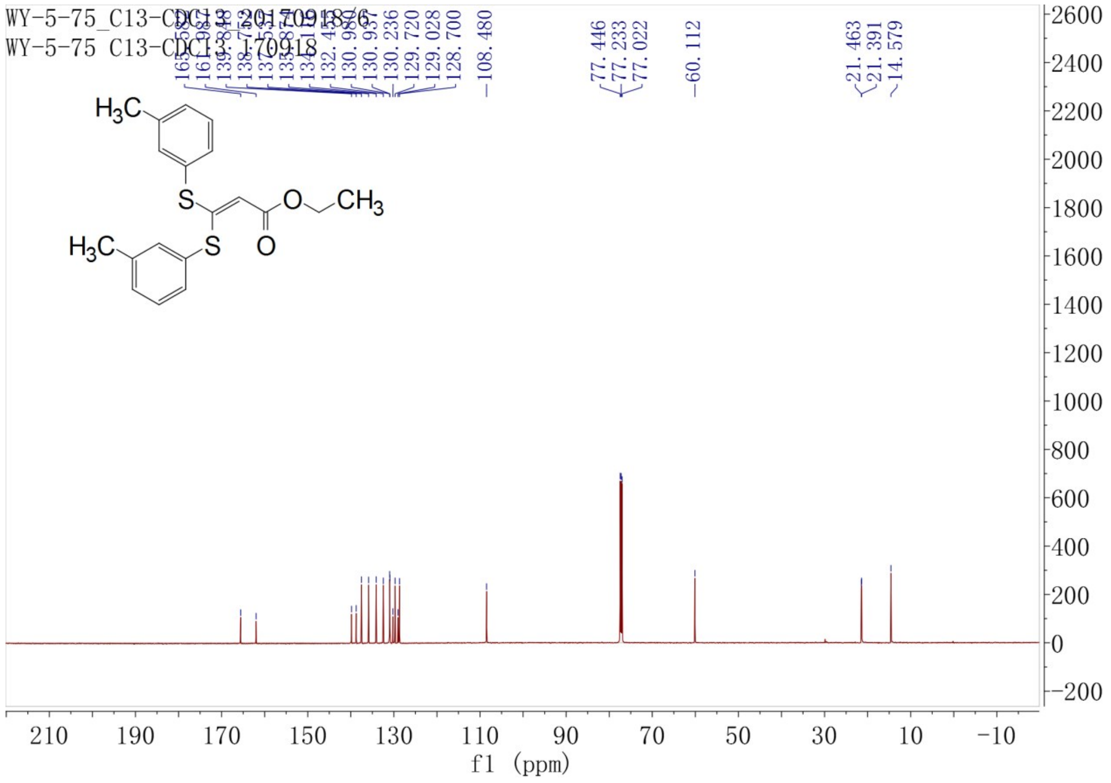
WY-5-75_C13-CDCl3-20170918
WY-5-75 C13-CDCl3 170918
132.450
130.980
130.937
130.236
129.720
129.028
128.700
108.480
77.446
77.233
77.022
60.112
21.463
21.391
14.579
f1 (ppm)

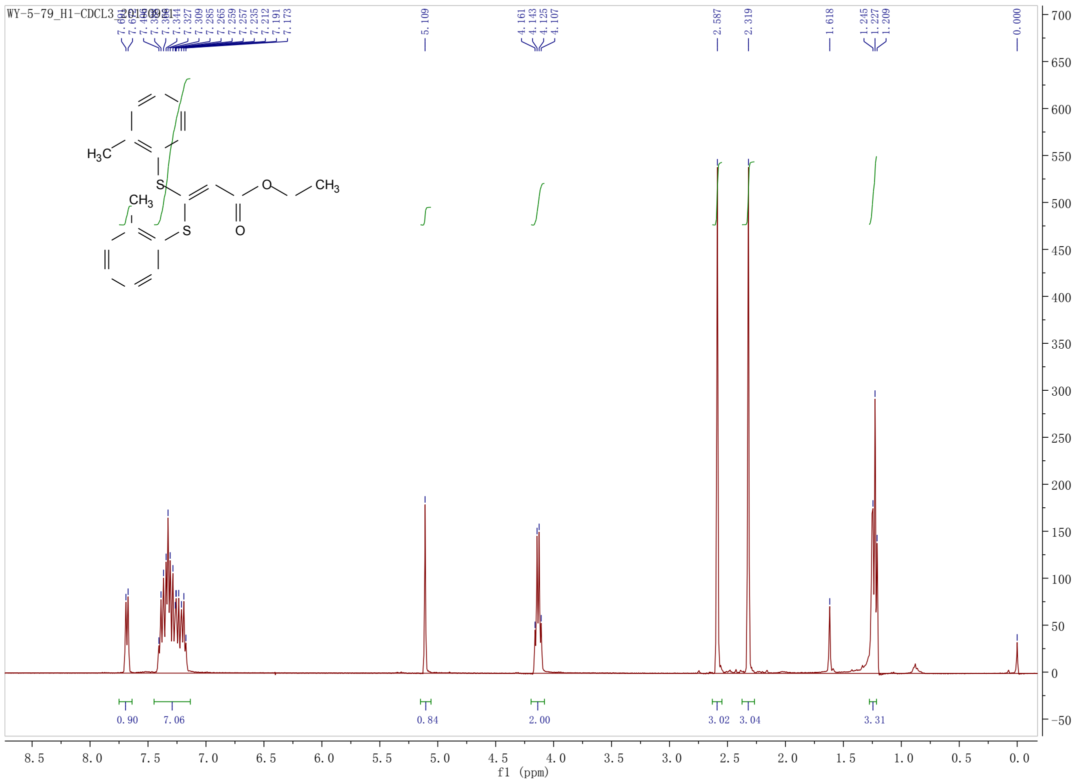
WY-5-79_H1-CDCL3_20170918
f1 (ppm)

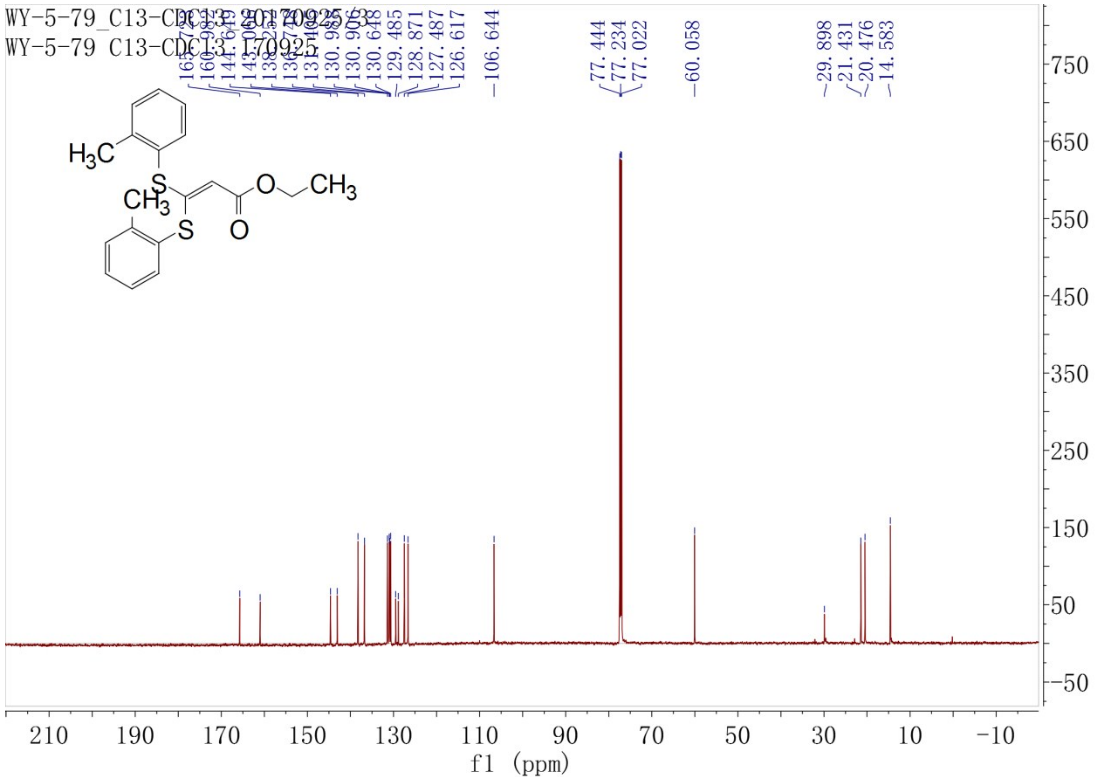
WY-5-79_C13-CDCl3_20170925
f1 (ppm)

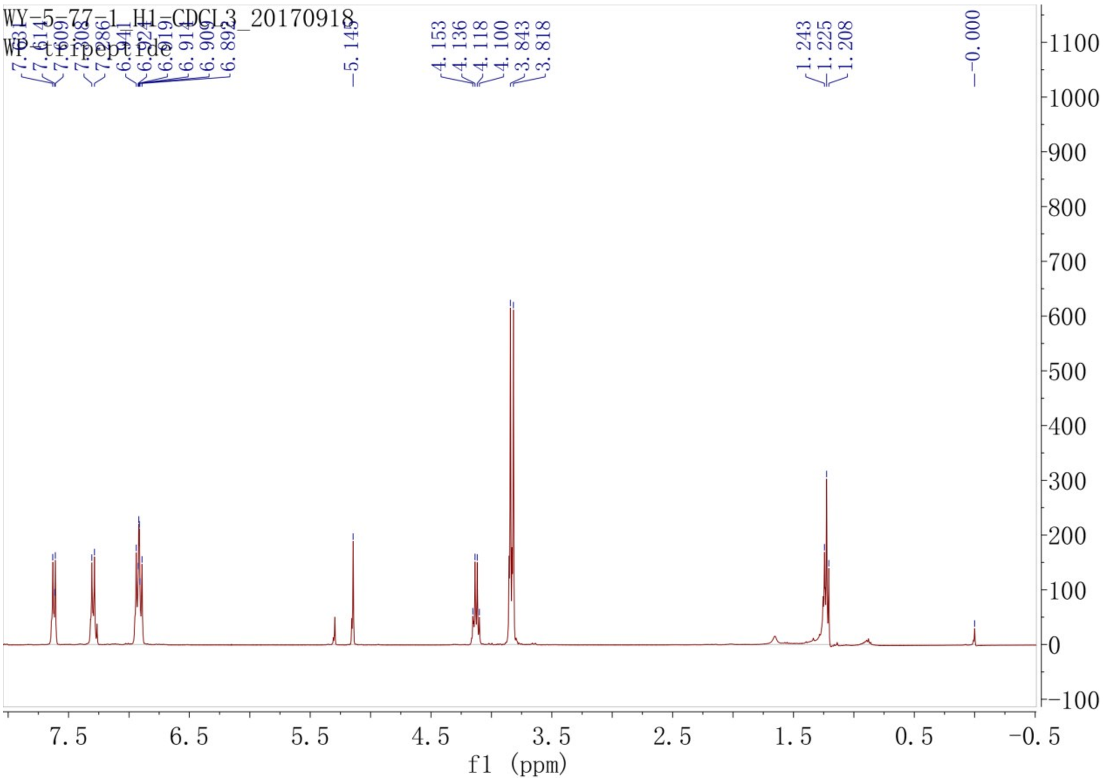
WY-5-77-1_H1-CDCl3_20170918
5.145
4.153
4.136
4.118
4.100
3.843
3.818
1.243
1.225
1.208
0.000
7.5
6.5
5.5
4.5
3.5
2.5
1.5
0.5
-0.5
f1 (ppm)

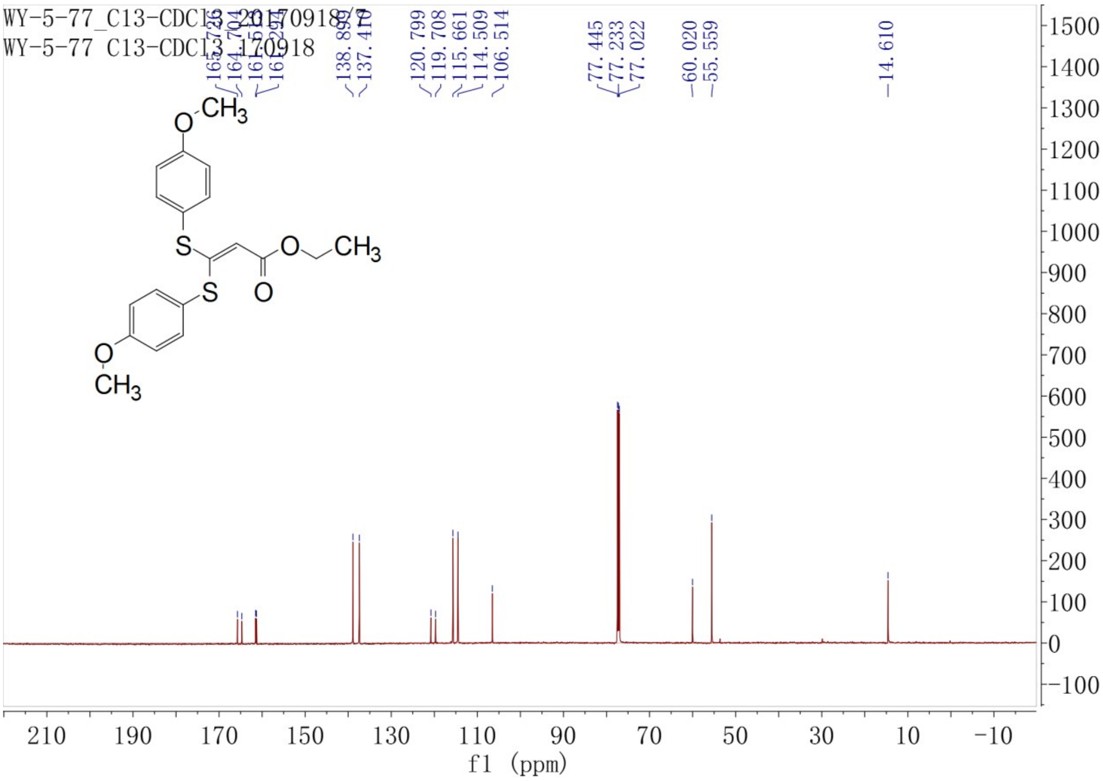
WY-5-77_C13-CDCl3_20170918
WY-5-77 C13-CDCl3_170918
138.899
137.410
120.799
119.708
115.661
114.509
106.514
77.445
77.233
77.022
60.020
55.559
14.610
CH3
O
S
S
O
CH3
O
CH3
210
190
170
150
130
110
90
70
50
30
10
-10
f1 (ppm)

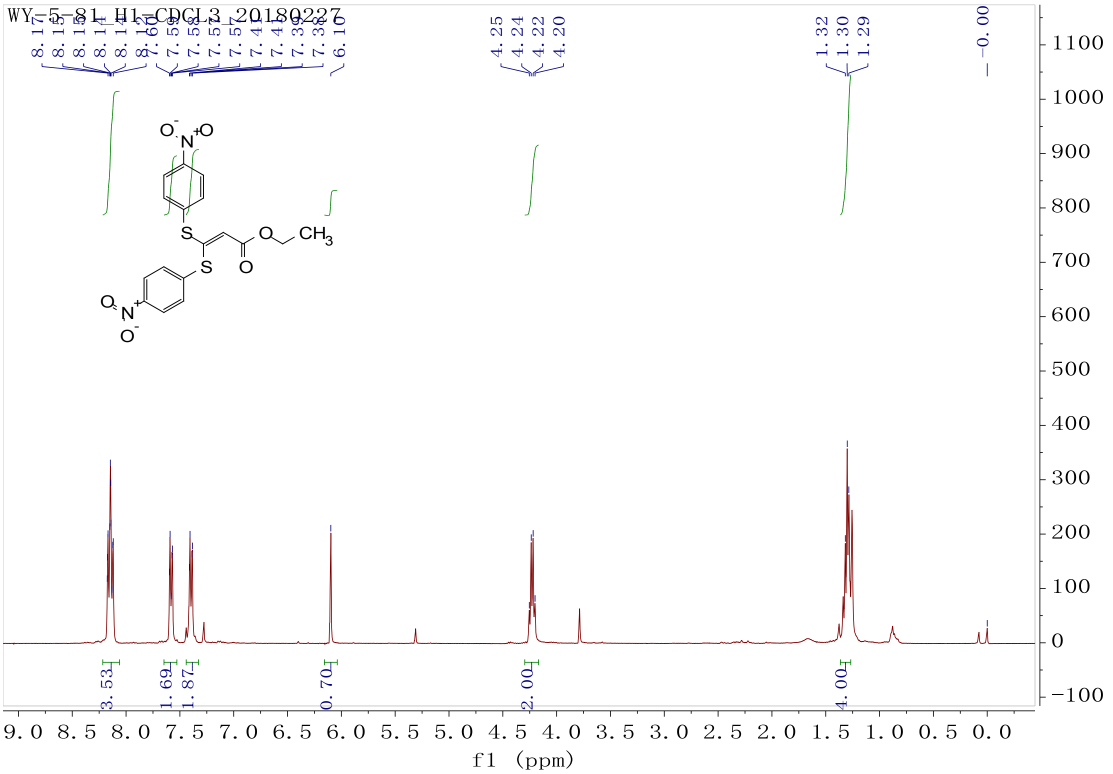
WY-5-81_H1-CDCl3_20180227
4.25
4.24
4.22
4.20
1.32
1.30
1.29
-0.00
6.10
3.53
1.69
1.87
0.70
2.00
4.00
CH3
f1 (ppm)

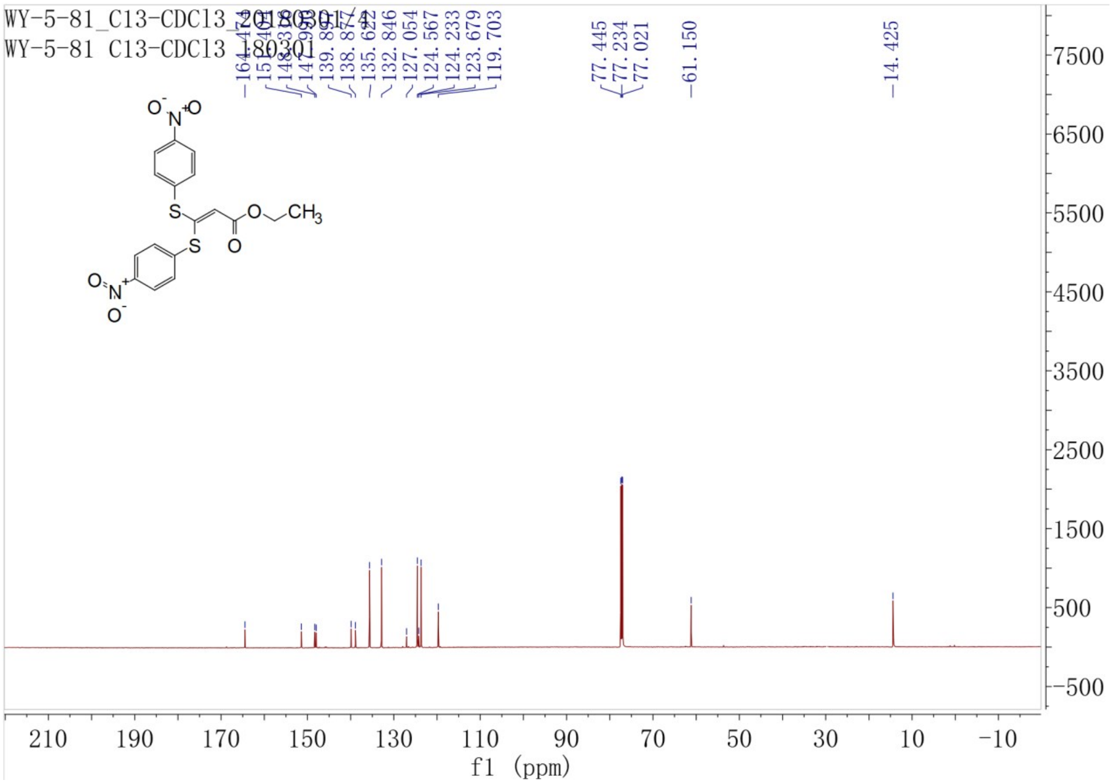
WY-5-81_C13-CDCl3_20180301
139.891
138.877
135.622
132.846
127.054
124.567
124.233
123.679
119.703
77.445
77.234
77.021
61.150
14.425
CH3
f1 (ppm)

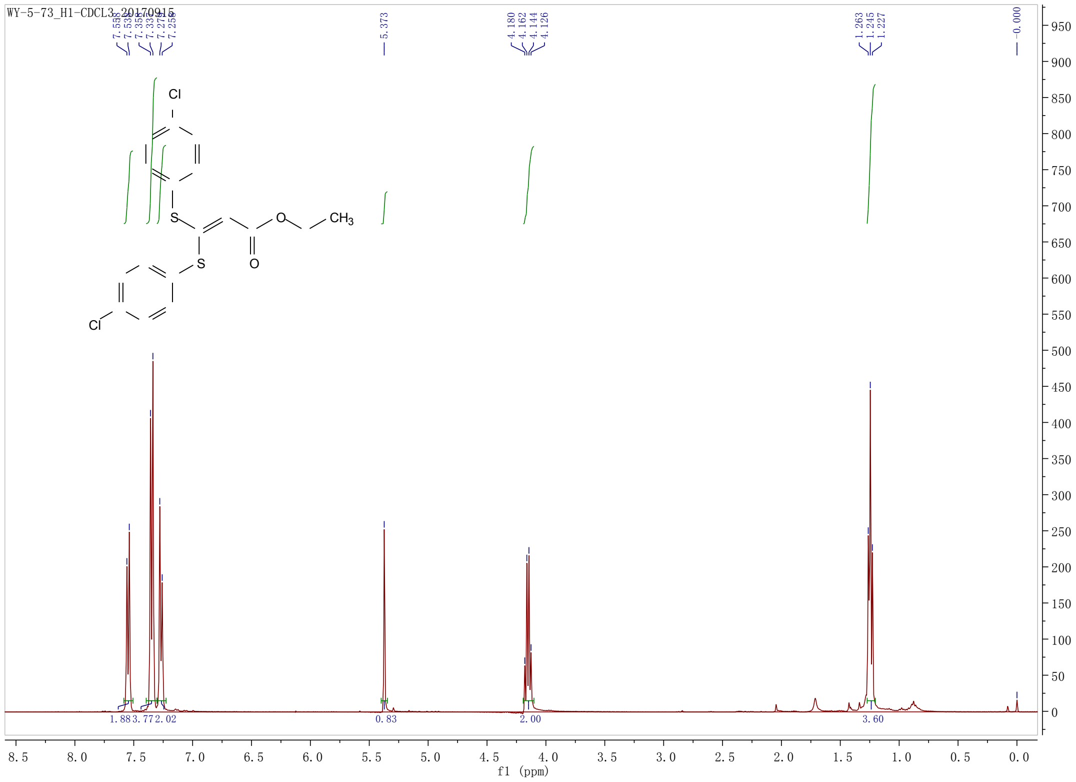
WY-5-73_H1-CDCL3-20170915
5.373
4.180
4.162
4.144
4.126
1.263
1.245
1.227
-0.000
Cl
S
S
O
O
CH3
Cl
1.88
3.77
2.02
0.83
2.00
3.60
f1 (ppm)

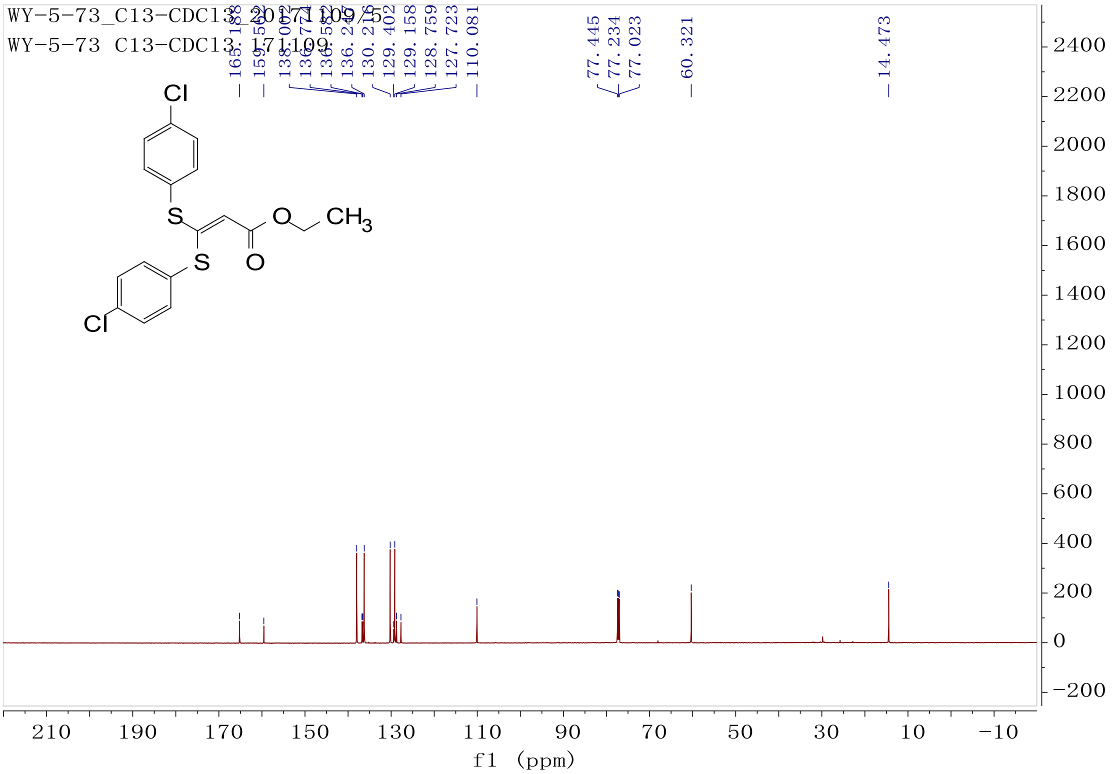
WY-5-73_C13-CDCl3
129.402
129.158
128.759
127.723
110.081
77.445
77.234
77.023
60.321
14.473
Cl
S
S
O
CH3
O
Cl
f1 (ppm)

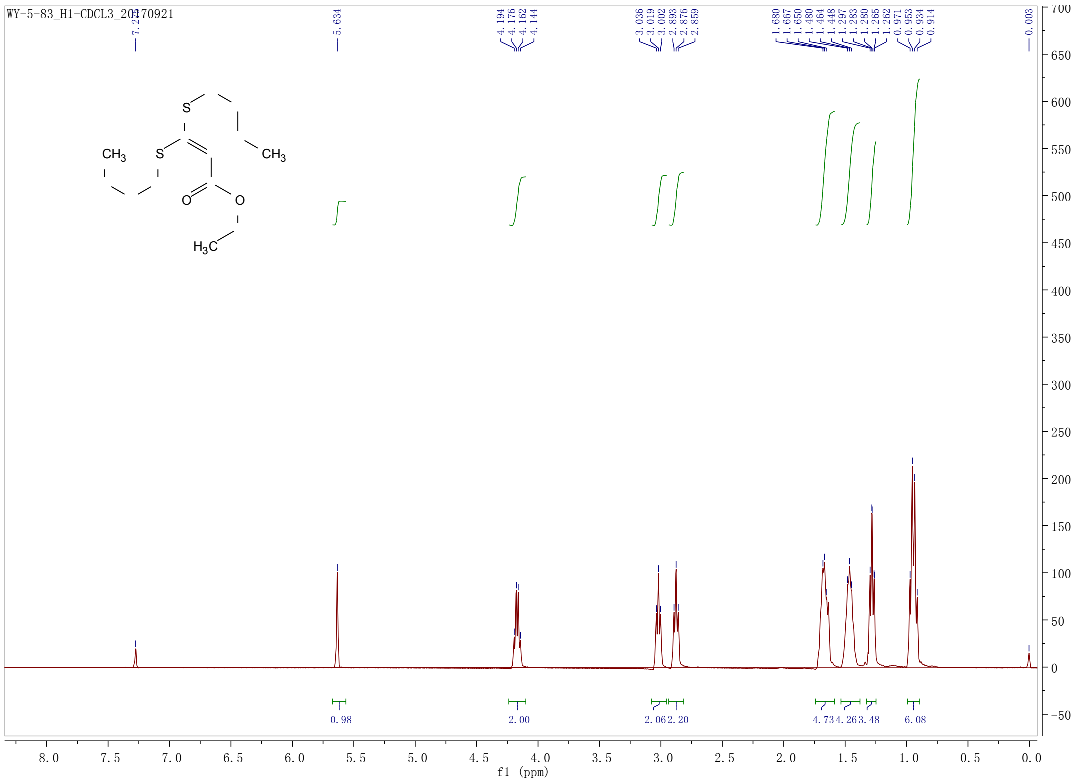
WY-5-83_H1-CDCL3_20170921
5.634
4.194
4.176
4.162
4.144
3.036
3.019
3.002
2.893
2.876
2.859
1.680
1.667
1.650
1.480
1.464
1.448
1.297
1.283
1.280
1.265
1.262
0.971
0.953
0.934
0.914
0.003
0.98
2.00
2.06
2.20
4.73
4.26
3.48
6.08
f1 (ppm)

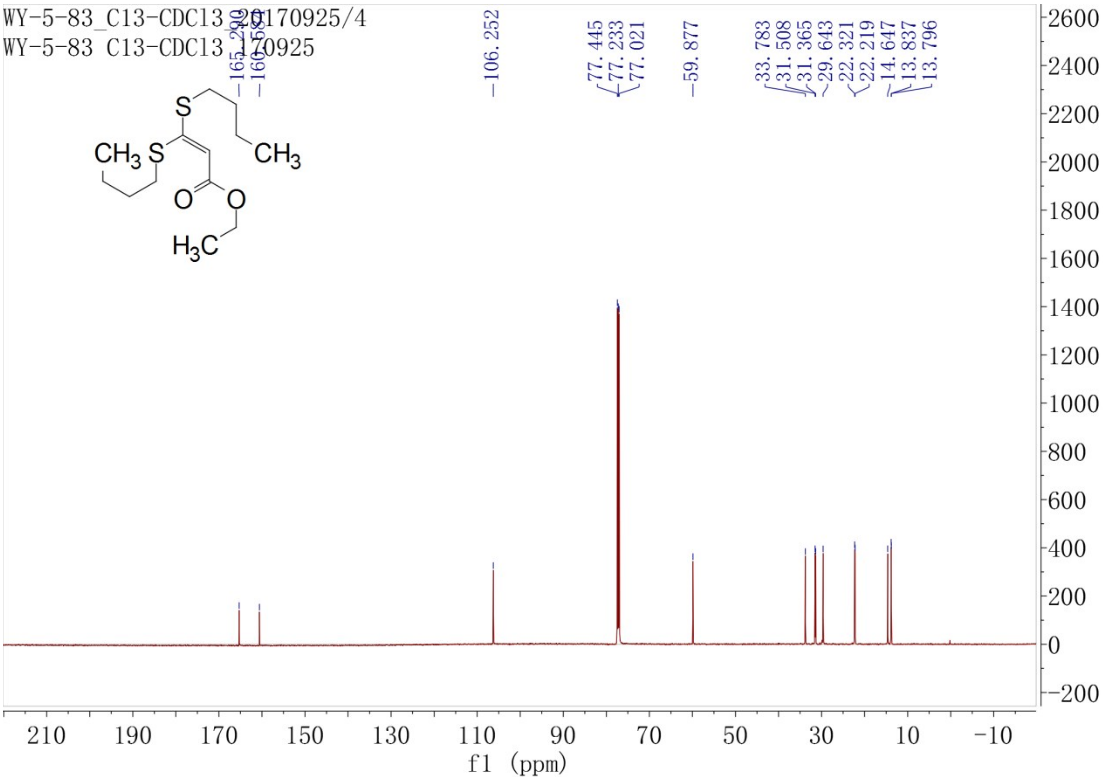
WY-5-83_C13-CDC13_20170925/4
WY-5-83 C13-CDC13 170925
106.252
77.445
77.233
77.021
59.877
33.783
31.508
31.365
29.643
22.321
22.219
14.647
13.837
13.796
f1 (ppm)

3ah

4ah

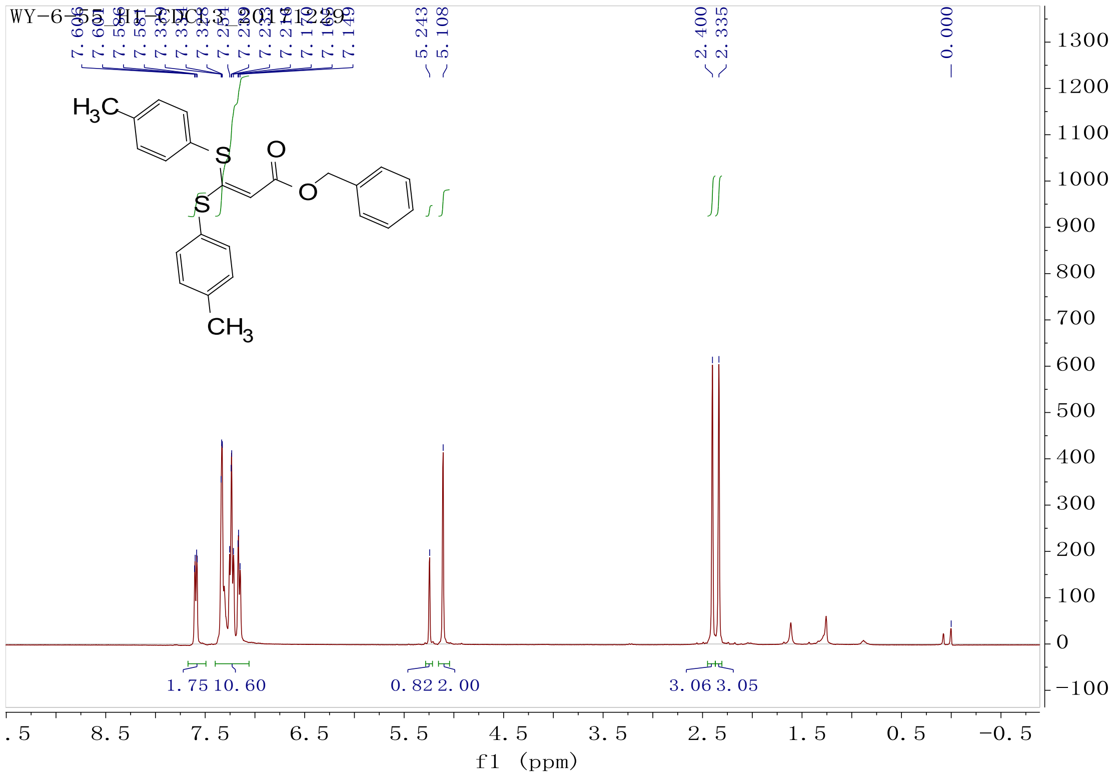

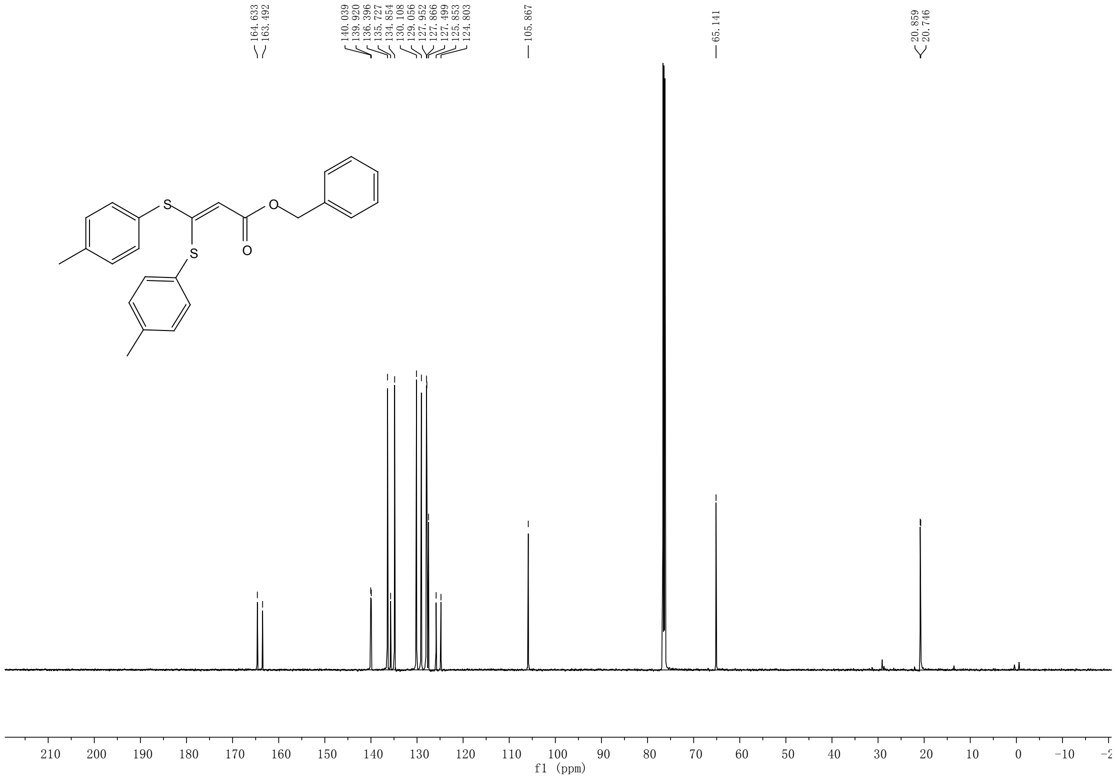

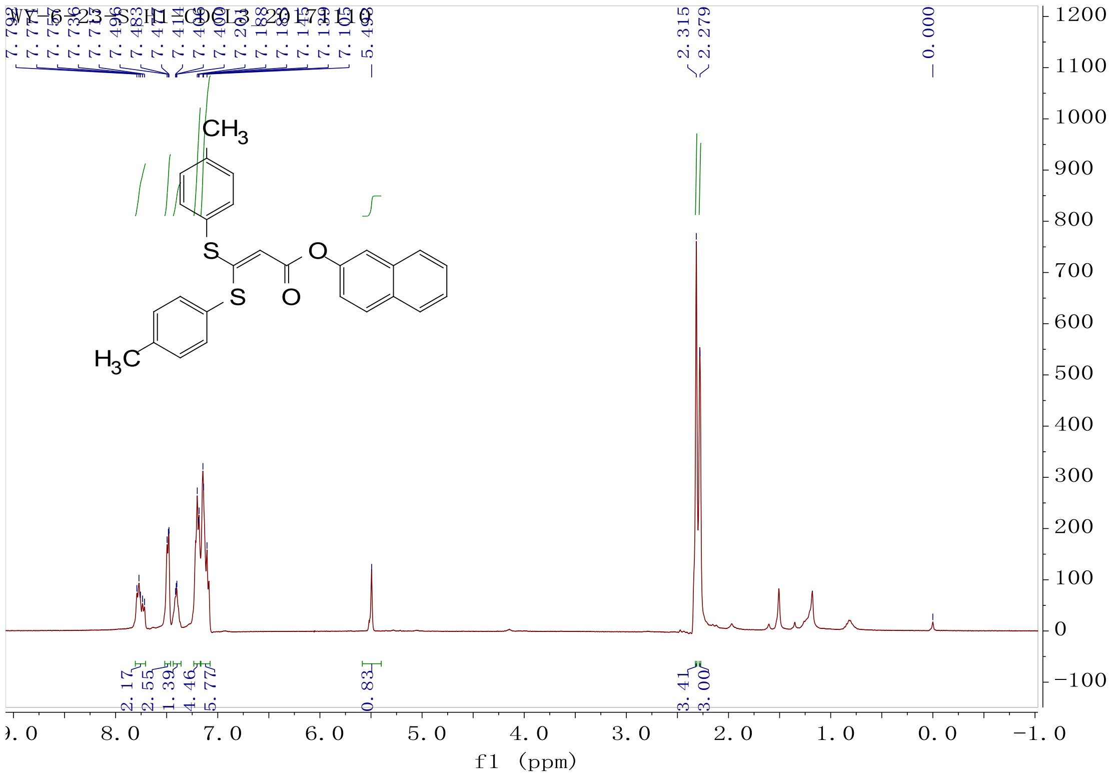
2.315
2.279
0.000
5.498
f1 (ppm)

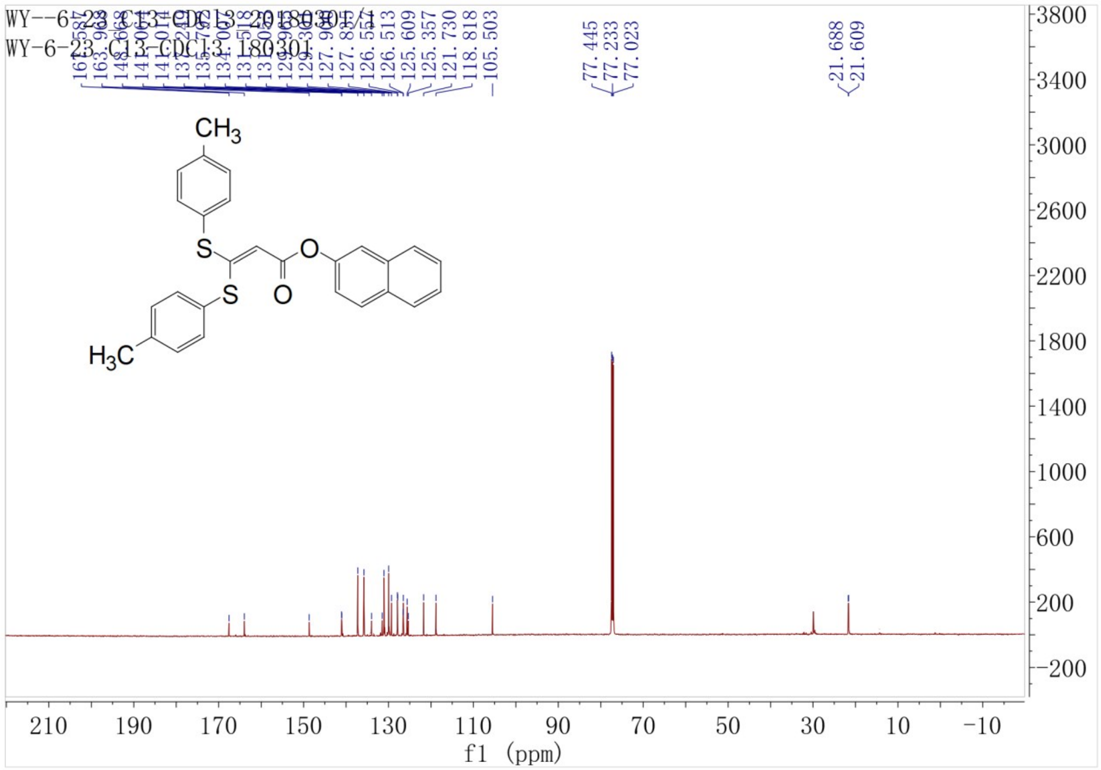
WY-6-23 C13 CDCl3 180301
125.609
125.357
121.730
118.818
105.503
77.445
77.233
77.023
21.688
21.609
f1 (ppm)

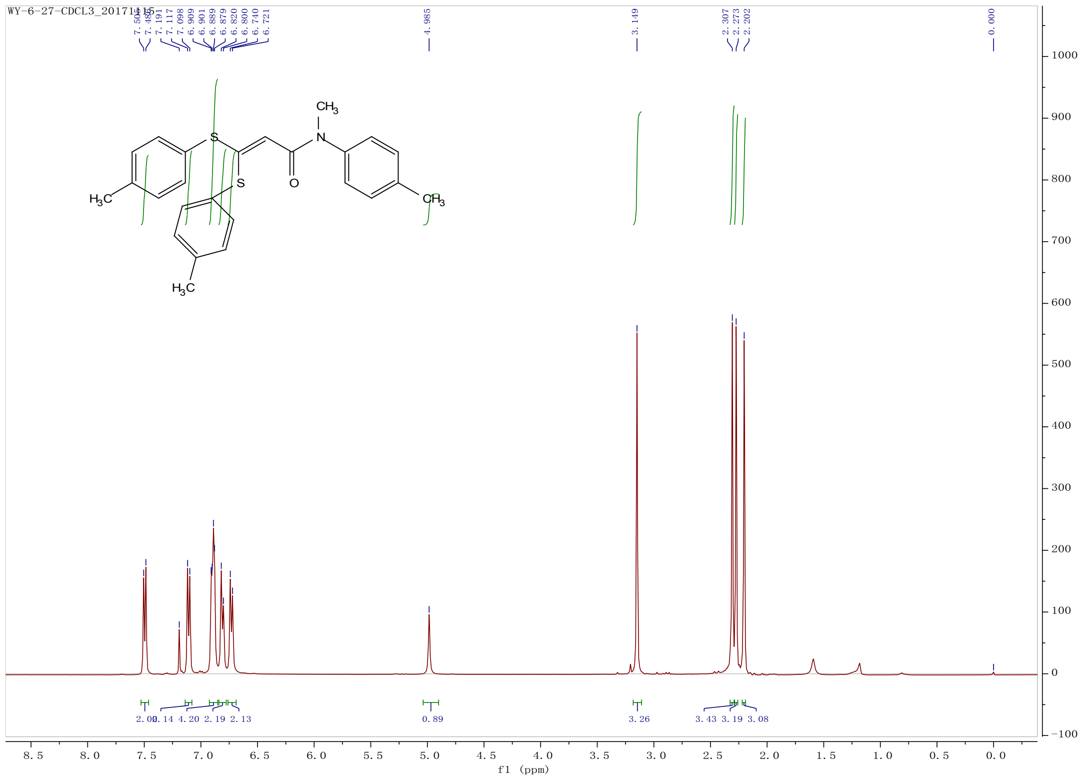

7.191
7.117
7.098
6.909
6.901
6.889
6.879
6.820
6.800
6.740
6.721
4.985
3.149
2.307
2.273
2.202
0.000
4.20
2.19
2.13
0.89
3.26
3.43
3.19
3.08
f1 (ppm)

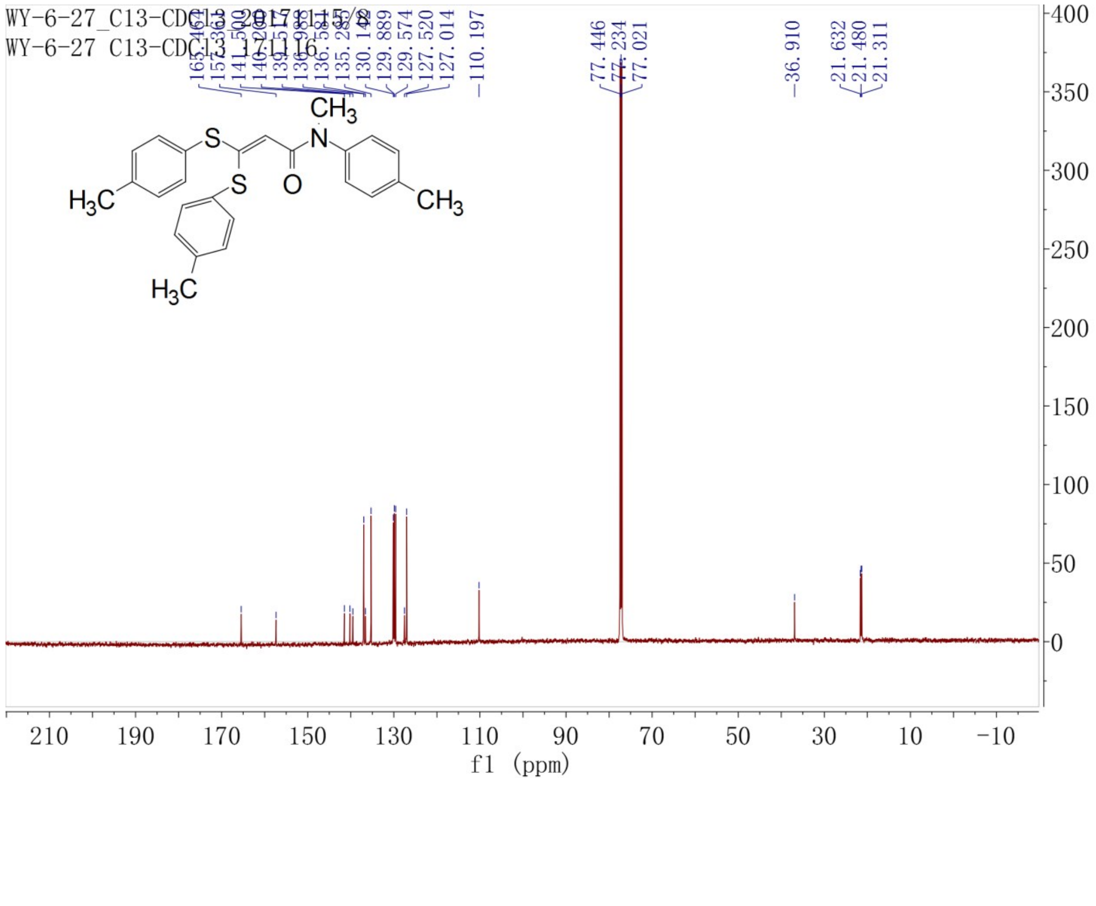

129.889
129.574
127.520
127.014
110.197
77.446
77.021
36.910
21.632
21.480
21.311
f1 (ppm)

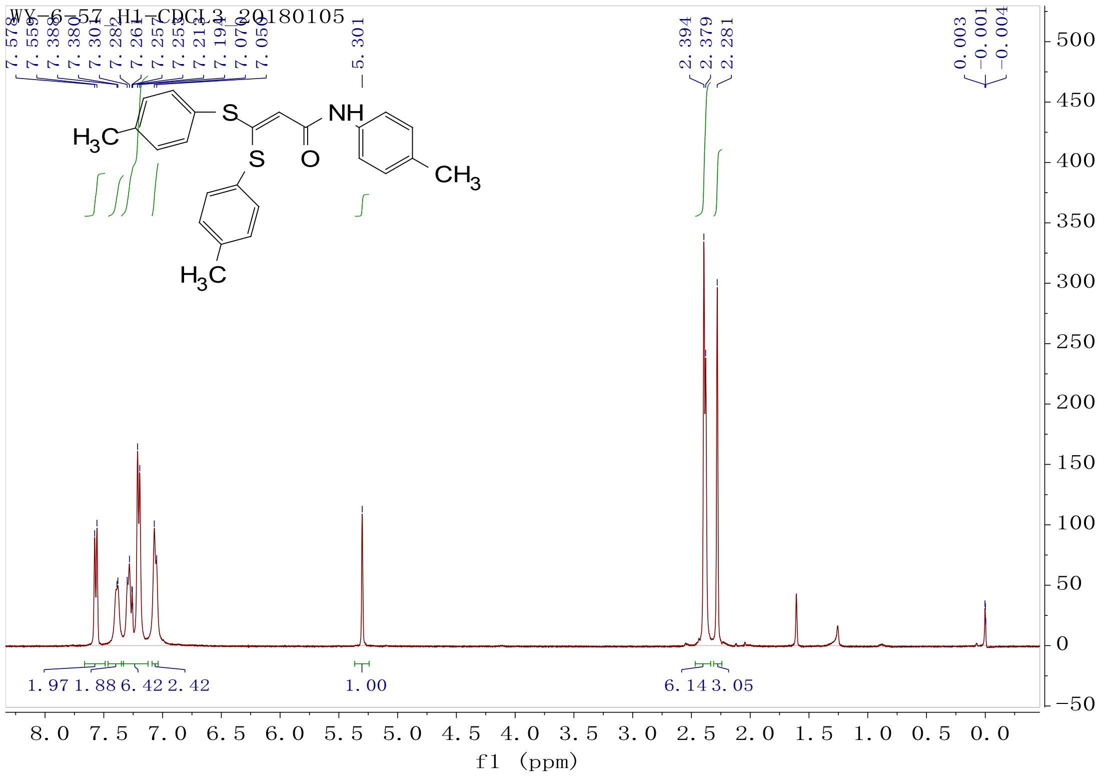

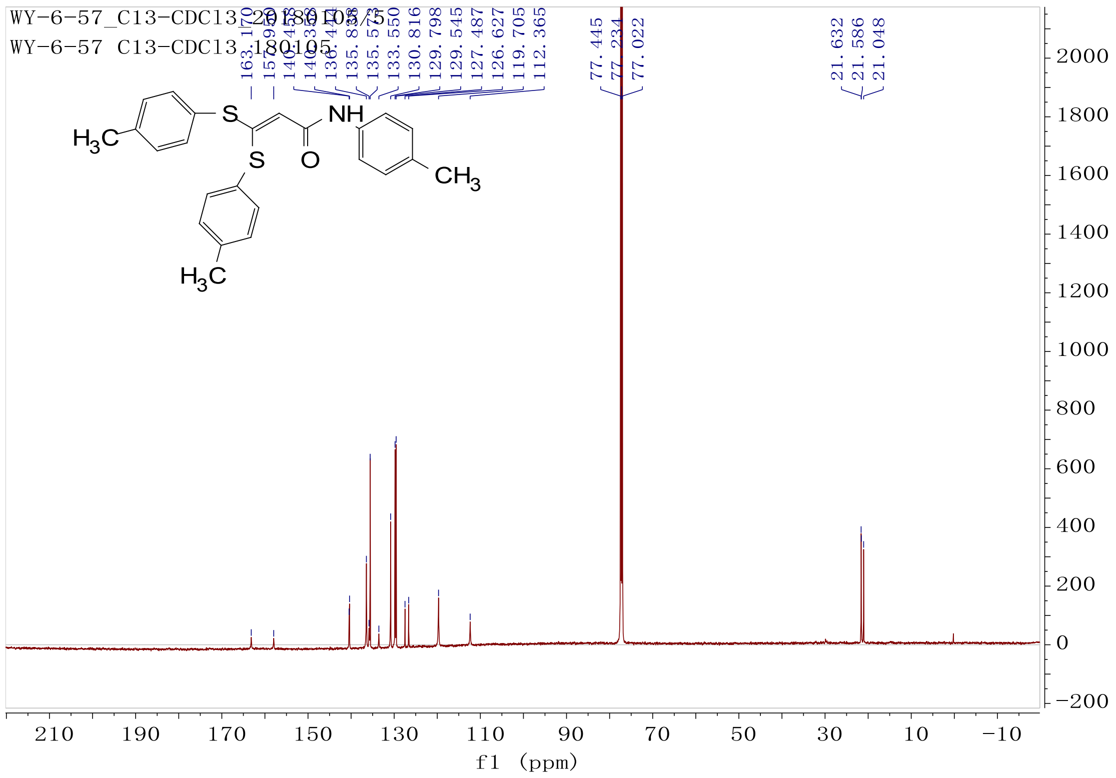

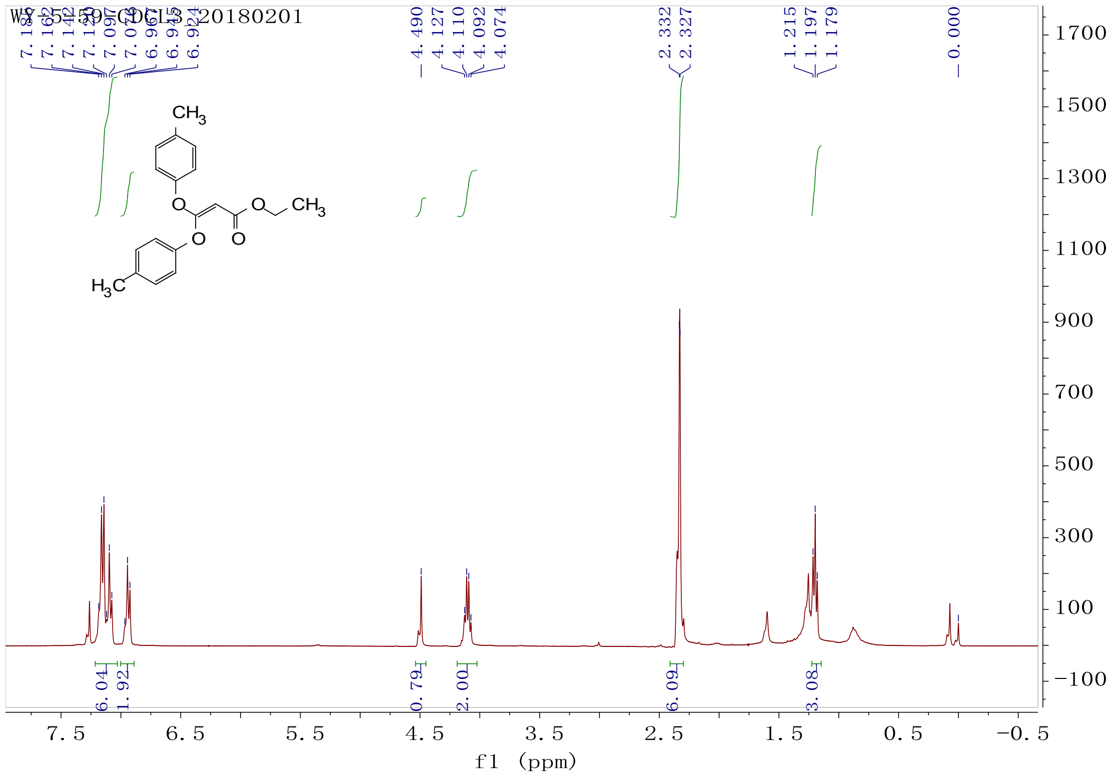

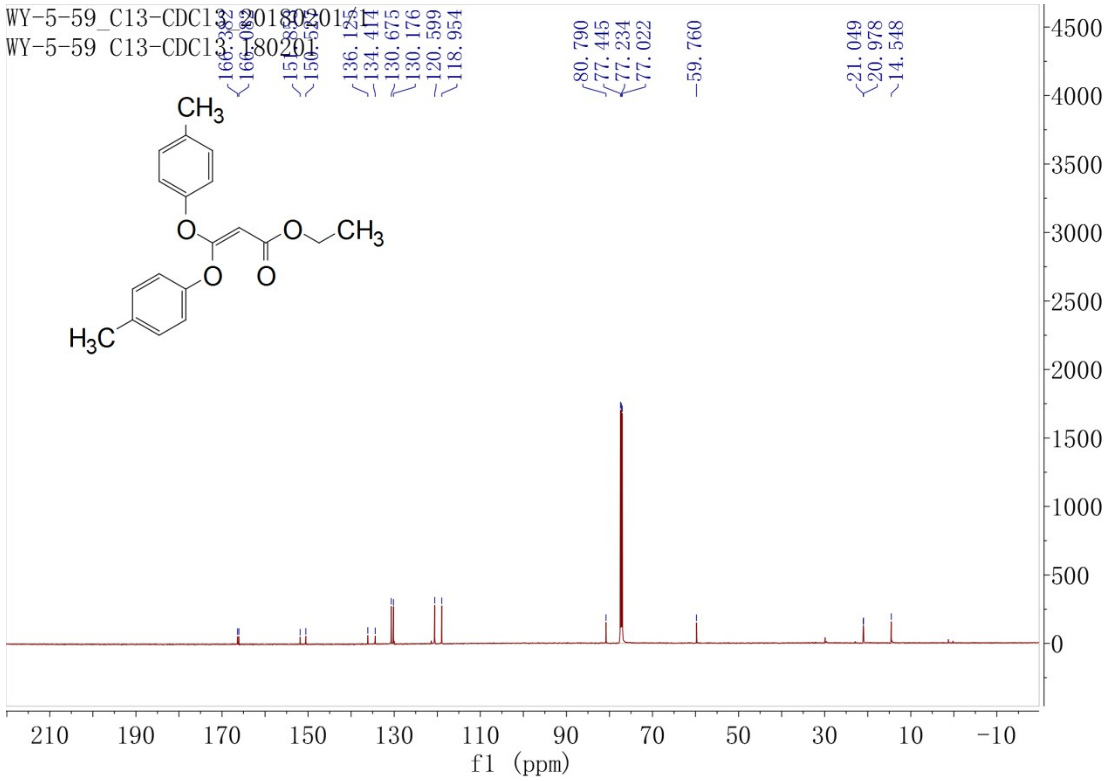

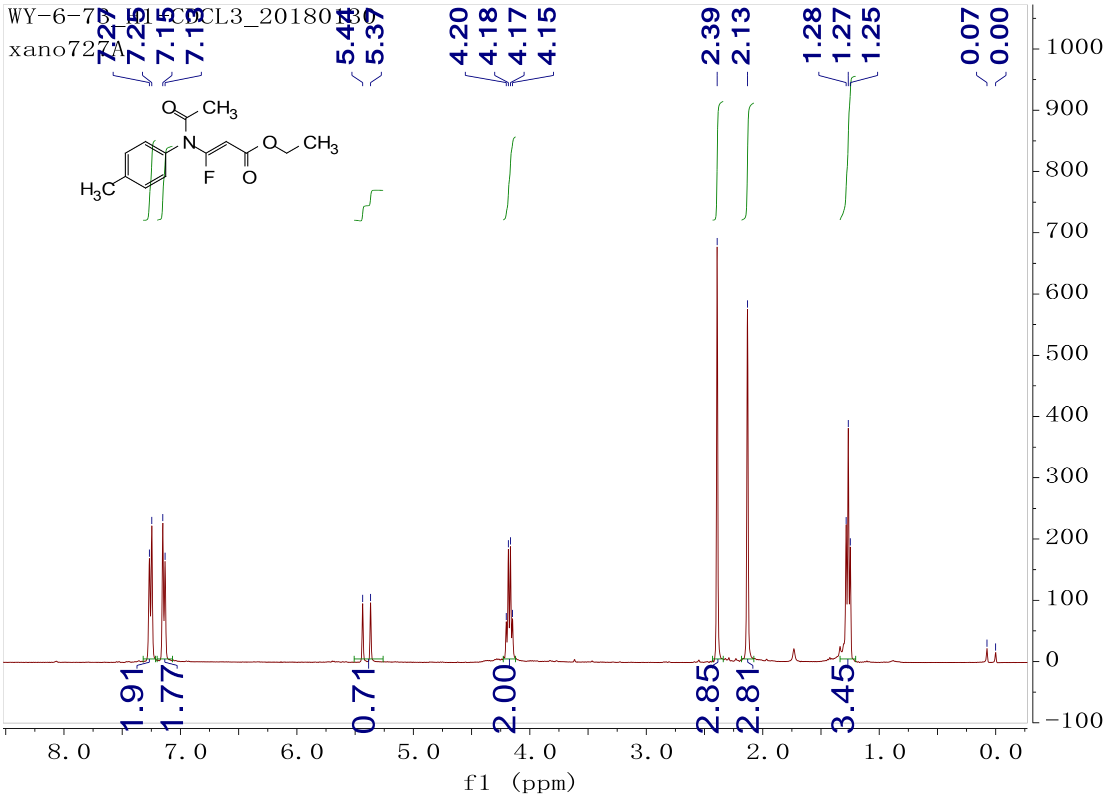
WY-6-73_H1-CDCL3_20180130
xano727A
7.27
7.25
7.15
7.13
5.44
5.37
4.20
4.18
4.17
4.15
2.39
2.13
1.28
1.27
1.25
0.07
0.00
1.91
1.77
0.71
2.00
2.85
2.81
3.45
f1 (ppm)

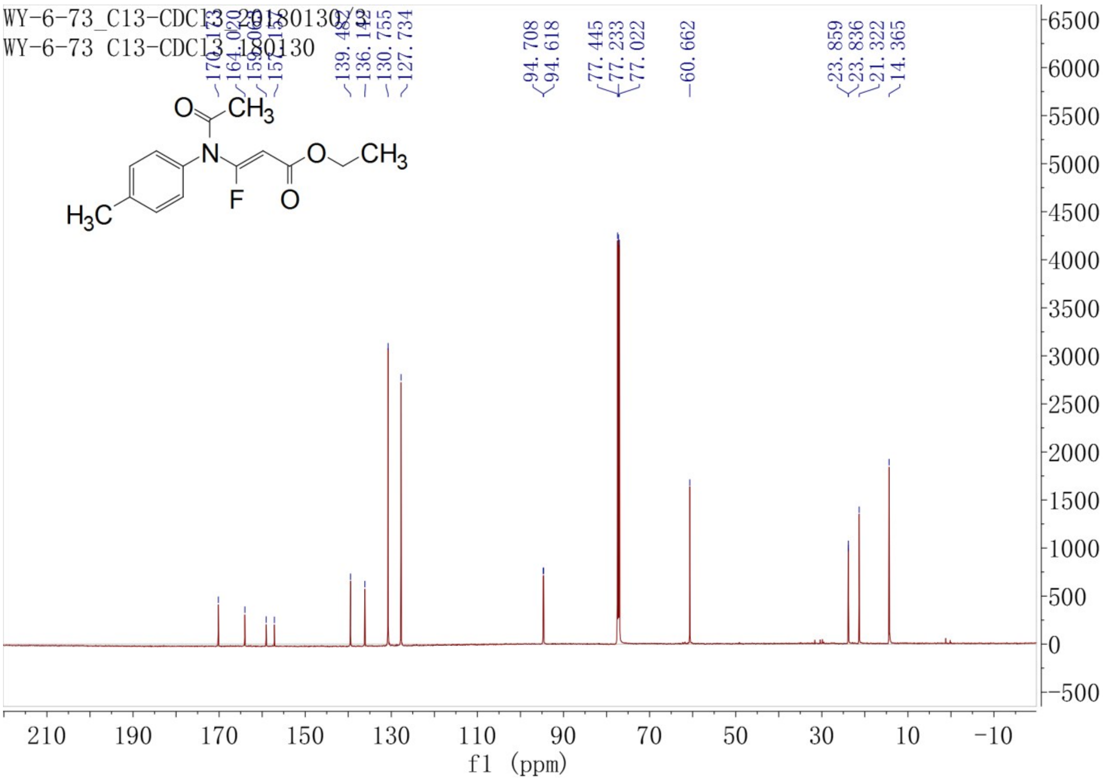
WY-6-73_C13-CDCl3_20180130/3
WY-6-73 C13-CDCl3 180130
139.482
136.142
130.755
127.734
94.708
94.618
77.445
77.233
77.022
60.662
23.859
23.836
21.322
14.365
f1 (ppm)

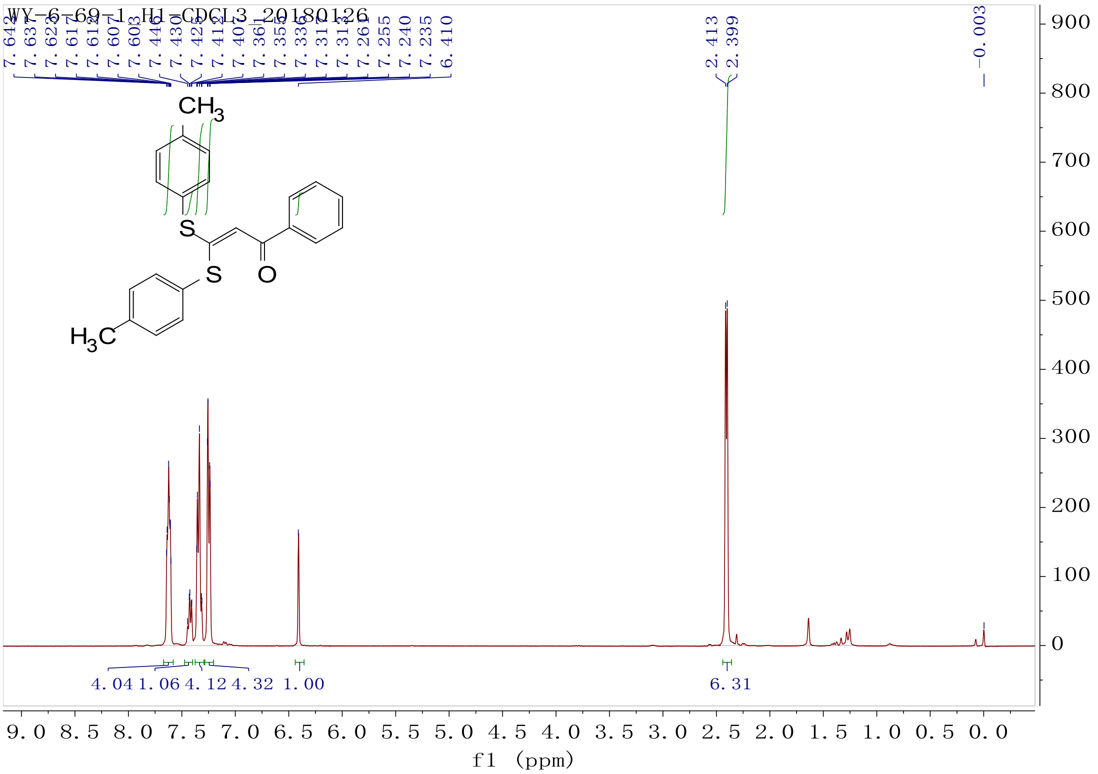
WY-6-69-1_H1-CDCl3_20180126
7.642
7.637
7.622
7.617
7.607
7.603
7.446
7.430
7.411
7.407
7.361
7.355
7.336
7.311
7.261
7.255
7.240
7.235
6.410
2.413
2.399
-0.003
CH3
S
S
O
H3C
4.04
1.06
4.12
4.32
1.00
6.31
f1 (ppm)

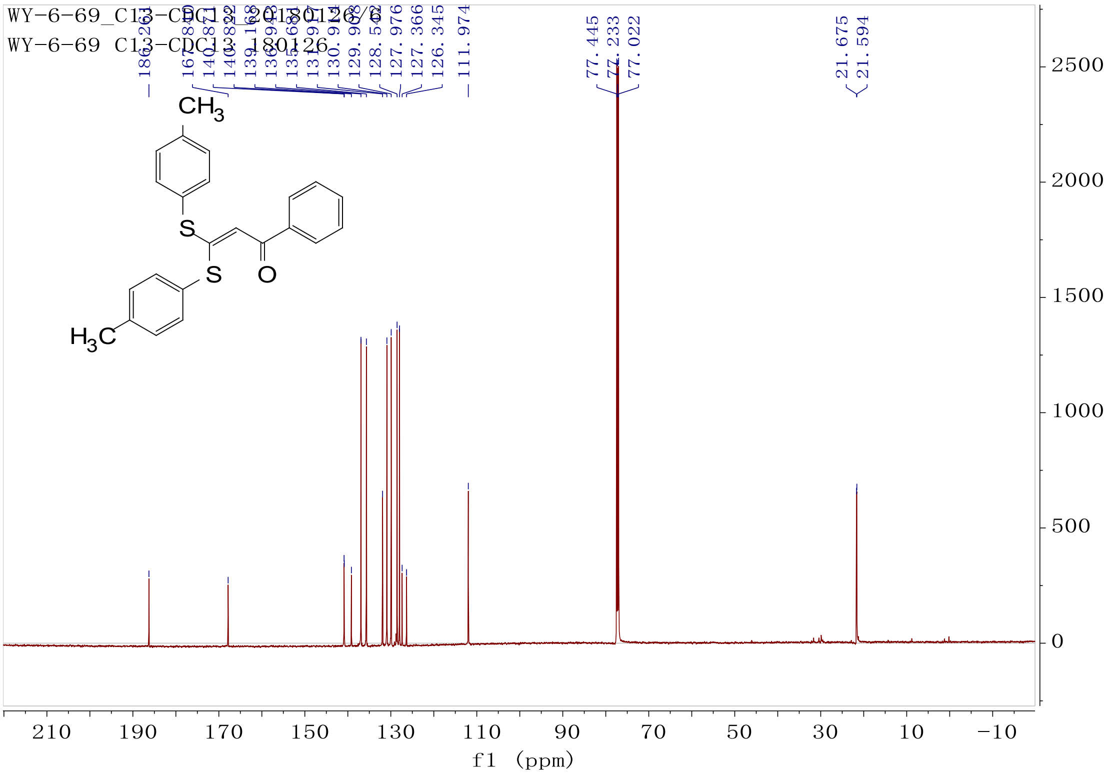
WY-6-69_C13-CDCl3_20180126/6
WY-6-69 C13-CDCl3 180126
139.168
127.976
127.366
126.345
111.974
77.445
77.233
77.022
21.675
21.594
CH3
S
S
O
H3C
f1 (ppm)

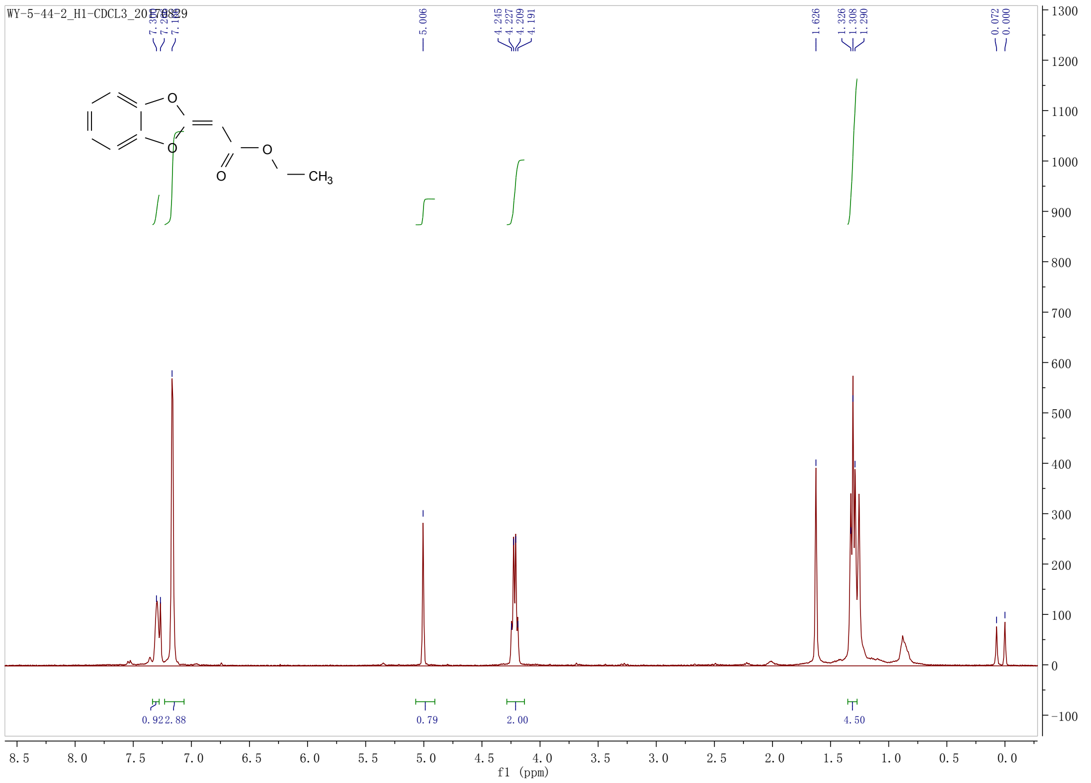
WY-5-44-2_H1-CDCL3_20170829
5.006
4.245
4.227
4.209
4.191
1.626
1.326
1.308
1.290
0.072
0.000
CH3
O
0.92
2.88
0.79
2.00
4.50
f1 (ppm)

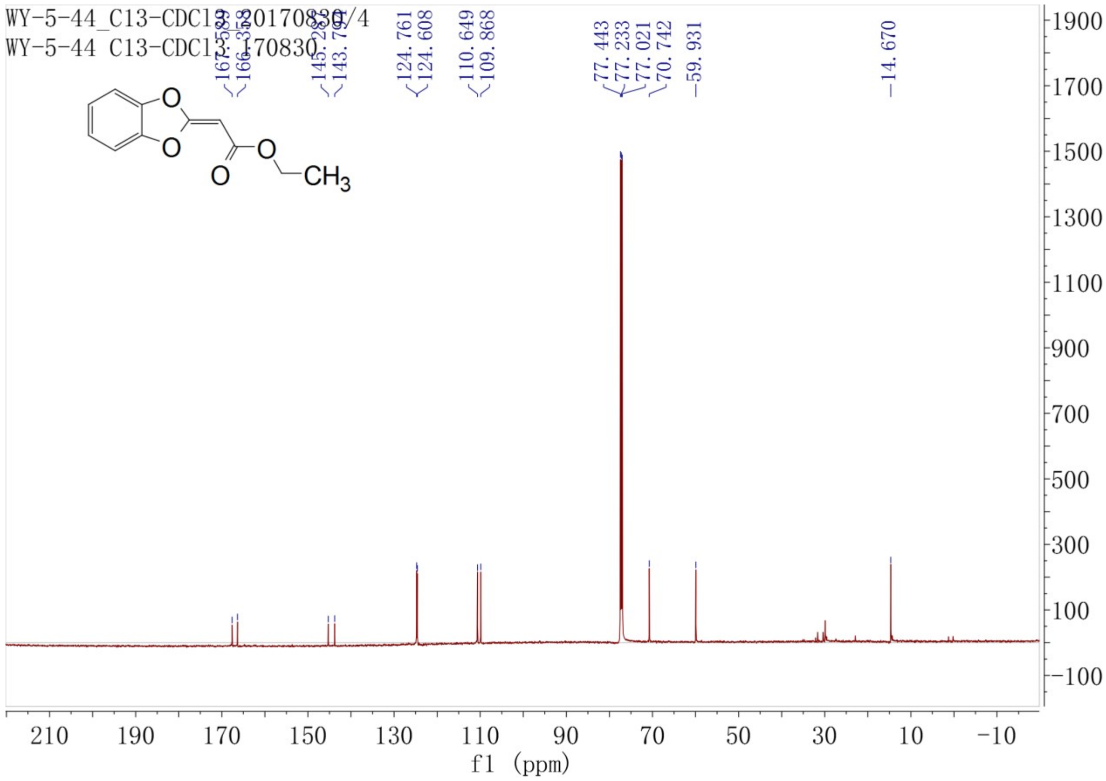
WY-5-44_C13-CDCl3_20170830/4
124.761
124.608
110.649
109.868
77.443
77.233
77.021
70.742
59.931
14.670
CH3
O
f1 (ppm)

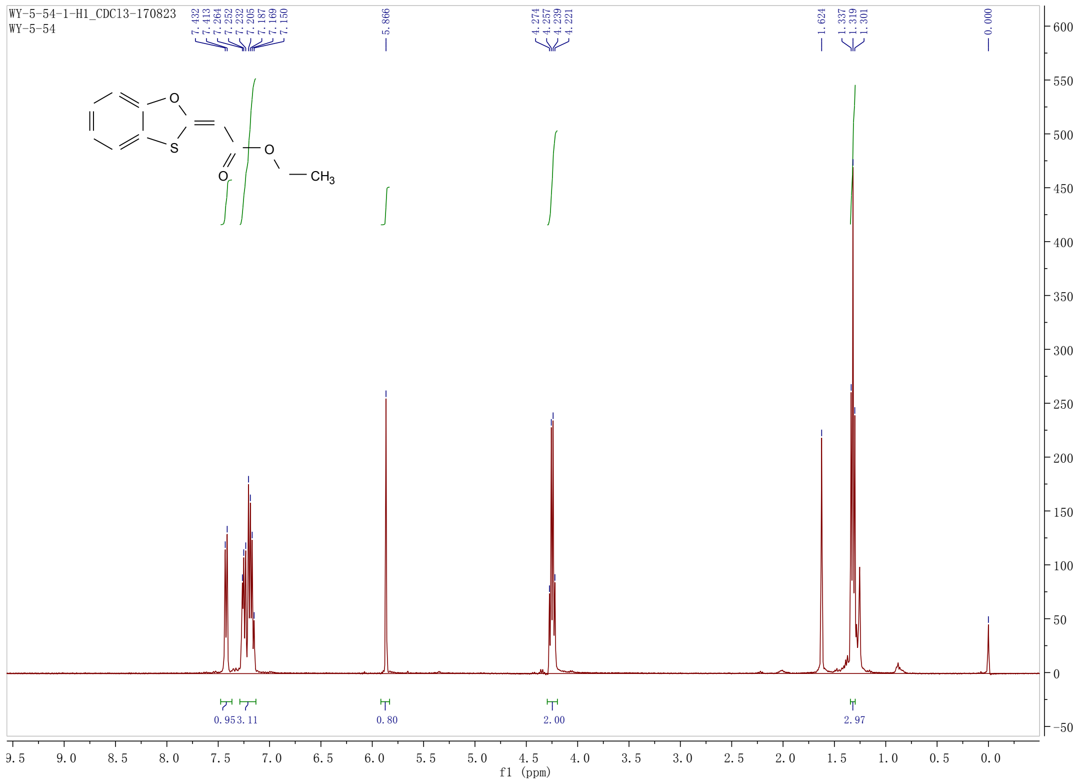
WY-5-54-1-H1_CDC13-170823
WY-5-54
7.432
7.413
7.264
7.252
7.232
7.205
7.187
7.169
7.150
5.866
4.274
4.257
4.239
4.221
1.624
1.337
1.319
1.301
0.000
O
S
O
O
CH3
0.95
3.11
0.80
2.00
2.97
f1 (ppm)

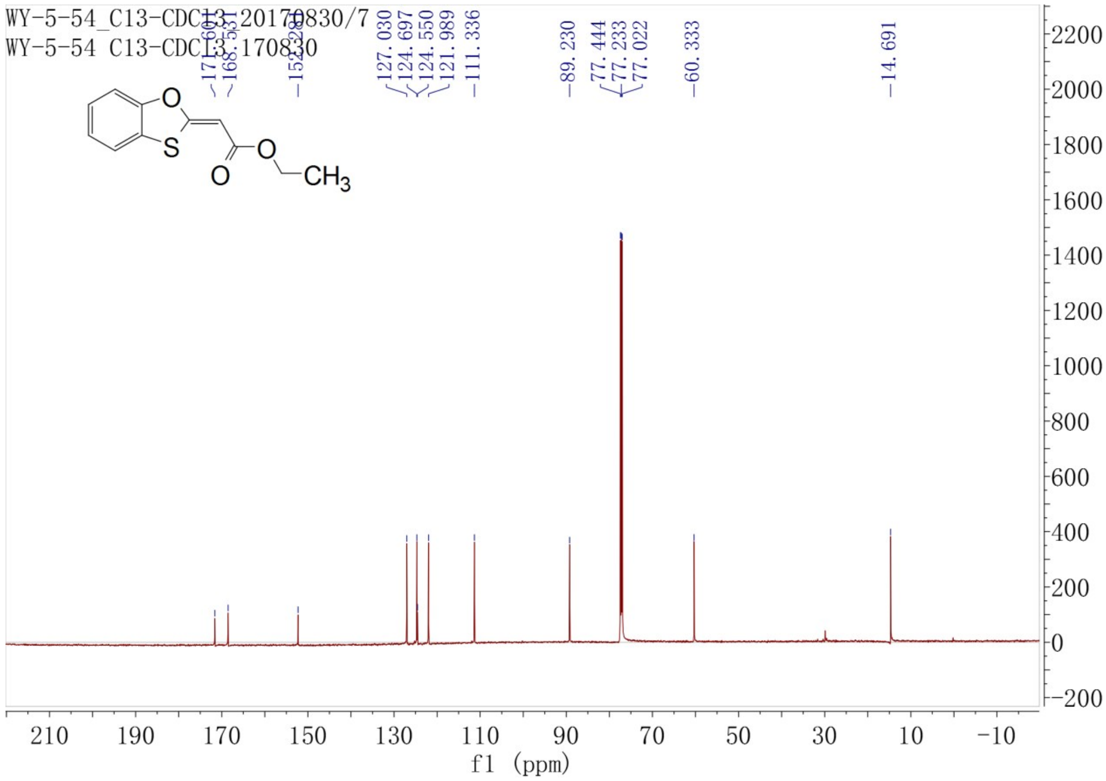
WY-5-54_C13-CDC13-20170830/7
WY-5-54 C13-CDC13-170830
127.030
124.697
124.550
121.989
111.336
89.230
77.444
77.233
77.022
60.333
14.691
O
S
O
O
CH3
f1 (ppm)

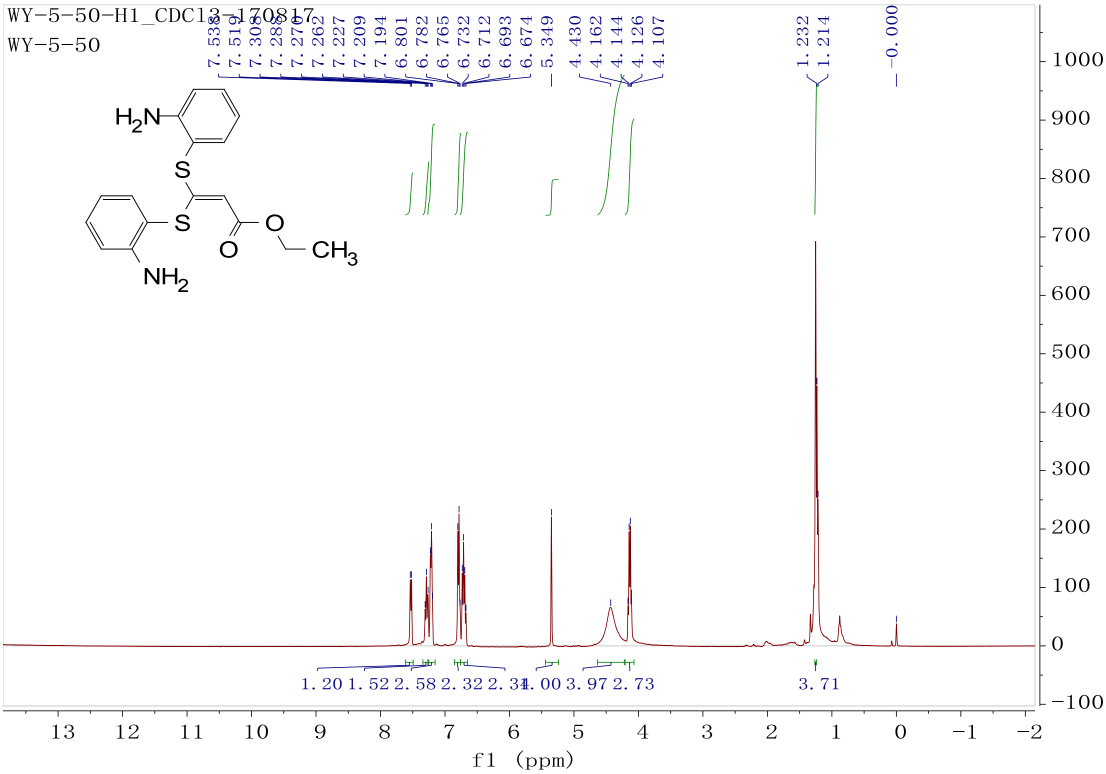
WY-5-50-H1_CDCl3-170817
WY-5-50
7.538 7.519 7.308 7.288 7.276 7.262 7.227 7.209 7.194 6.801 6.782 6.765 6.732 6.712 6.693 6.674
5.349
4.430 4.162 4.144 4.126 4.107
1.232 1.214
-0.000
1.20 1.52 2.58 2.32 2.31 1.00 3.97 2.73 3.71
f1 (ppm)

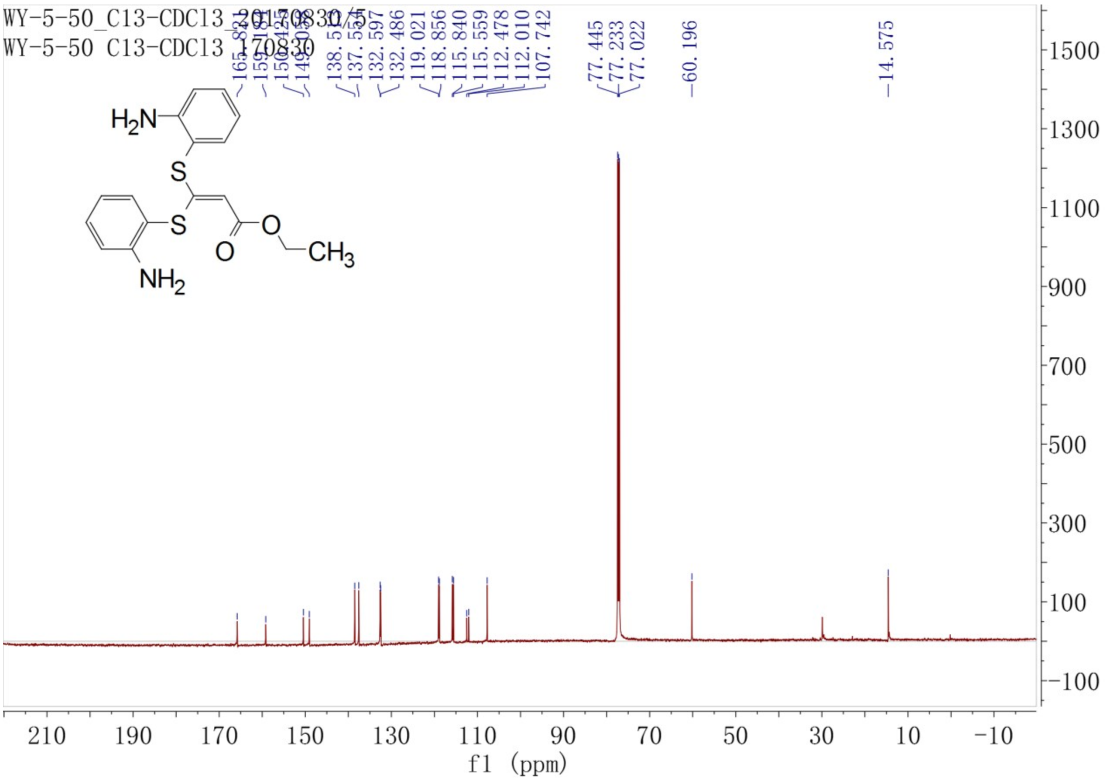
WY-5-50_C13-CDCl3_20170830/5
WY-5-50 C13-CDCl3 170830
138.51 137.53 132.597 132.486 119.021 118.856 115.840 115.559 112.478 112.010 107.742
77.445 77.233 77.022
60.196
14.575
f1 (ppm)

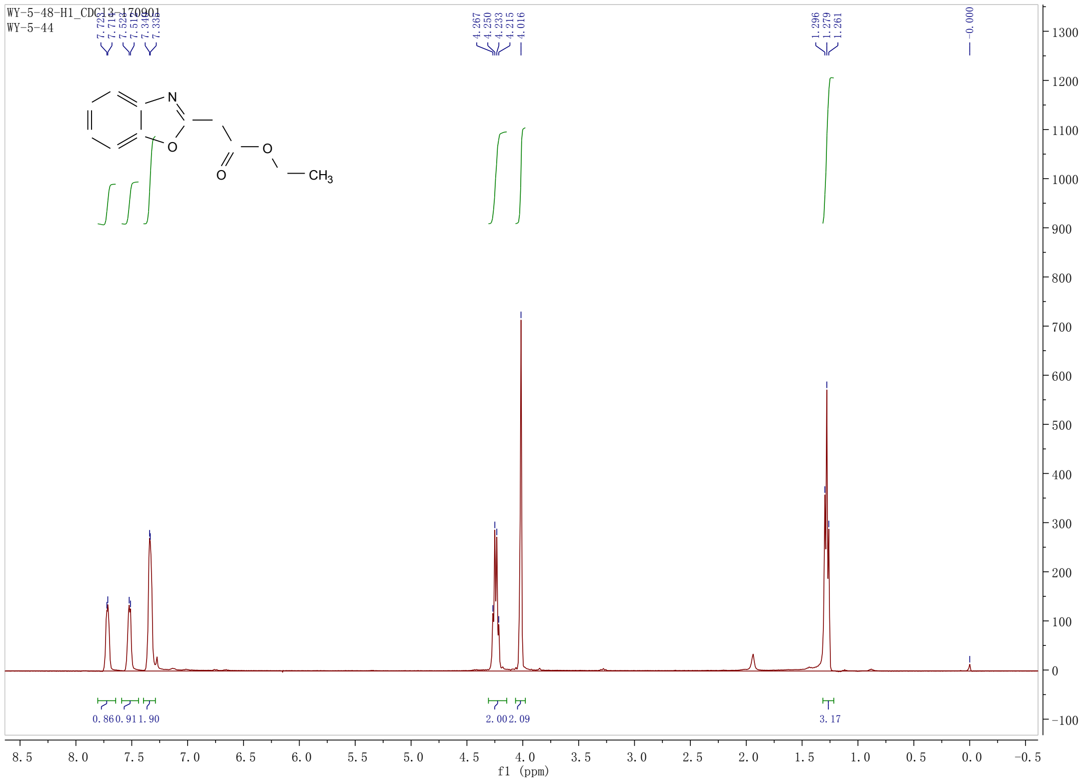
WY-5-48-H1_CDCl3_170901
WY-5-44
f1 (ppm)

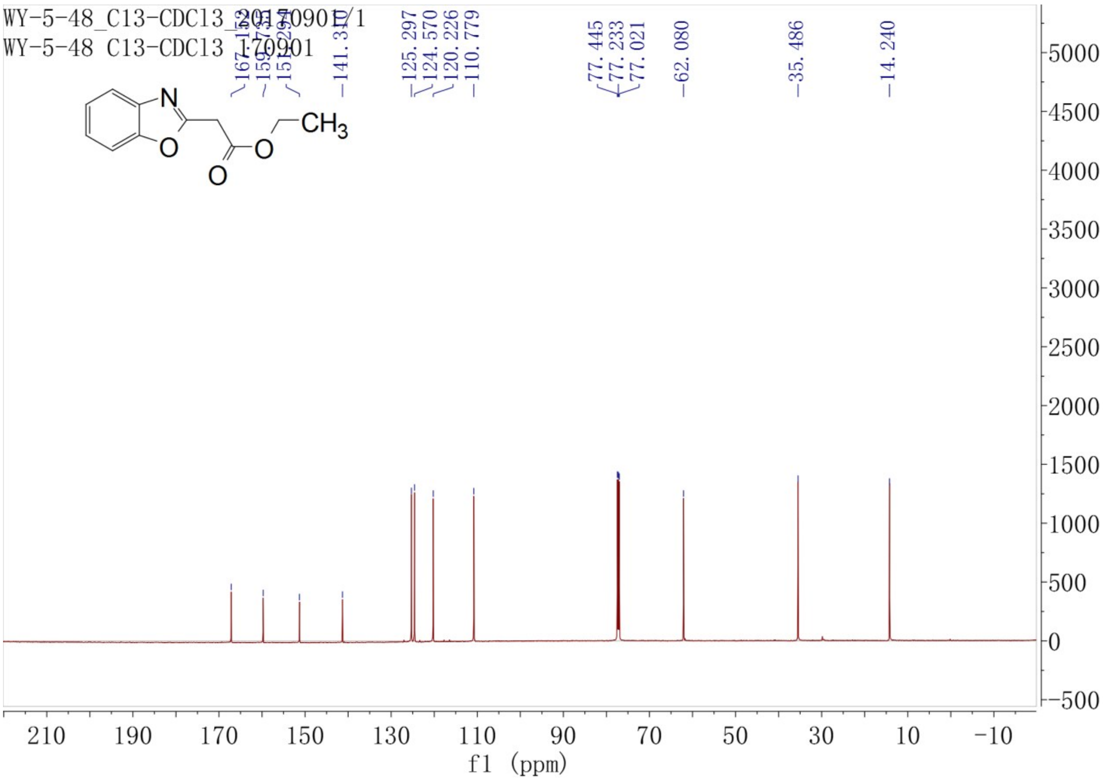
WY-5-48_C13-CDCl3_20170901/1
WY-5-48 C13-CDCl3 170901
141.311
125.297
124.570
120.226
110.779
77.445
77.233
77.021
62.080
35.486
14.240
f1 (ppm)

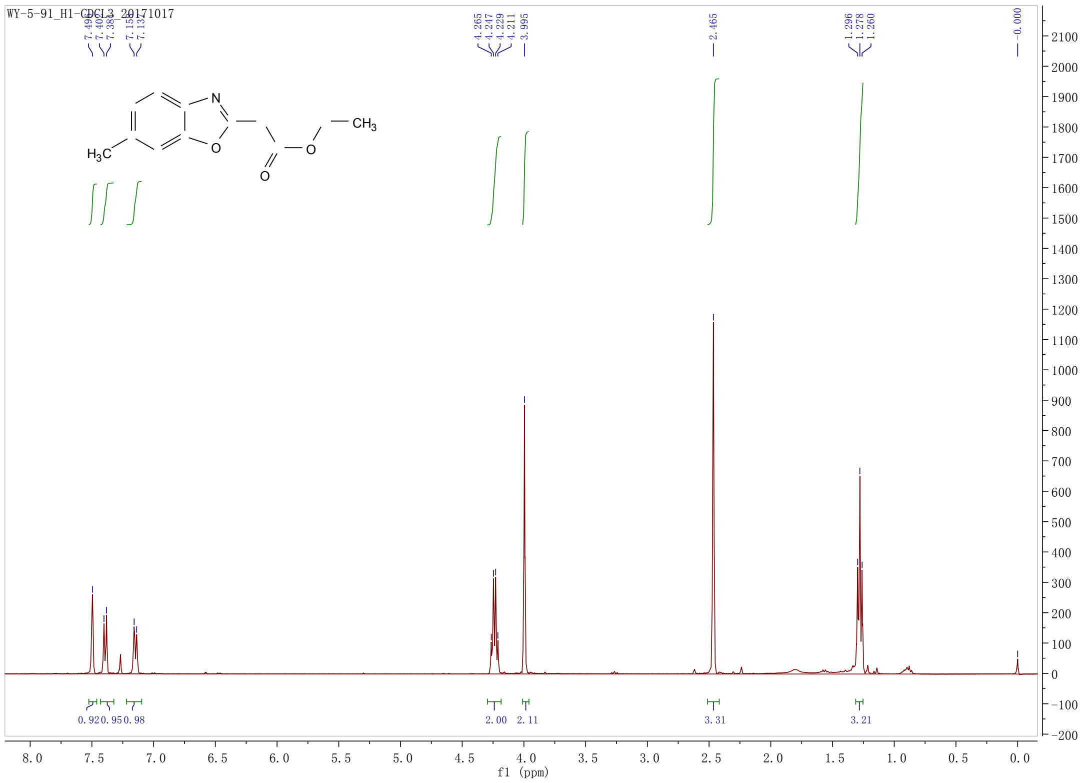
WY-5-91_H1-CDCl3_20171017
7.490 7.402 7.384 7.151 7.133
4.265 4.247 4.229 4.211 3.995
2.465
1.296 1.278 1.260
0.000
0.92 0.95 0.98 2.00 2.11 3.31 3.21
f1 (ppm)

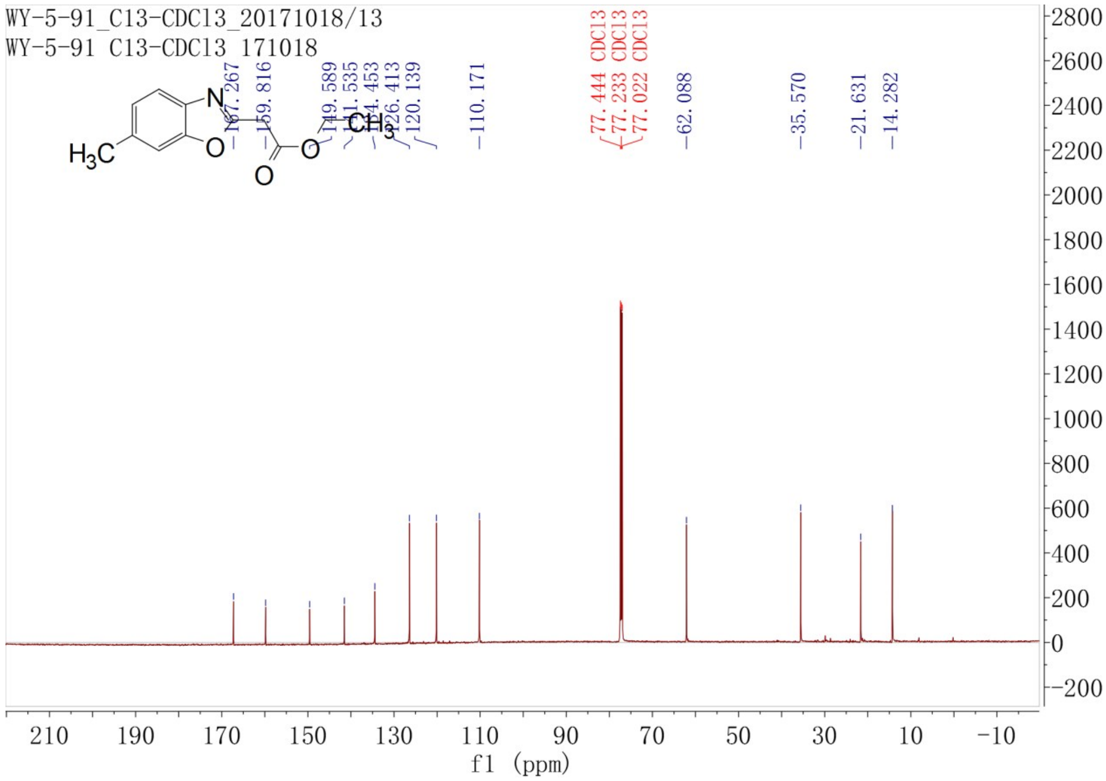
WY-5-91_C13-CDCl3_20171018/13
WY-5-91 C13-CDCl3 171018
167.267 159.816 149.589 141.535 134.453 126.413 120.139 110.171
77.444 CDCl3 77.233 CDCl3 77.022 CDCl3
62.088 35.570 21.631 14.282
f1 (ppm)

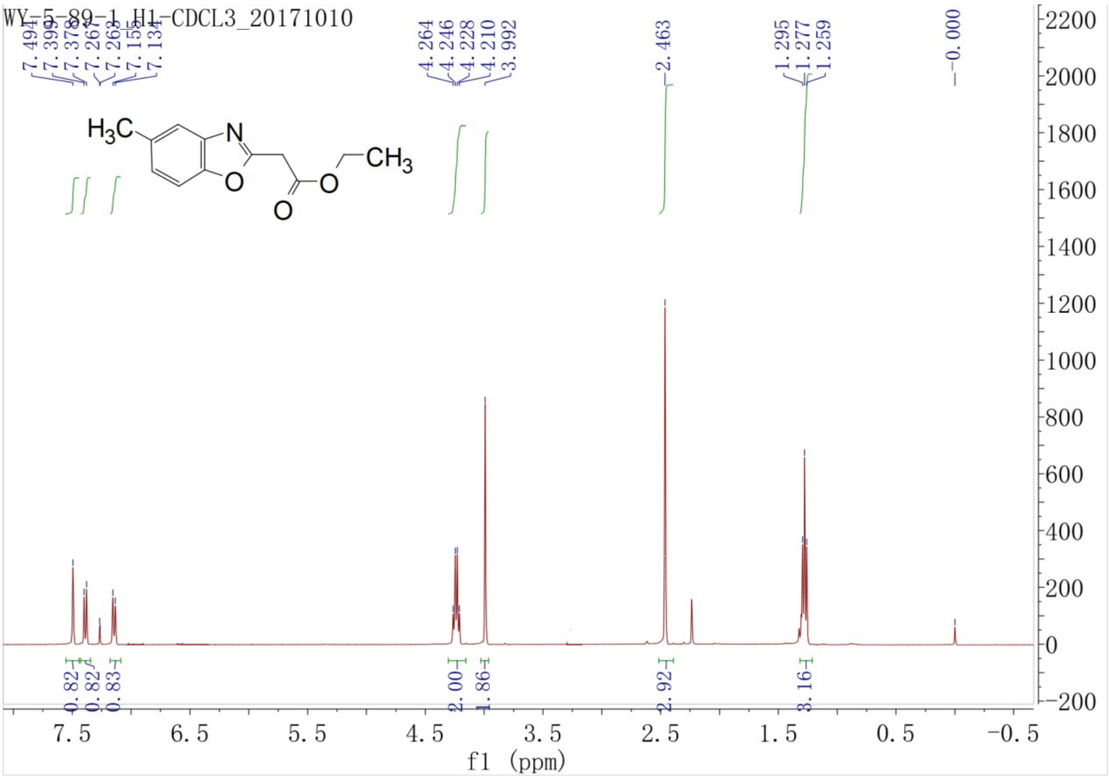
WY-5-89-1 H1-CDCL3_20171010
7.494
7.399
7.378
7.267
7.262
7.155
7.134
4.264
4.246
4.228
4.210
3.992
2.463
1.295
1.277
1.259
-0.000
0.82
0.82
0.83
2.00
1.86
2.92
3.16
f1 (ppm)

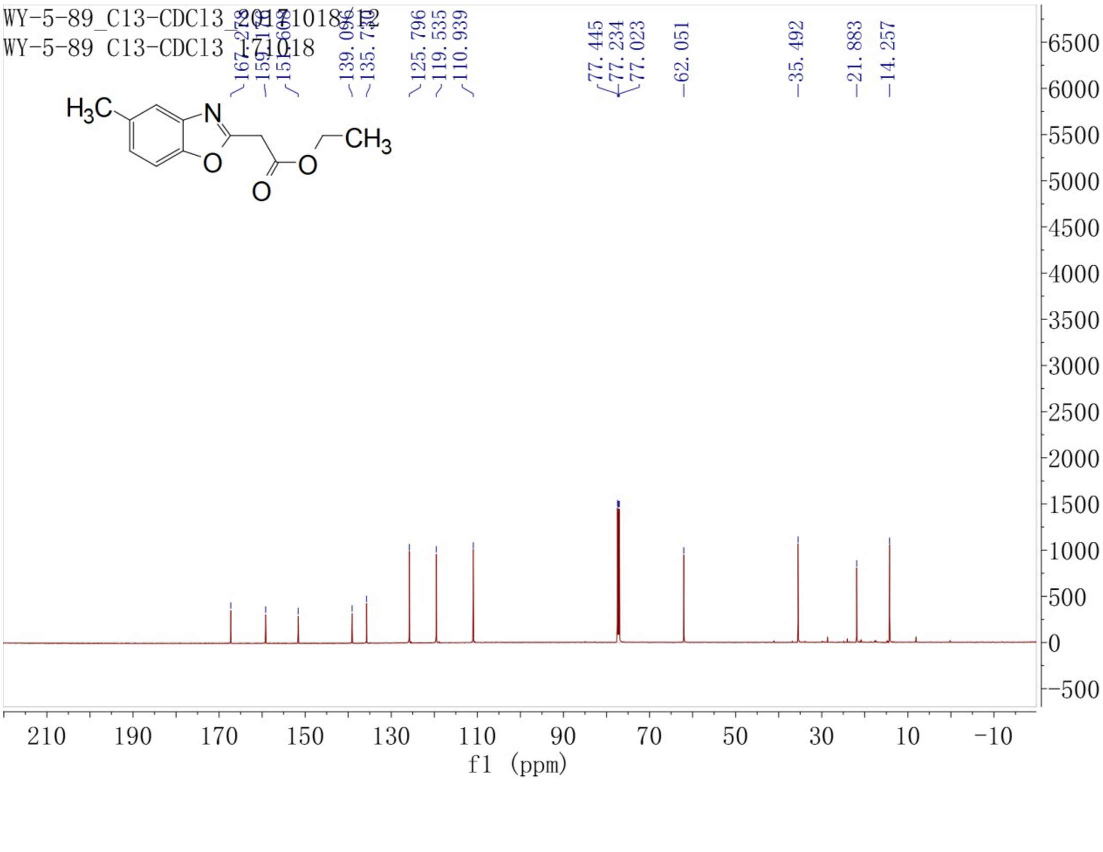
WY-5-89_C13-CDCl3_20171018
WY-5-89 C13-CDCl3 171018
139.096
135.742
125.796
119.535
110.939
77.445
77.234
77.023
62.051
35.492
21.883
14.257
f1 (ppm)

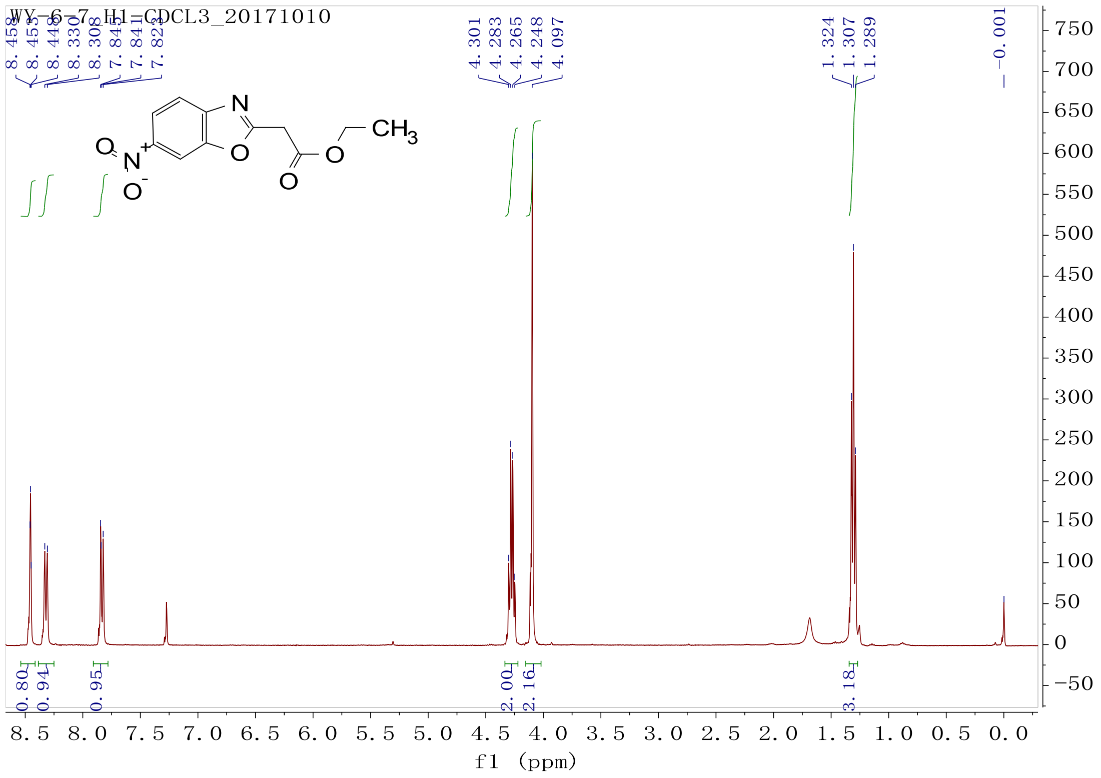
WY-6-7_H1-CDCL3_20171010
8.458
8.453
8.448
8.330
8.308
7.845
7.841
7.823
4.301
4.283
4.265
4.248
4.097
1.324
1.307
1.289
-0.001
CH3
N
O
O
O
N+
O-
0.80
0.94
0.95
2.00
2.16
3.18
f1 (ppm)

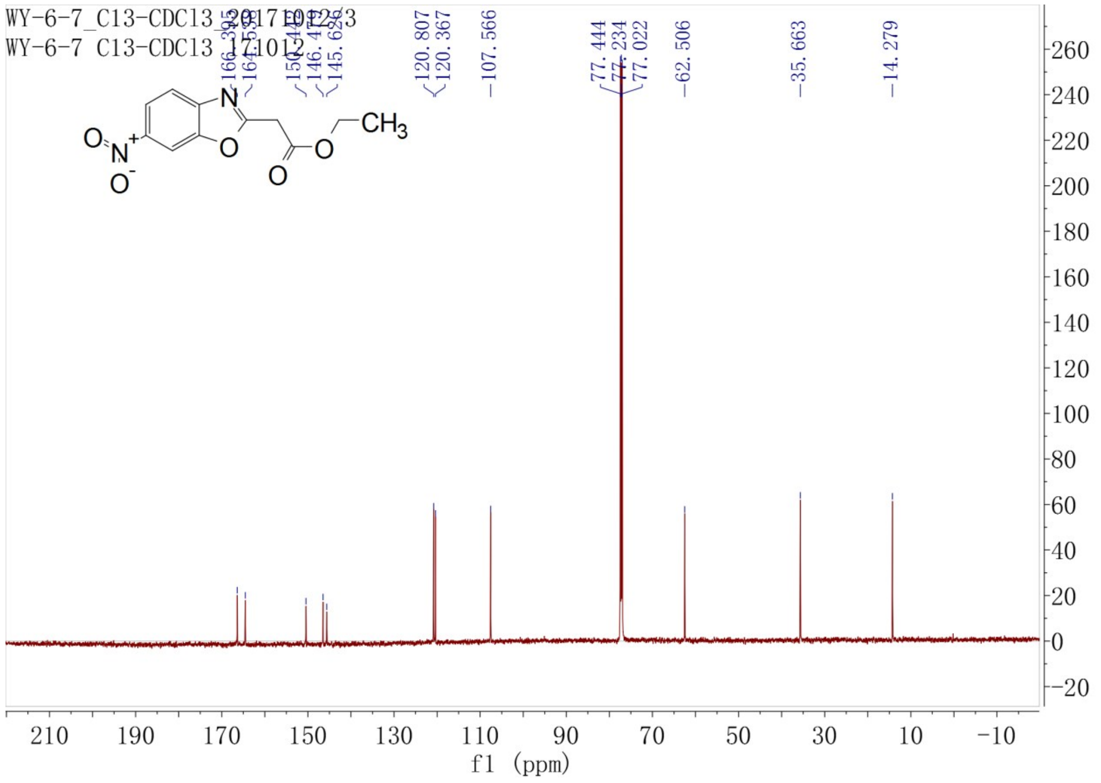
WY-6-7_C13-CDCl3
120.807
120.367
107.566
77.444
77.022
62.506
35.663
14.279
CH3
N
O
O
O
N+
O-
f1 (ppm)

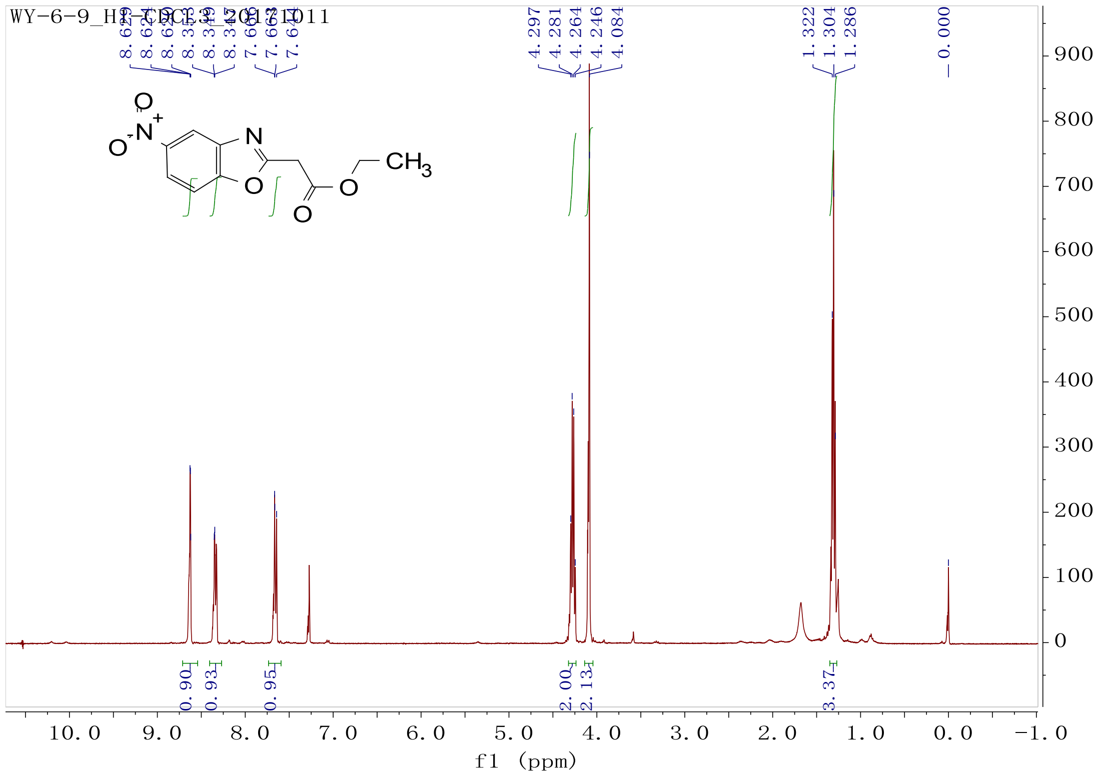

WY-6-9_H1-CDCl3_20171011
4.297
4.281
4.264
4.246
4.084
1.322
1.304
1.286
0.000
0.90
0.93
0.95
2.00
2.13
3.37
f1 (ppm)

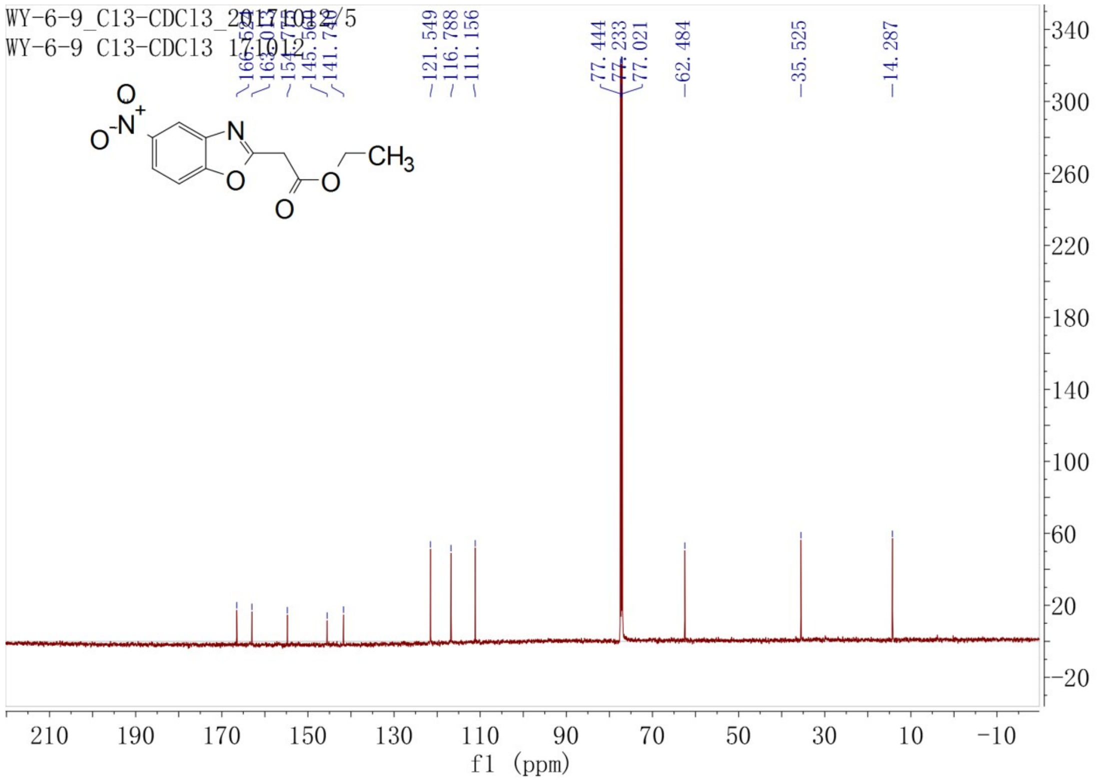

WY-6-9 C13-CDCl3 171012
121.549
116.788
111.156
77.444
77.021
62.484
35.525
14.287
f1 (ppm)

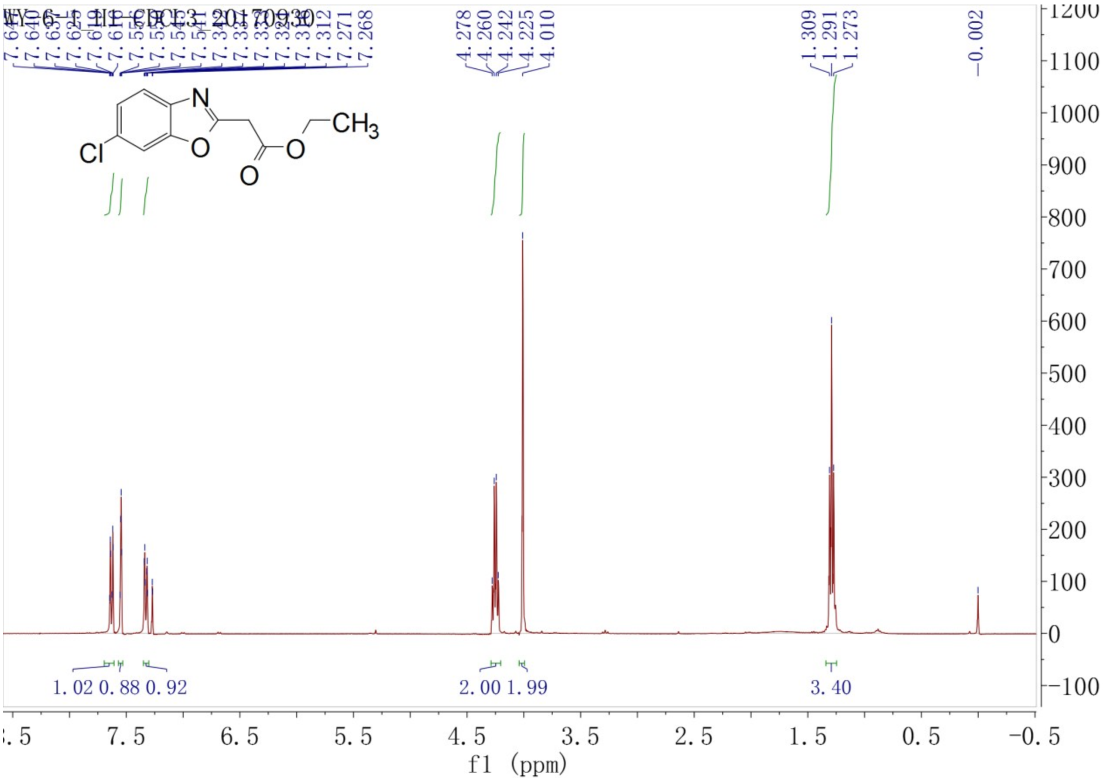

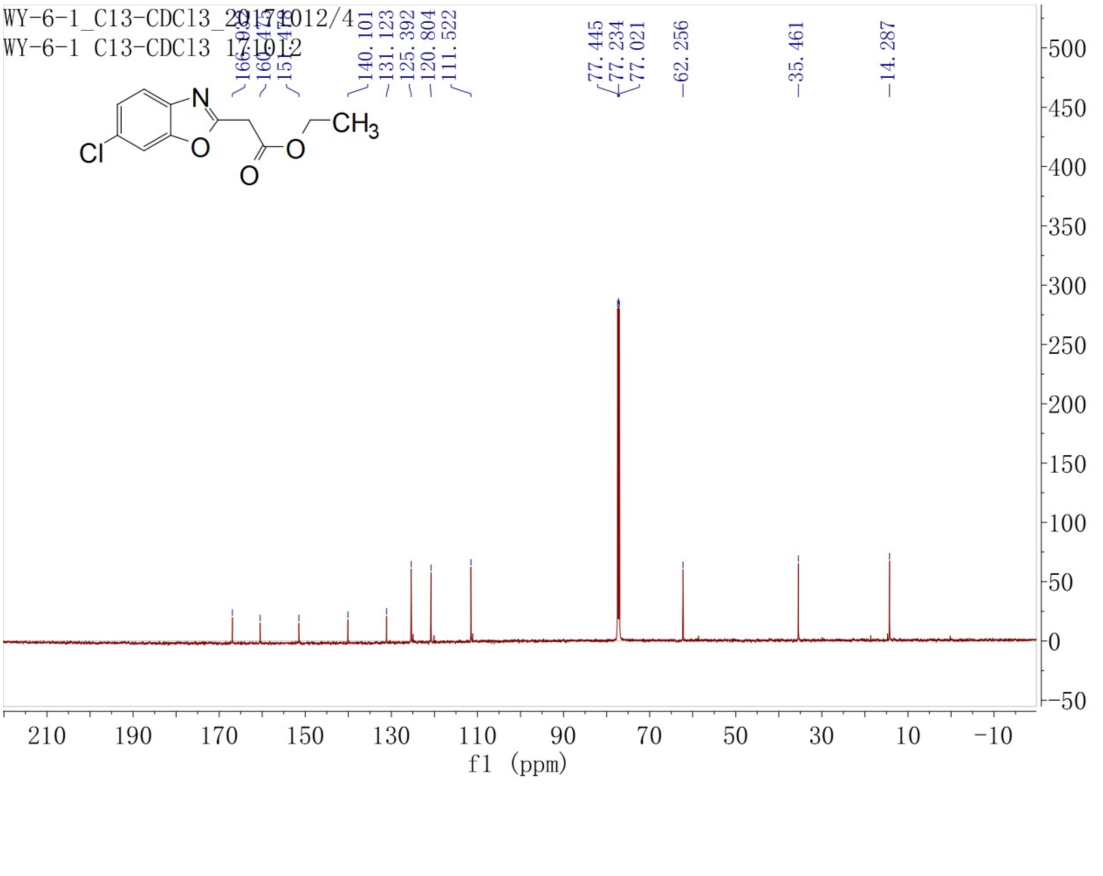

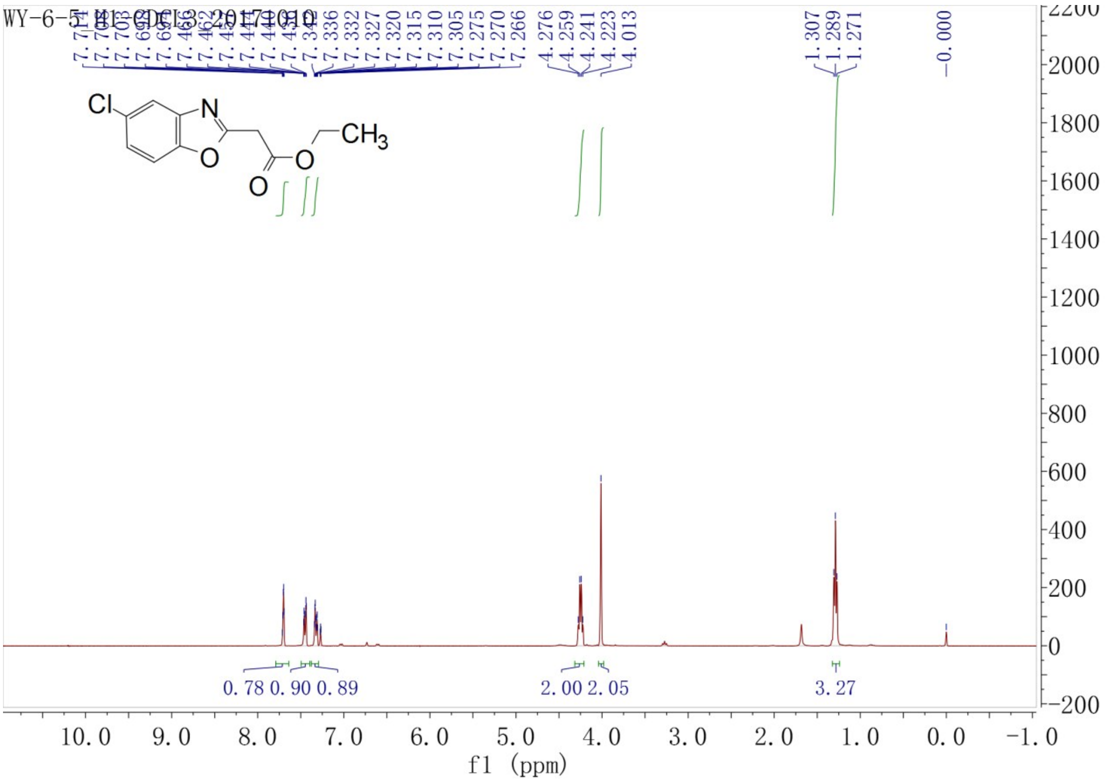
WY-6-5_H1-CDCl3_20171010
Cl
N
O
O
O
CH3
1.307
1.289
1.271
-0.000
0.78
0.90
0.89
2.00
2.05
3.27
f1 (ppm)

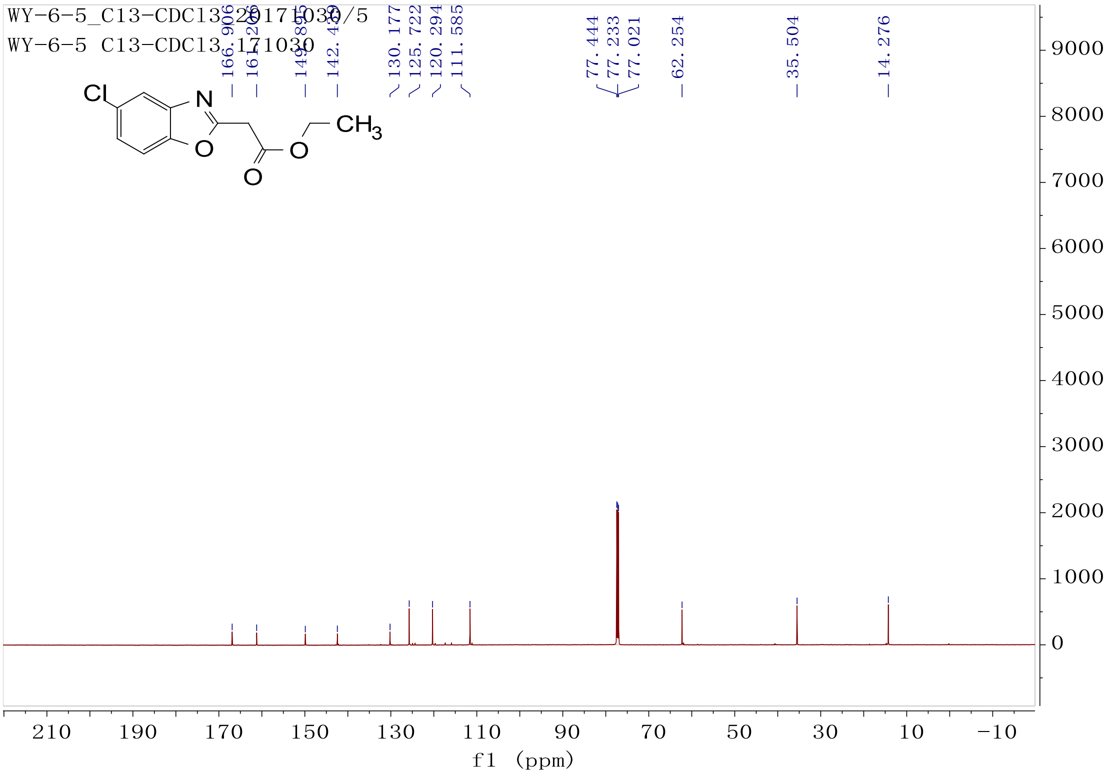
WY-6-5_C13-CDCl3_20171030/5
WY-6-5 C13-CDCl3 171030
130.177
125.722
120.294
111.585
77.444
77.233
77.021
62.254
35.504
14.276
Cl
N
O
O
O
CH3
f1 (ppm)

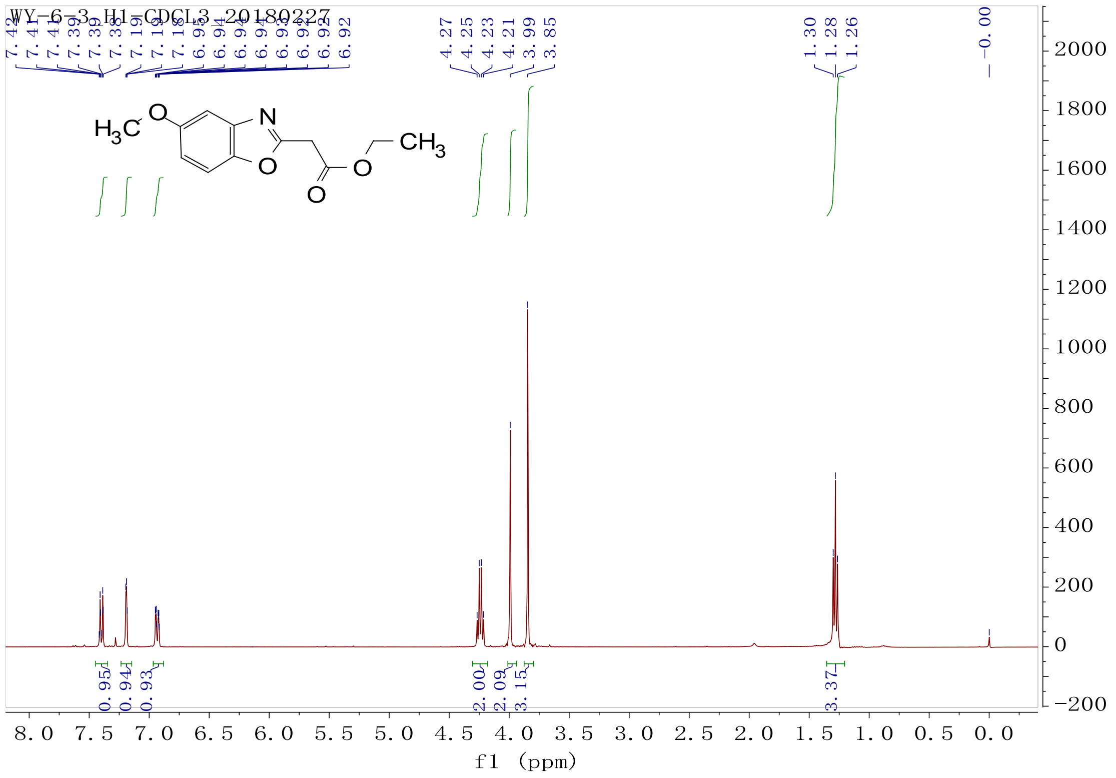
WY-6-3_H1-CDCL3_20180227
f1 (ppm)

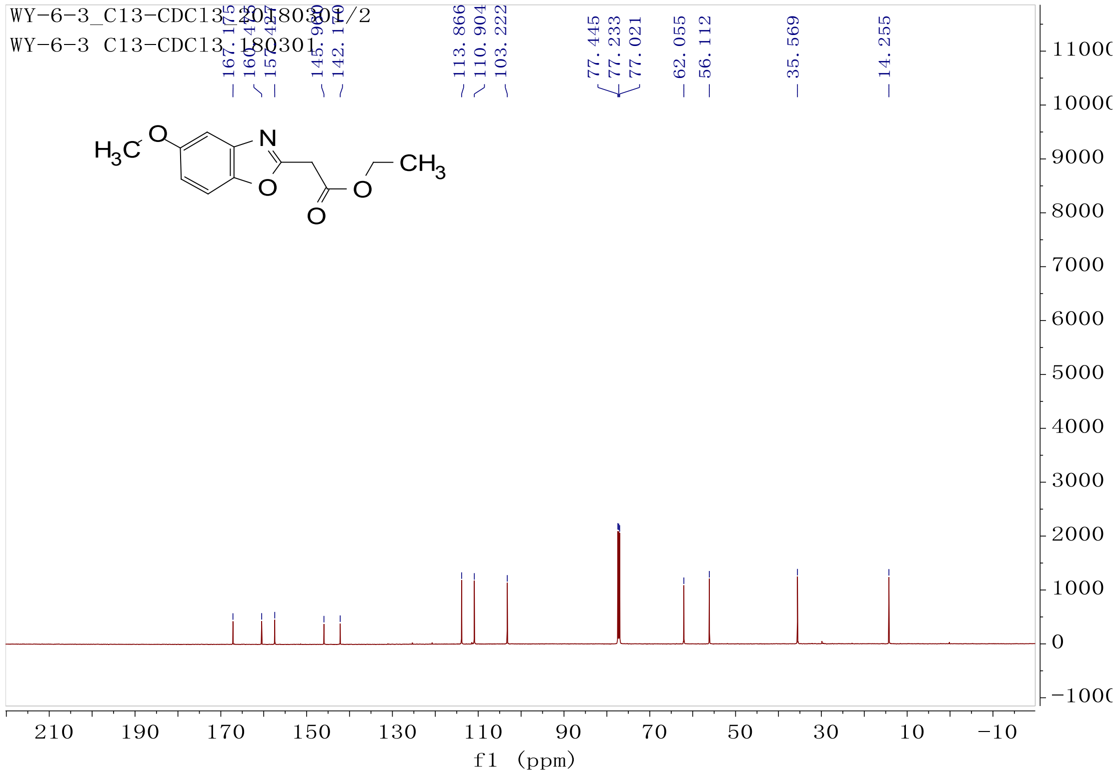
WY-6-3_C13-CDCl3_20180301/2
WY-6-3 C13-CDCl3 180301
113.866
110.904
103.222
77.445
77.233
77.021
62.055
56.112
35.569
14.255
f1 (ppm)

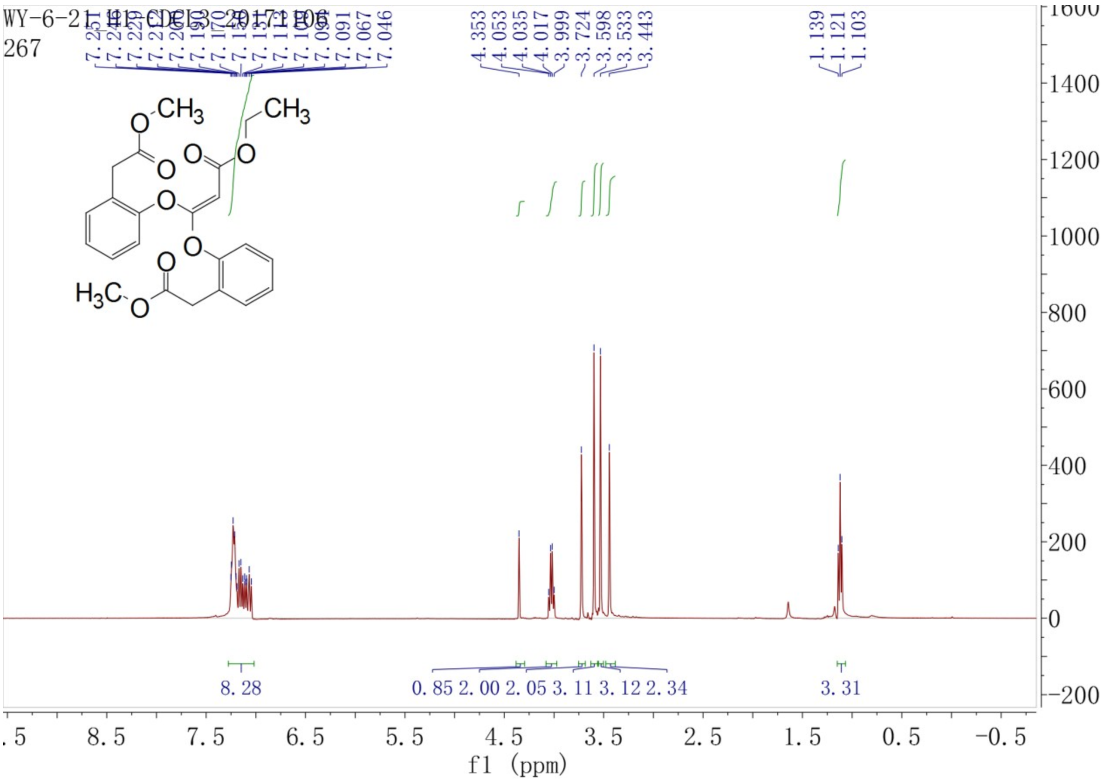

WY-6-21_H1-CDCl3_20171106
267
f1 (ppm)
8.28
0.85 2.00 2.05 3.11 3.12 2.34
3.31

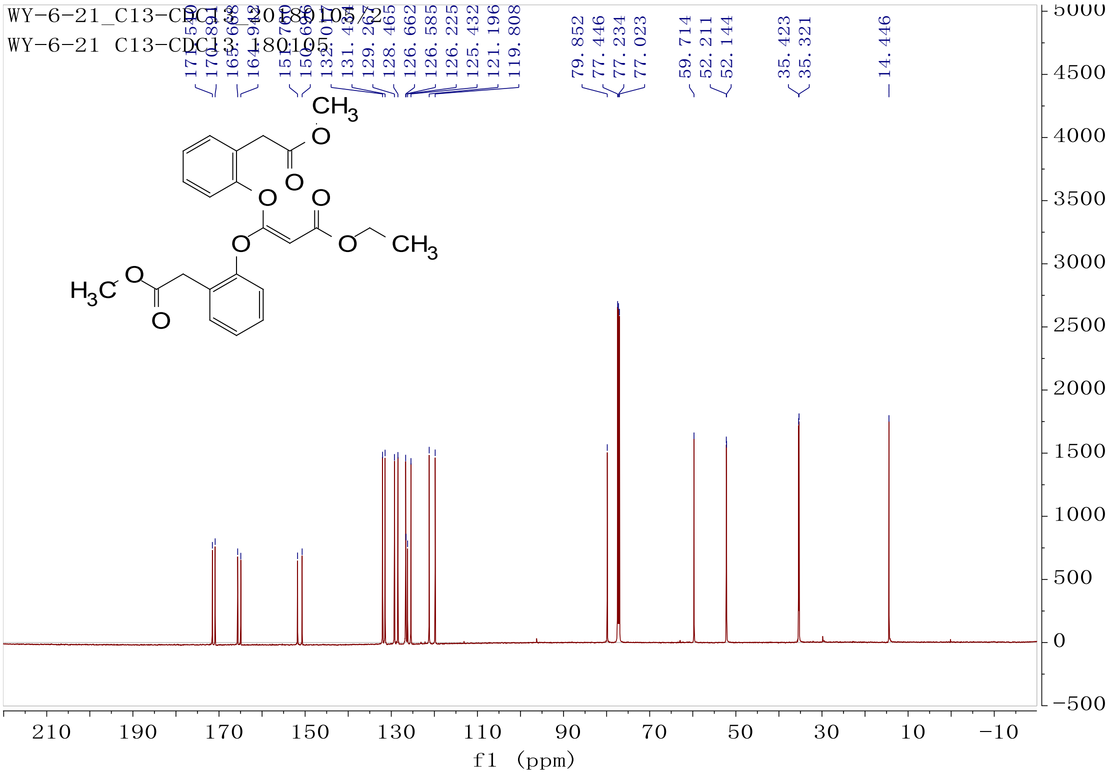

WY-6-21_C13-CDCl3_20180105
79.852
77.446
77.234
77.023
59.714
52.211
52.144
35.423
35.321
14.446
f1 (ppm)

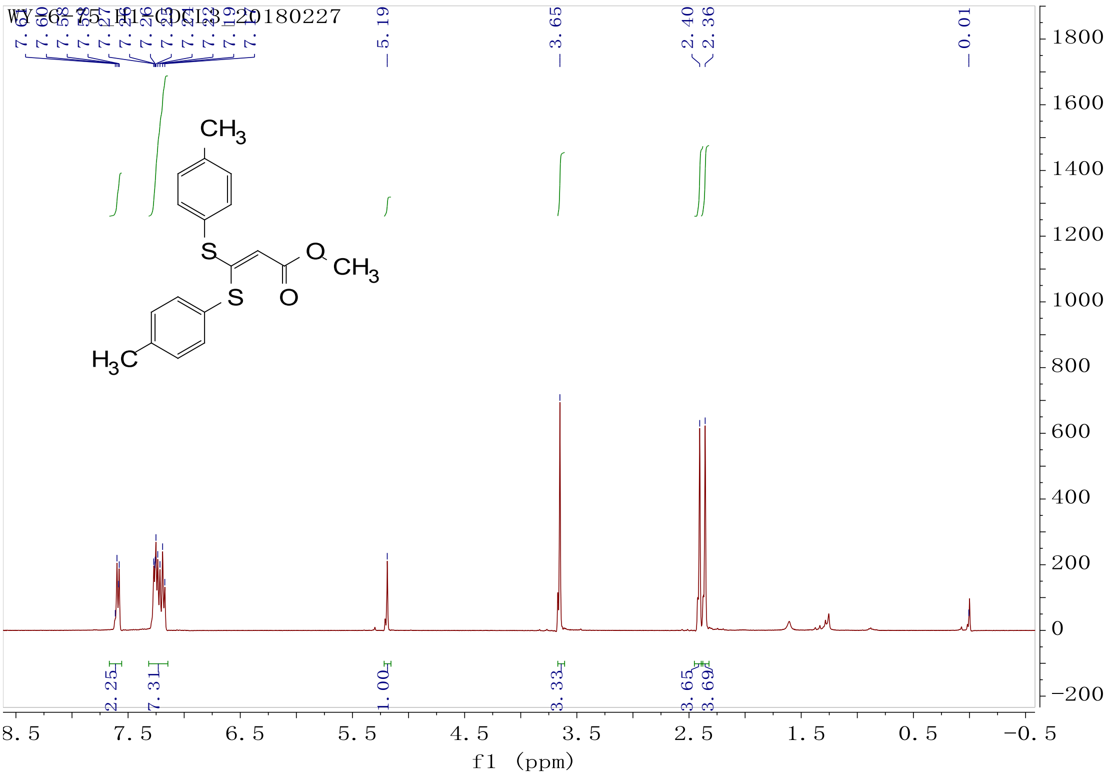
WY-6-75_H1-CDCl3_20180227
5.19
3.65
2.40
2.36
0.01
CH3
S
O
CH3
S
O
H3C
2.25
7.31
1.00
3.33
3.65
3.69
f1 (ppm)

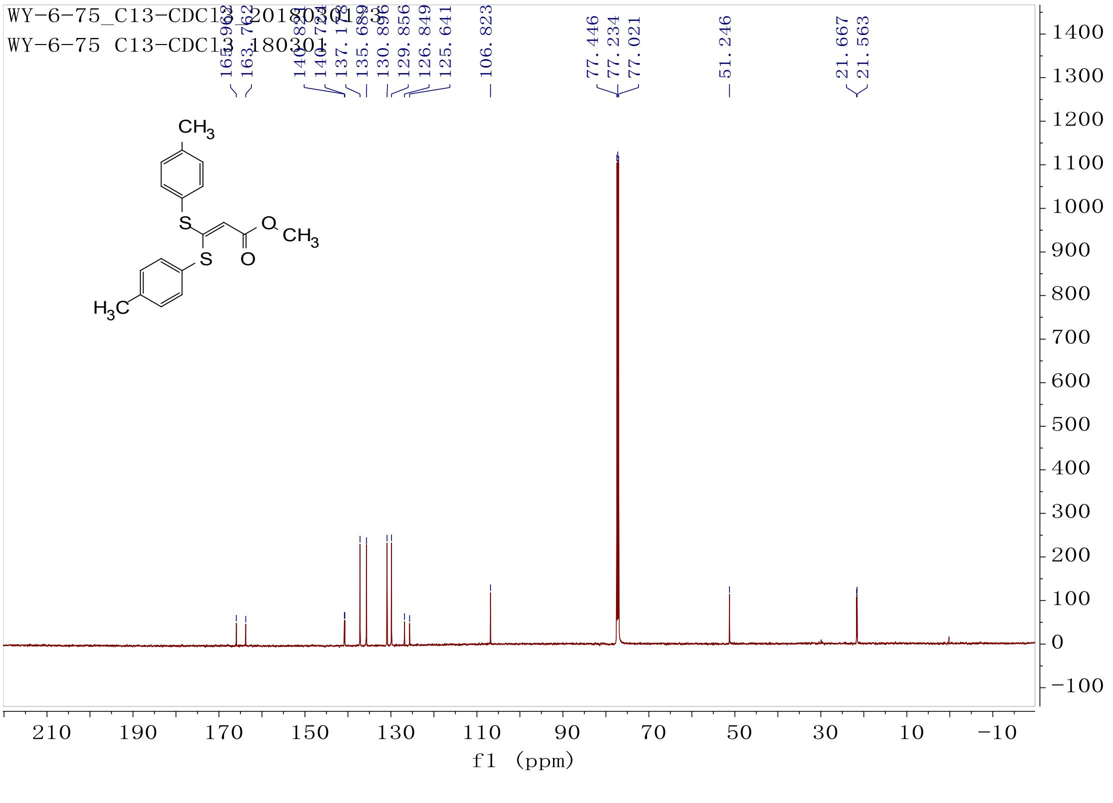
WY-6-75_C13-CDCl3_20180301
163.762
106.823
77.446
77.234
77.021
51.246
21.667
21.563
CH3
S
O
CH3
S
O
H3C
f1 (ppm)
